# IRAK4 constrains cellular plasticity during chemically-induced cell fate reprogramming into multiple lineages

**DOI:** 10.1038/s44319-026-00855-9

**Published:** 2026-07-15

**Authors:** Chuanshu Huang, Xiaoyun Han, Tao Wang, Yang Zhao, Jun Li

**Affiliations:** 1 Zhejiang Provincial Key Laboratory of Medical Genetics, Key Laboratory of Laboratory Medicine, Ministry of Education, School of Laboratory Medicine and Life Sciences, Wenzhou Medical University, 325035 Wenzhou, Zhejiang China; 2 Oujiang Laboratory (Zhejiang Lab for Regenerative Medicine, Vision and Brain Health), Wenzhou, Zhejiang 325035 China; 3 State Key Laboratory of Natural and Biomimetic Drugs, Ministry of Education Key Laboratory of Cell Proliferation and Differentiation, Beijing Advanced Center of Cellular Homeostasis and Aging-Related Diseases, Institute of Advanced Clinical Medicine, Peking University, Beijing, 100871 China; 4 Key Laboratory of Laboratory Medicine, Ministry of Education, Institute of Genomic Medicine, Wenzhou Medical University, Wenzhou, Zhejiang 325035 China

**Keywords:** Cell Cycle, Chromatin, Transcription & Genomics, Stem Cells & Regenerative Medicine

## Abstract

Chemical reprogramming holds transformative potential for regenerative medicine. However, the regulatory mechanisms governing cell fate transitions are not well understood. Here, we identify Interleukin-1 Receptor-Associated Kinase 4 (IRAK4) as a barrier to multi-lineage reprogramming. Pharmacological inhibition of IRAK4 enhances the reprogramming of mouse embryonic fibroblasts (MEFs) through a chemically activated multi-lineage priming (CaMP) state and extraembryonic endoderm (XEN)-like intermediates, increasing colony formation, and the expression of core XEN regulators (Sox17, Gata4, Sall4, and Foxa2). Genetic knockdown of Irak4 similarly accelerates reprogramming, whereas its overexpression blocks cell fate transitions. IRAK4 inhibition enhances chromatin accessibility and reshapes cell cycle dynamics, characterized by G0/G1 shortening and G2/M lengthening, potentially contributing to multi-lineage state establishment. Furthermore, IRAK4 suppression enhances the direct conversion of MEFs to neuron-like and hepatocyte-like cells, which exhibit enhanced functional maturity, including increased glycogen storage and improved detoxification capacity. Our findings establish IRAK4 as a regulator that constrains cellular plasticity potentially by coordinating chromatin accessibility and cell cycle dynamics.

## Synopsis

IRAK4 is a barrier to chemical reprogramming and lineage conversion. Pharmacological or genetic inhibition of IRAK4 enhances multi-lineage priming, accelerates reprogramming, and improves the functional maturity of directly converted neuron-like and hepatocyte-like cells.

- IRAK4 inhibition promotes the chemical reprogramming of mouse embryonic fibroblasts through a CaMP state and XEN-like intermediates, increasing colony formation and the expression of core XEN regulators (Sox17, Gata4, Sall4, Foxa2).
- IRAK4 suppression enhances chromatin accessibility and reshapes cell cycle dynamics, which may facilitate the establishment of multi-lineage states.
- Inhibition of IRAK4 boosts direct conversion to neuron-like and hepatocyte-like cells.

## Abstract (web summary)

IRAK4 is a barrier to chemical reprogramming and lineage conversion. Pharmacological or genetic inhibition of IRAK4 enhances multi-lineage priming, accelerates reprogramming, and improves the functional maturity of directly converted neuron-like and hepatocyte-like cells.

## Graphical abstract

**Figure:**
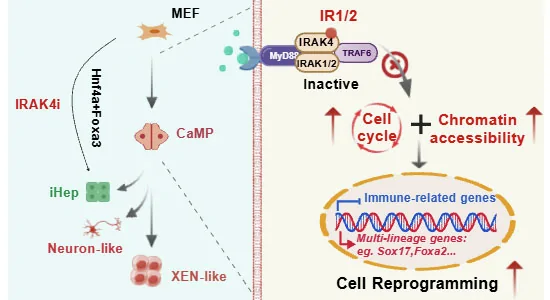

**Figure:**
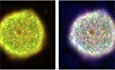

## Introduction

The chemical induction of somatic cells into pluripotent stem cells (CiPSCs) or lineage-specific cell types has emerged as a transformative strategy for regenerative medicine, bypassing ethical and technical limitations of traditional approaches (Guan et al, 2022; Hou et al, 2013; Wang et al, 2023; Yamanaka, 2020; Zhao et al, 2015). Over the past decade, this field has evolved through the replacement of transcription factors (e.g., Oct4/Sox2/Klf4/c-Myc) with small molecules, culminating in fully chemically defined reprogramming protocols (Hou et al, 2013; Ichida et al, 2009b; Maherali and Hochedlinger, 2009; Takahashi and Yamanaka, 2006; Zhu et al, 2010). During mouse embryonic fibroblast (MEF) reprogramming, cells traverse a chemically activated multi-lineage priming (CaMP) state (Yang et al, 2022), transiently adopt extraembryonic endoderm (XEN) -like features, and ultimately achieve pluripotency (Li et al, 2017; Zhao et al, 2018; Zhao et al, 2015). Similar paradigms have been extended to human CiPSC generation, either via or bypassing XEN-like intermediates (Guan et al, 2022; Liuyang et al, 2023; Wang et al, 2025), marking a critical milestone toward clinical translation.

Chemical reprogramming acts by modulating endogenous pathways, including epigenetic remodeling (Barrero et al, 2010; Cao et al, 2018; Cherry and Daley, 2012; Huangfu et al, 2008; Nicetto and Zaret 2019; Wang, 2011), signaling cascades(Ichida et al, 2009a; Lin et al, 2009; Lyssiotis et al, 2009; Silva et al, 2008), metabolic reprogramming (Prigione et al, 2010), and senescence suppression (Banito et al, 2009; Esteban et al, 2010; Wang, 2011). However, low reprogramming efficiency remains a persistent challenge, largely attributed to unresolved epigenetic and signaling barriers that reinforce somatic identity and restrict cellular plasticity. For instance, CDK8 kinase maintains somatic cell identity by repressing lineage-priming transcription factors (Yang et al, 2020). CDK8 inhibition accelerates the MEF-CaMP-XEN-like-CiPSC transition and enhances direct trans-differentiation into hepatocytes or adipocytes (Li et al, 2023). Similarly, H3K9me3, a repressive histone mark deposited by SETDB1, silences endogenous retroviruses (ERVs) linked to pluripotency (Wang et al, 2018). Genetic inhibition of SETDB1 accelerates murine reprogramming by alleviating ERV repression (Chen et al, 2023). Additional barriers arise from hyperacetylation of H3K27 at key loci by histone acetyltransferases KAT3A/KAT3B and KAT6A, which stabilize somatic gene networks. Inhibiting these enzymes rebalances the epigenome—reducing H3K27ac and elevating H3K27me3—to enable highly efficient human CiPSC generation (Wang et al, 2025).

Despite these advances, reprogramming kinetics and efficiency remain suboptimal. Activation of endogenous late pluripotency or lineage-defining genes represents the principal rate-limiting step. Addressing these barriers requires dissection of the molecular mechanisms governing cellular plasticity. Here, we identify IRAK4, a kinase implicated in innate immune signaling, as a novel regulator of multi-lineage induction. We hypothesized that IRAK4 functions as a common barrier restricting cellular plasticity across different reprogramming routes. To test this, we investigated the effect of IRAK4 modulation not only on the path to pluripotency but also on direct lineage conversion to neurons and hepatocytes. Pharmacological inhibition of IRAK4 (using IRAK inhibitor 1 and IRAK4-IN-1) promotes the MEF-CaMP-XEN-like transition by enhancing chromatin accessibility and modulating cell cycle dynamics: shortening G0/G1 and elongating G2/M phases during reprogramming. Genetic validation confirms that IRAK4 knockdown enhances reprogramming efficiency, while IRAK4 overexpression impedes it. Notably, IRAK4 inhibition also facilitates direct MEF-to-neurons and MEF-to-hepatocyte conversion, underscoring its context-dependent role as a barrier to cell plasticity across multiple reprogramming contexts. These findings reveal IRAK4 as a druggable target to refine chemical reprogramming protocols and improve regenerative outcomes.

## Results

### IRAK4 inhibitors facilitate the initiation of MEF-to-XEN reprogramming

**Figure 1:**
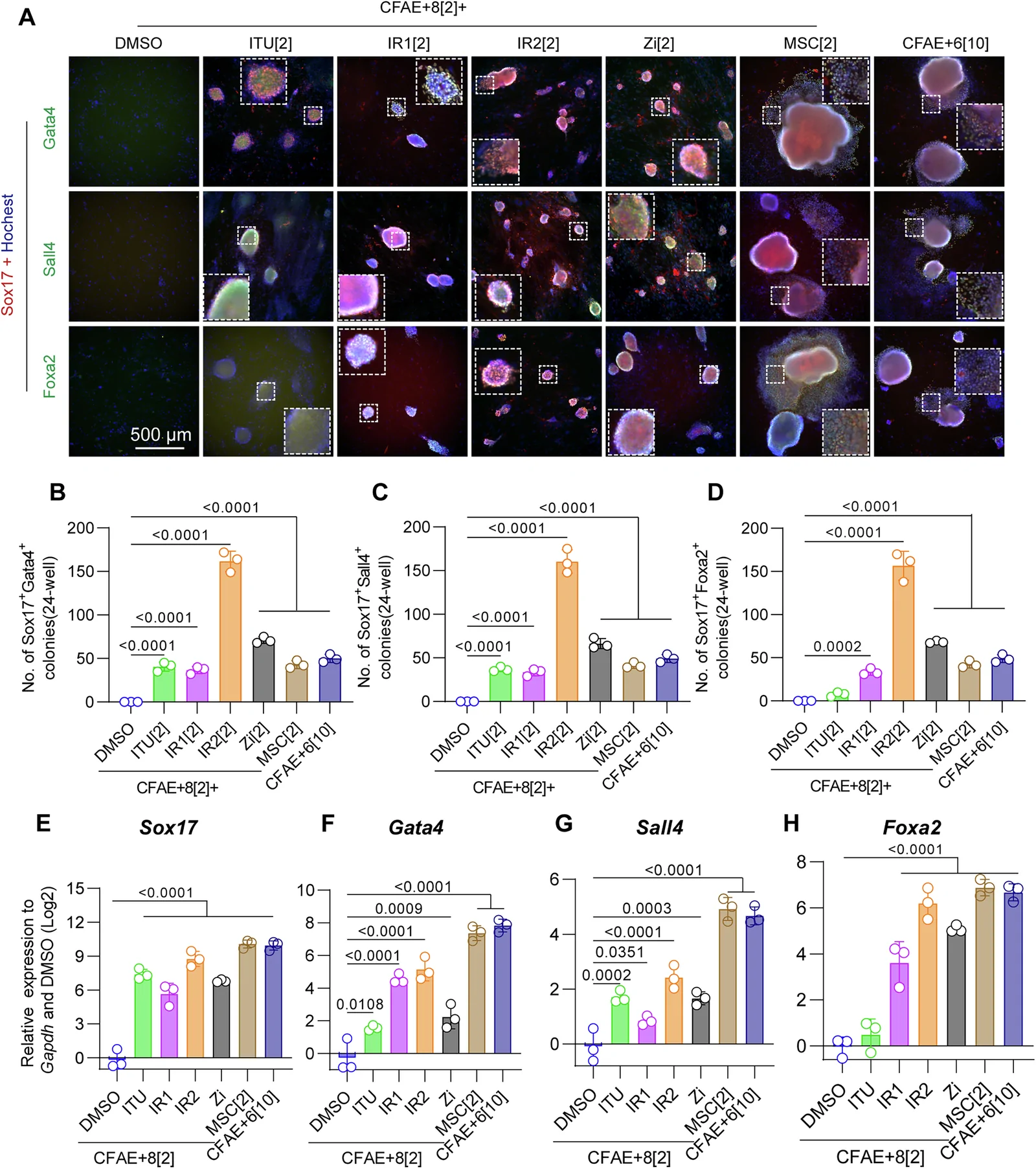
IRAK4 inhibitors facilitate the initiation of MEF-to-XEN reprogramming. (**A**) Immunofluorescence co-staining of Sox17 and Gata4, Sall4 or Foxa2 in CFAE + 8 plus ITU/IR1/IR2/Zi/MSC or CFAE + 6 medium for 12 days, respectively. (C CHIR99021, F Forskolin, A AM580, E EPZ04777, 6 E-616452, 8 A-83-01, ITU 5-Iodotubercidin, IR1 IRAK inhibitor 1, IR2 IRAK4-IN-1, Zi Zimlovisertib, MSC MSC2530818). Scale bar: 500 μm. Numbers in the bracket are the concentrations (μM) of each small molecule. The same raw images are shown separately in Fig. EV1C–E to provide detailed views. (**B**–**D**) The number of Sox17^+^Gata4^+^, Sox17^+^Sall4^+^, and Sox17^+^Foxa2^+^ colonies in (**A**). Data were represented as mean ± SD. Numbers in each panel are *P* values between DMSO and indicated samples (*n* = 3 biological replicates, respectively). DMSO vs. ITU[2]: *p* = 3.1e-6; DMSO vs. IR1[2]: *p* = 1.1e-5; DMSO vs. IR2[2]: *p* = 4.0e-14; DMSO vs. Zi[2]: *p* = 2.8e-9; DMSO vs. MSC[2]: *p* = 1.6e-6; DMSO vs. CFAE + 6[10]: *p* = 2.5e-7 in (**B**). DMSO vs. ITU[2]: *p* = 2.3e-5; DMSO vs. IR1[2]: *p* = 6.5e-5; DMSO vs. IR2[2]: *p* = 1.3e-13; DMSO vs. Zi[2]: *p* = 1.9e-8; DMSO vs. MSC[2]: *p* = 6.6e-6; DMSO vs. CFAE + 6[10]: *p* = 8.0e-7 in (**C**). DMSO vs. ITU[2]: *p* = 0.6; DMSO vs. IR1[2]: *p* = 0.0002; DMSO vs. IR2[2]: *p* = 7.3e-13; DMSO vs. Zi[2]: *p* = 4.9e-8; DMSO vs. MSC[2]: *p* = 1.6e-5; DMSO vs. CFAE + 6[10]: *p* = 3.0e-6 in (**D**). (**E**–**H**) Quantitative PCR for *Sox17*, *Gata4*, *Sall4*, and *Foxa2* in CFAE + 8 plus ITU/IR1/IR2/Zi/MSC or CFAE + 6 medium for 12 days, respectively. Data were represented as mean ± SD. Numbers in each panel are *P* values between DMSO and indicated samples (*n* = 3 biological replicates, respectively). DMSO vs. ITU: *p* = 2.1e-9; DMSO vs. IR1: *p* = 6.4e-8; DMSO vs. IR2: *p* = 2.3e-10; DMSO vs. Zi: *p* = 6.0e-9; DMSO vs. MSC[2]: *p* = 3.6e-11; DMSO vs. CFAE + 6[10]: *p* = 4.2e-11 in (**E**). DMSO vs. ITU: *p* = 0.0108; DMSO vs. IR1: *p* = 6.3e-7; DMSO vs. IR2: *p* = 1.5e-7; DMSO vs. Zi: *p* = 9.0e-4; DMSO vs. MSC[2]: *p* = 1.7e-9; DMSO vs. CFAE + 6[10]: *p* = 7.7e-10 in (**F**). DMSO vs. ITU: *p* = 0.0002; DMSO vs. IR1: *p* = 0.0351; DMSO vs. IR2: *p* = 4.5e-6; DMSO vs. Zi: *p* = 0.0003; DMSO vs. MSC[2]: *p* = 7.1e-10; DMSO vs. CFAE + 6[10]: *p* = 1.4e-9 in (**G**). DMSO vs. ITU: *p* = 0.7371; DMSO vs. IR1: *p* = 8.6e-6; DMSO vs. IR2: *p* = 1.1e-8; DMSO vs. Zi: *p* = 1.4e-7; DMSO vs. MSC[2]: *p* = 2.9e-9; DMSO vs. CFAE + 6[10]: *p* = 4.2e-9 in (**H**). For (**B**–**H**), *P* values were calculated using one-way ANOVA followed by Dunnett multiple comparisons test. Source data are available online for this figure.

**Figure EV1:**
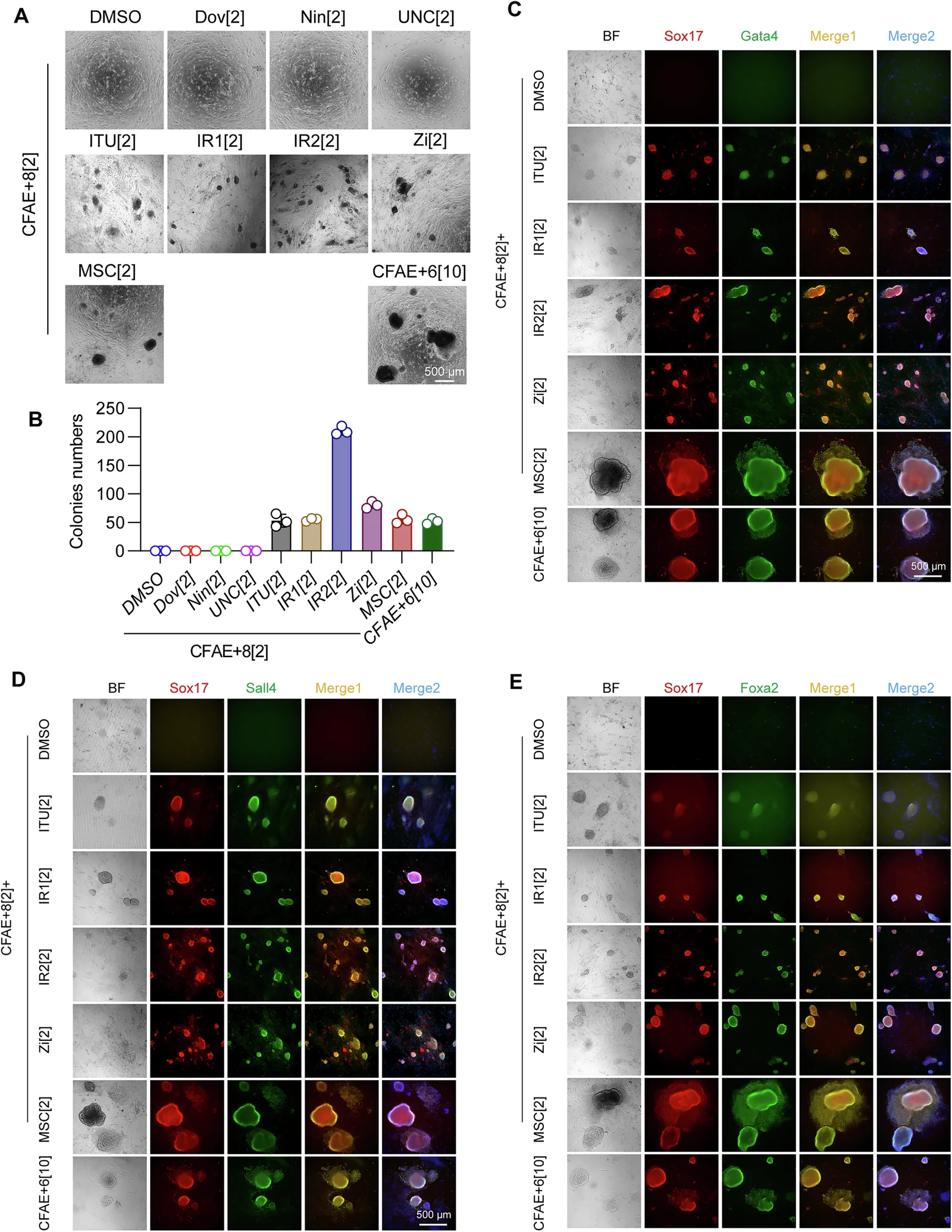
Identifying the candidate chemicals from the DRUG-seq2 screen that initiate MEF-XEN reprogramming. (**A**) The morphologies of colonies in CFAE + 8 plus candidate chemicals from the DRUG-seq2 screen or in CFAE + 6 on day 12. (C CHIR99021, F Forskolin, A AM580, E EPZ04777, 6 E-616452, 8 A-83-01, Dov dovitinib lactate, Nin nintedanib esylate, UNC UNC2025, ITU 5-iodotubercidin, IR1 IRAK inhibitor 1, IR2 IRAK4-IN-1, Zi zimlovisertib, MSC MSC2530818). (**B**) Quantifying colony numbers in (**A**). Data were represented as mean ± SD. *n* = 3 biological replicates, respectively. (**C**–**E**) Individual raw images and merged panel related to Fig. 1A. Immunofluorescence staining of XEN master regulators Sox17, Gata4, Sall4, and Foxa2 in CFAE + 8 plus ITU/IR1/IR2/Zi/MSC or CFAE + 6, respectively. Scale bar: 500 μm. Source data are available online for this figure.

To identify molecular barriers in chemical reprogramming, we previously developed a transcriptome profiling-based screening platform, Drug-seq2, which was utilized to identify MSC2530818, a CDK8 inhibitor, as a potent enhancer of reprogramming via screening a kinase inhibitor library (Li et al, 2023). Intriguingly, several other candidate chemicals (e.g., Iodotubercidin, IRAK inhibitors 1/2) also exhibited the potential to induce a CaMP or XEN-like transcriptional profile. However, the functional roles and underlying mechanisms of such chemicals in reprogramming remained unexplored. We first validated the top-ranked candidates. Remarkably, the combined application of A-83-01 (8) with only 5-Iodotubercidin (ITU), IRAK inhibitor 1 (IR1), IRAK4-IN-1 (IR2), or Zimlovisertib (Zi), respectively induced colony formation, with CFAE + 6 as a positive control (Figs. 1A and EV1A,B). Notably, IR2 induced the most colony numbers, even exceeding the yield induced by MSC2530818. Conversely, other compounds like Dovitinib lactate (Dov), Nintedanib esylate (Nin), and UNC2025 (UNC) failed to generate colonies (Fig. EV1B). Immunofluorescence (IF) and quantitative PCR (qPCR) analyses demonstrated that colonies induced by IR1, IR2, or Zi exhibited robust co-expression of key XEN-like markers: Sox17, Gata4, Sall4, and Foxa2. Quantification of marker-positive colonies revealed IR2 to be the most efficient inducer of Sox17^+^Gata4^+^, Sox17^+^Sall4^+^, and Sox17^+^Foxa2^+^ double-positive colonies (Fig. 1B–D). The efficiencies of IR1 and Zi were comparable to the positive controls of MSC2530818 and E-616452 (Fig. 1A–D). In contrast, clones generated by ITU displayed markedly low transcript levels of Sall4 and Foxa2 and numbers of Sox17^+^Foxa2^+^ double-positive colonies (Figs. 1A–H and EV1C–E).

**Figure 2:**
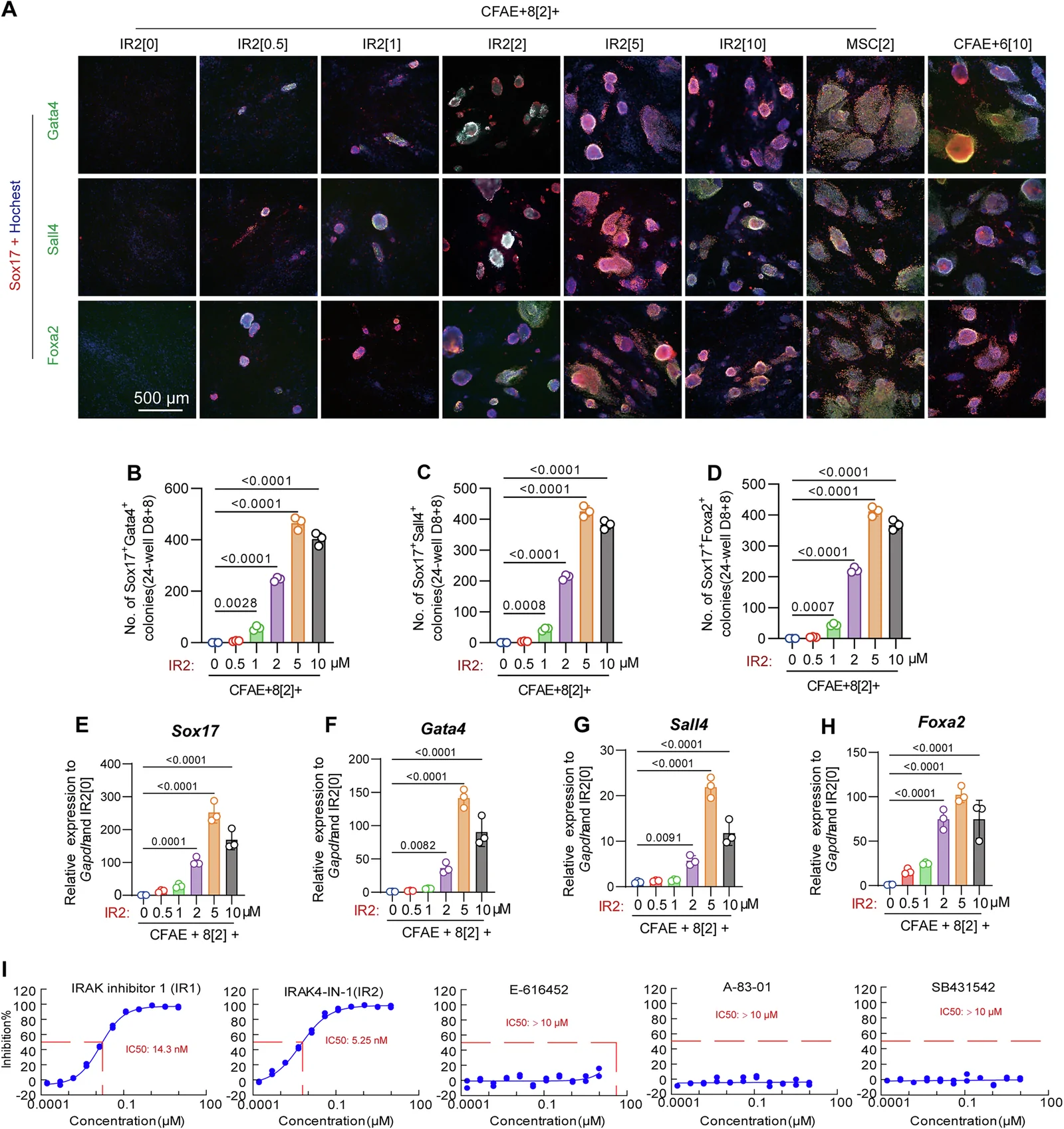
Optimal concentration determination of IR2 during MEF-XEN reprogramming. (**A**) Immunofluorescence co-staining of Sox17 and Gata4, Sall4 or Foxa2 under CFAE + 8 with a series of doses of IR2 or CFAE + 6 medium induction for 12 days, respectively. Scale bar: 500 μm. Numbers in the brackets are the concentrations (μM) of IR2. The same raw images are shown separately in Appendix Fig. S2 to provide detailed views. (**B**–**D**) The number of Sox17^+^Gata4^+^, Sox17^+^Sall4^+^, and Sox17^+^Foxa2^+^ colonies in (**A**). Data were represented as mean ± SD. Numbers in each panel are *P* values between the control and indicated samples (*n* = 3 biological replicates, respectively). 0 vs. 0.5: *p* = 0.9738; 0 vs. 1: *p* = 0.0028; 0 vs. 2: *p* = 6.9e-10; 0 vs. 5: *p* = 3.9e-13; 0 vs. 10: *p* = 2.1e-12 in (**B**). 0 vs. 0.5: *p* = 0.9819; 0 vs. 1: *p* = 8.0e-4; 0 vs. 2: *p* = 2.9e-11; 0 vs. 5: *p* = 5.6e-15; 0 vs. 10: *p* = 2.5e-14 in (**C**). 0 vs. 0.5: *p* = 0.9669; 0 vs. 1: *p* = 7.0e-4; 0 vs. 2: *p* = 1.9e-11; 0 vs. 5: *p* = 9.9e-15; 0 vs. 10: *p* = 4.4e-14 in (**D**). (**E**–**H**) Quantitative PCR for *Sox17*, *Gata4*, *Sall4*, and *Foxa2* in samples of (**A**), respectively. Data were represented as mean ± SD. Numbers in each panel are *P* values between DMSO and indicated samples (*n* = 3 biological replicates, respectively). 0 vs. 0.5: *p* = 0.8957; 0 vs. 1: *p* = 0.3103; 0 vs. 2: *p* = 1.0e-4; 0 vs. 5: *p* = 6.7e-9; 0 vs. 10: *p* = 6.1e-7 in (**E**). 0 vs. 0.5: p > 0.999; 0 vs. 1: *p* = 0.9793; 0 vs. 2: *p* = 0.0082; 0 vs. 5: *p* = 1.1e-8; 0 vs. 10: *p* = 1.7e-6 in (**F**). 0 vs. 0.5: p = 0.99; 0 vs. 1: p = 0.99; 0 vs. 2: *p* = 0.0091; 0 vs. 5: *p* = 4.2e-9; 0 vs. 10: *p* = 6.3e-6 in (**G**). 0 vs. 0.5: *p* = 0.3407; 0 vs. 1: *p* = 0.0661; 0 vs. 2: *p* = 9.1e-6; 0 vs. 5: *p* = 3.0e-7; 0 vs. 10: *p* = 9.1e-6 in (**H**). For (**B**–**H**), *P* values were calculated using one-way ANOVA followed by Dunnett multiple comparisons test. (**I**) IRAK4 activity inhibition assays in vitro for IR1, IR2, E-616452, A-83-01, and SB431542. Source data are available online for this figure.

Next, we established dose-response relationships for IR1 and IR2. Cells were treated in CFAE + 8 reprogramming medium with concentrations ranging from 0.5 to 10 μM. Quantification of Sox17^+^Gata4^+^, Sox17^+^Sall4^+^, and Sox17^+^Foxa2^+^ double-positive colonies, along with validation of XEN marker expression (Fig. 2A–H; Appendix Figs. S1 and S2), revealed an optimal concentration window of 0.5–2 μM for IR1 and 5 μM for IR2. Based on maximal efficacy and marker expression, 2 μM IR1 and 5 μM IR2 were selected for all subsequent experiments.

To elucidate the mechanistic basis for these effects, we systematically elucidated molecular targets for the leading candidates (IR1, IR2, and Zi). Unexpectedly, all three lead candidates were previously characterized as interleukin-1 receptor-associated kinase 4 (IRAK4) inhibitors (Buckley et al, 2008; Lee et al, 2017; Smith et al, 2017; Wang et al, 2009), indicating IRAK4 acts as a barrier to chemical reprogramming. E-616452 is widely recognized to exhibit polypharmacology during chemical reprogramming, including the direct target CDK8(Li et al, 2023). However, CDK8 inhibition alone failed to fully recapitulate the functional outcomes of ALK5 (TGFβ receptor kinase) blockade, the canonical target of E-616452, suggesting the involvement of additional molecular targets. To determine whether IRAK4 might represent another functional target of E-616452, we performed a kinase activity assay to quantify the inhibition of IRAK4 by E-616452, A-83-01, and SB431542, using the IRAK4 inhibitors IR1 and IR2 as positive controls. Our results showed that none of the TGFβ receptor inhibitors (E-616452, A-83-01, or SB431542) could repress IRAK4 activity at all concentrations ranging from 0 to 10 μM, thereby ruling out IRAK4 as a direct target of E-616452. By contrast, both IR1 and IR2 treatment strongly inhibited IRAK4 activity, with respective IC50s of 14.3 and 5.25 nM (Fig. 2I). Collectively, these results suggest that IRAK4 was a previously unrecognized molecular barrier to chemical reprogramming that was independent of E-616452.

### IRAK4 Inhibitors IR1 and IR2 induce a multi-lineage priming state

**Figure 3:**
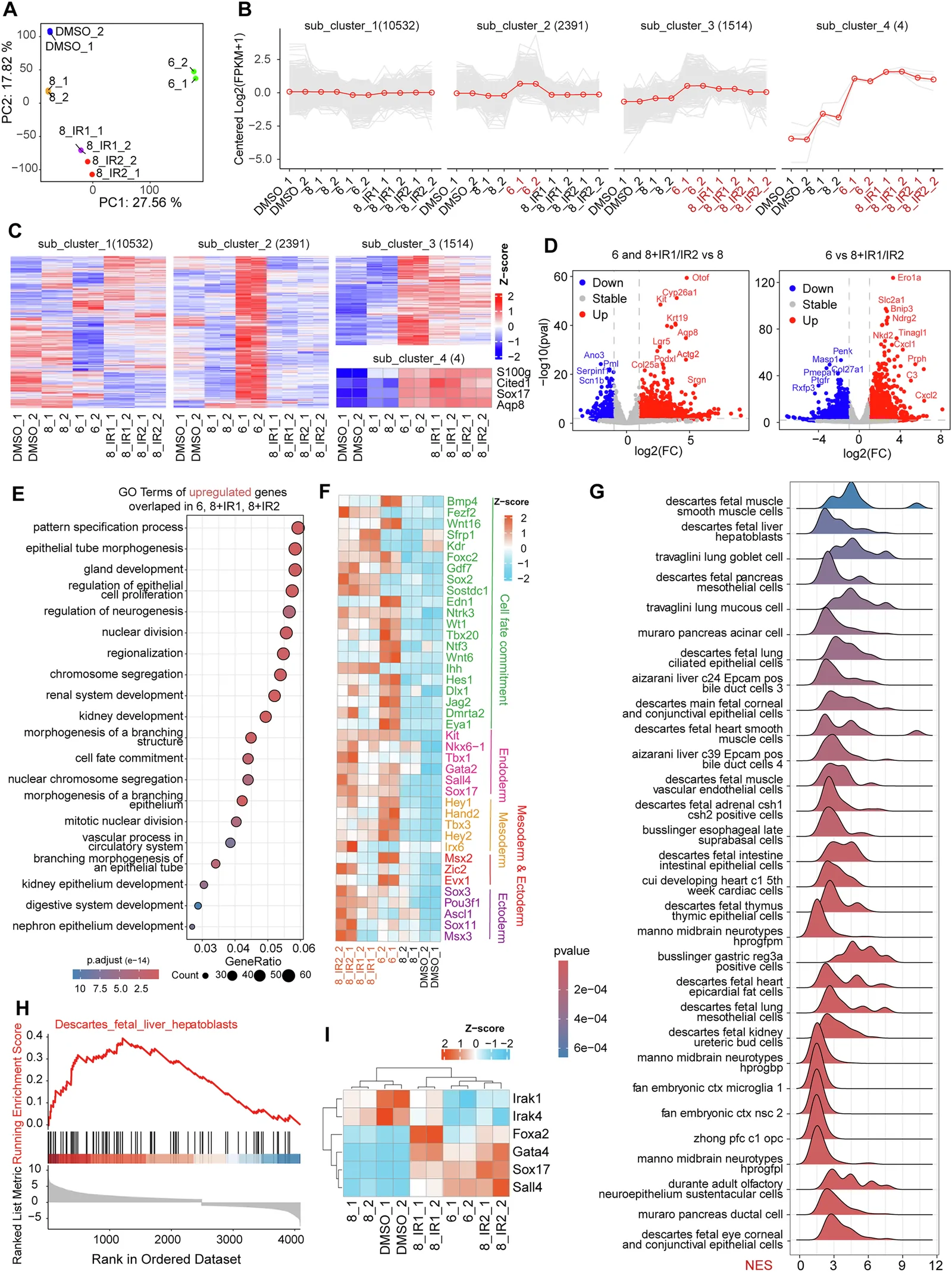
IR1 and IR2 activated a mult-lineage state. (**A**) PCA analysis of RNA-seq data from samples with CFAE plus DMSO/8/6/A8 + IR1/8 + IR2 treatments for 8 days. (**B**, **C**) Expression patterns of four subclusters in RNA-seq data. (**D**) Differentially expressed gene analysis of RNA-seq data (*n* = 2 biological replicates, respectively) from CFAE + 6 and CFAE + 8 + IR1/IR2 versus CFAE + 8 (left panel) or CFAE + 6 versus CFAE + 8 + IR1/IR2 (right panel) with DESeq2. The *P* value was calculated using the Wald test. Cutoff: −log10(*p*val) >2 and |log2(fold change)|>1. Blue represents downregulated genes. Red represents upregulated genes. Gray represents unchanged genes. (**E**) Biological processes (BP) of gene ontology (GO) enrichment in upregulated genes of both CFAE + 6 and CFAE + 8 + IR1/IR2 versus CFAE + 8. The *P* value was calculated using the Hypergeometric test. (**F**) Heatmap of cell fate commitment and three germ layers relative genes in CFAE + 6, CFAE + 8 + IR1/IR2, CFAE + 8, and CFAE + DMSO. (**G**) GSEA enrichment distributions of key upregulated genes in both CFAE + 6 and CFAE + 8 + IR1/IR2. (**H**) GSEA enrichment of fetal liver hepatoblasts fate in both CFAE + 6 and CFAE + 8 + IR1/IR2. (**I**) Heatmap of *Irak1, Irak4, Sox17, Gata4, Sall4*, and *Foxa2* expression levels in CFAE + 6, CFAE + 8 + IR1/IR2, CFAE + 8, and CFAE + DMSO.

**Figure EV2:**
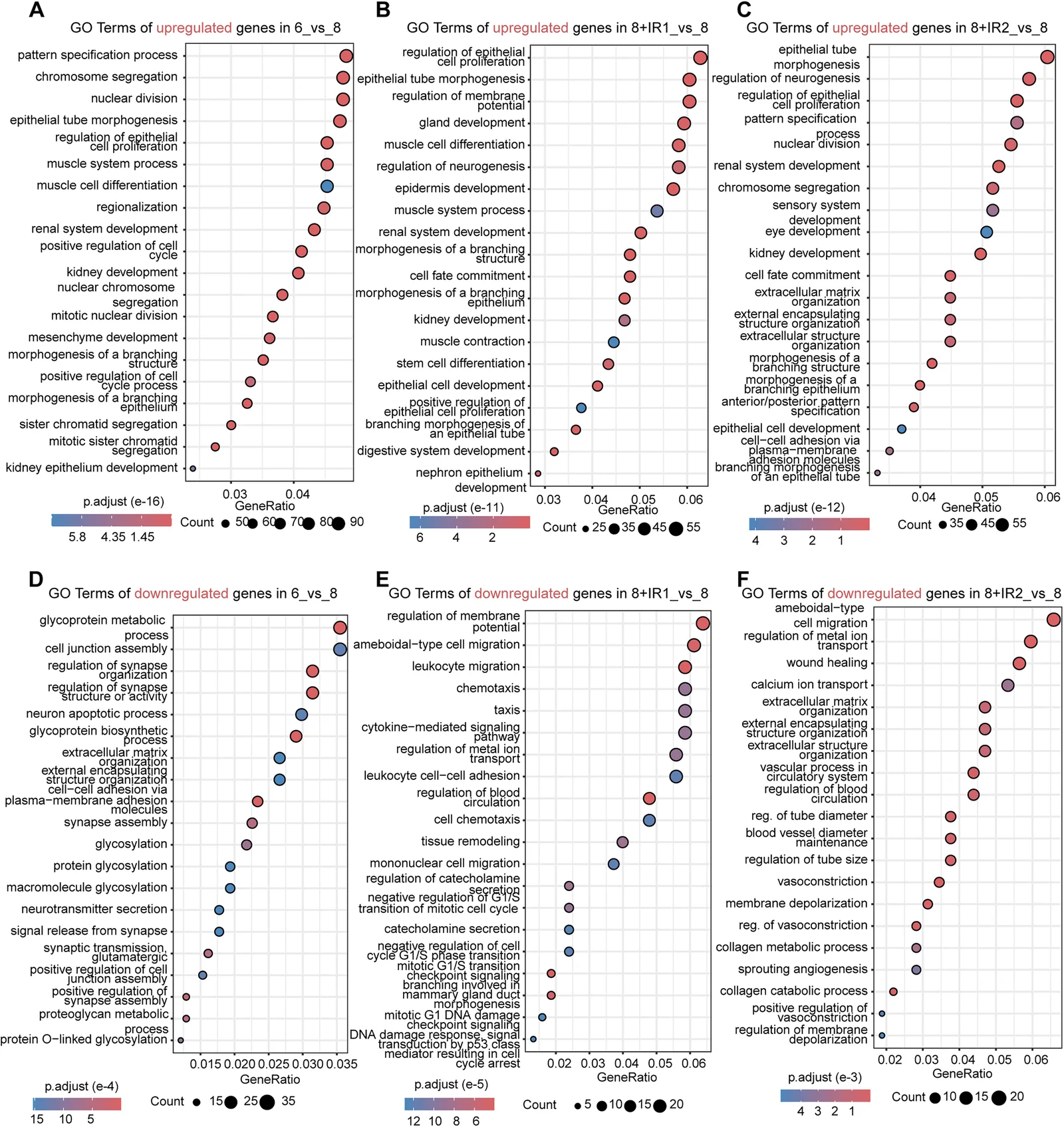
Gene ontology analysis of DEGs in 6, 8 + IR1, or 8 + IR2 versus 8. Biological processes (BP) of gene ontology (GO) enrichment in upregulated (**A**–**C**) or downregulated (**D**–**F**) genes of 6, 8 + IR1, or 8 + IR2 versus 8. The *P* value was calculated using the Hypergeometric test. Basal medium: CFAE. Optimal concentration determination of IR2 during MEF-XEN reprogramming. Related to Figs. 3 and 4.

**Figure EV3:**
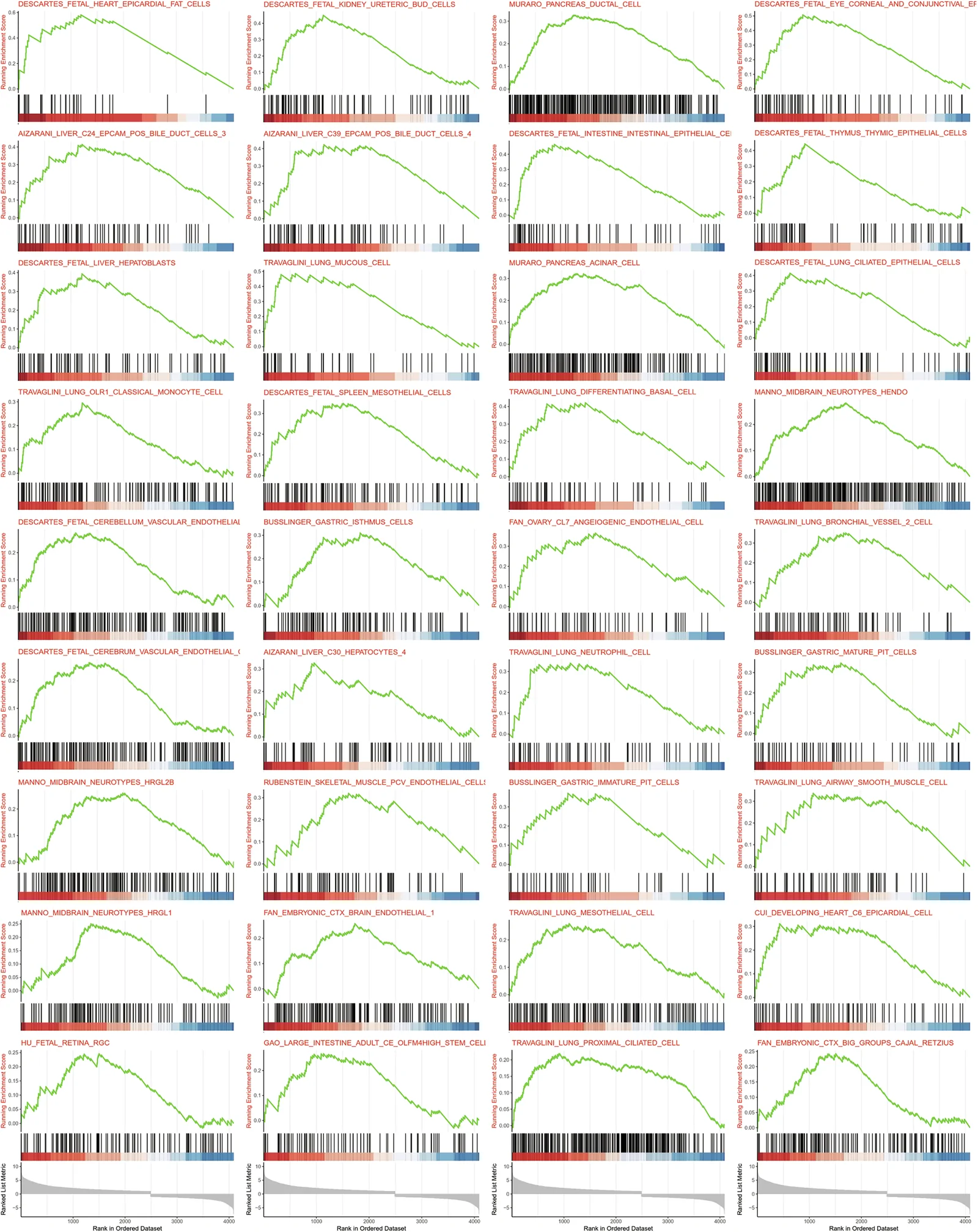
GSEA of multi‑lineage fate pathways in both CFAE + 6 and CFAE + 8 + IR1/IR2 versus the CFAE + 8 control.

To characterize the molecular events underlying XEN-like cell fate initiation induced by either A-83-01 + IR1/IR2 or E-616452 alone, we performed RNA-seq on cells harvested after 8 days of induction under five conditions: CFAE + DMSO, CFAE + 8, CFAE + 8 + IR1, CFAE + 8 + IR2, and CFAE + 6. Principal component analysis (PCA) revealed that CFAE + 8 + IR1 and CFAE + 8 + IR2 samples formed a distinct cluster significantly separated from CFAE + DMSO, CFAE + 8, and CFAE + 6 samples (Fig. 3A), indicating that IRAK4 inhibition induces a unique transcriptional profile distinct from E-616452-mediated reprogramming. Further analysis categorized all expressed genes into four distinct subclusters: Subcluster 1 comprised genes with stable expression across conditions; Subcluster 2 contained genes specifically upregulated by E-616452; Subcluster 3 represented genes activated under both E-616452 and IR1/IR2 treatments; while Subcluster 4 included genes activated in both treatments but exhibiting slightly elevated expression in IR1/IR2-treated samples (Fig. 3B,C). Notably, both E-616452- and IR1/IR2-treated samples demonstrated substantially more upregulated than downregulated genes (Fig. 3D). Gene Ontology (GO) analysis of overlapping differentially expressed genes (DEGs) between IR1/IR2 and E-616452 treatments revealed significant enrichment for multi-lineage developmental processes including epithelial tube morphogenesis, neurogenesis, gland development, renal/kidney organogenesis, digestive system development, and cell fate commitment (Figs. 3E and EV2A–C). Among the three conditions, CFAE + 8 + IR2 activated genes associated with cell fate specification and markers representative of all three germ layers, recapitulating the chemically activated multi-lineage priming (CaMP) state we previously described (Yang et al, 2022). CFAE + 6 induced endodermal markers (e.g., *Sall4* and *Sox17*), mesodermal markers (e.g., *Tbx3* and *Hand2*), and mesodermal and ectodermal co-expressed markers (e.g., *Msx2* and *Evx2*). These genes are typically co-expressed in common cells, which are also referred to as hybrid cells (Appendix Fig. S3). Whereas CFAE + 8 + IR1 only sporadically activated a few genes (e.g., *Sall4, Sox17, Tbx3, Hey2,* and *Pou3f1*) of mesodermal, ectodermal, and endodermal lineages (Fig. 3F). Gene Set Enrichment Analysis (GSEA) confirmed that overlapping genes induced by both treatments contributed to high enrichment scores for diverse progenitor lineages including fetal corneal/conjunctival epithelial cells, pancreatic ductal cells, and fetal liver hepatoblasts (Figs. 3G,H and EV3). Crucially, both IR1/IR2 and E-616452 co-activated core XEN master regulators (*Sox17, Gata4, Sall4*, and *Foxa2*) while simultaneously repressing IRAK1/4 expression (Fig. 3I). This reciprocal expression pattern between IRAK1/4 and XEN master genes further supported IRAK4’s role as a molecular barrier during MEF-to-XEN reprogramming.

### IR1 and IR2 alleviate cell cycle phase transition blockade during MEF-XEN Reprogramming

**Figure 4:**
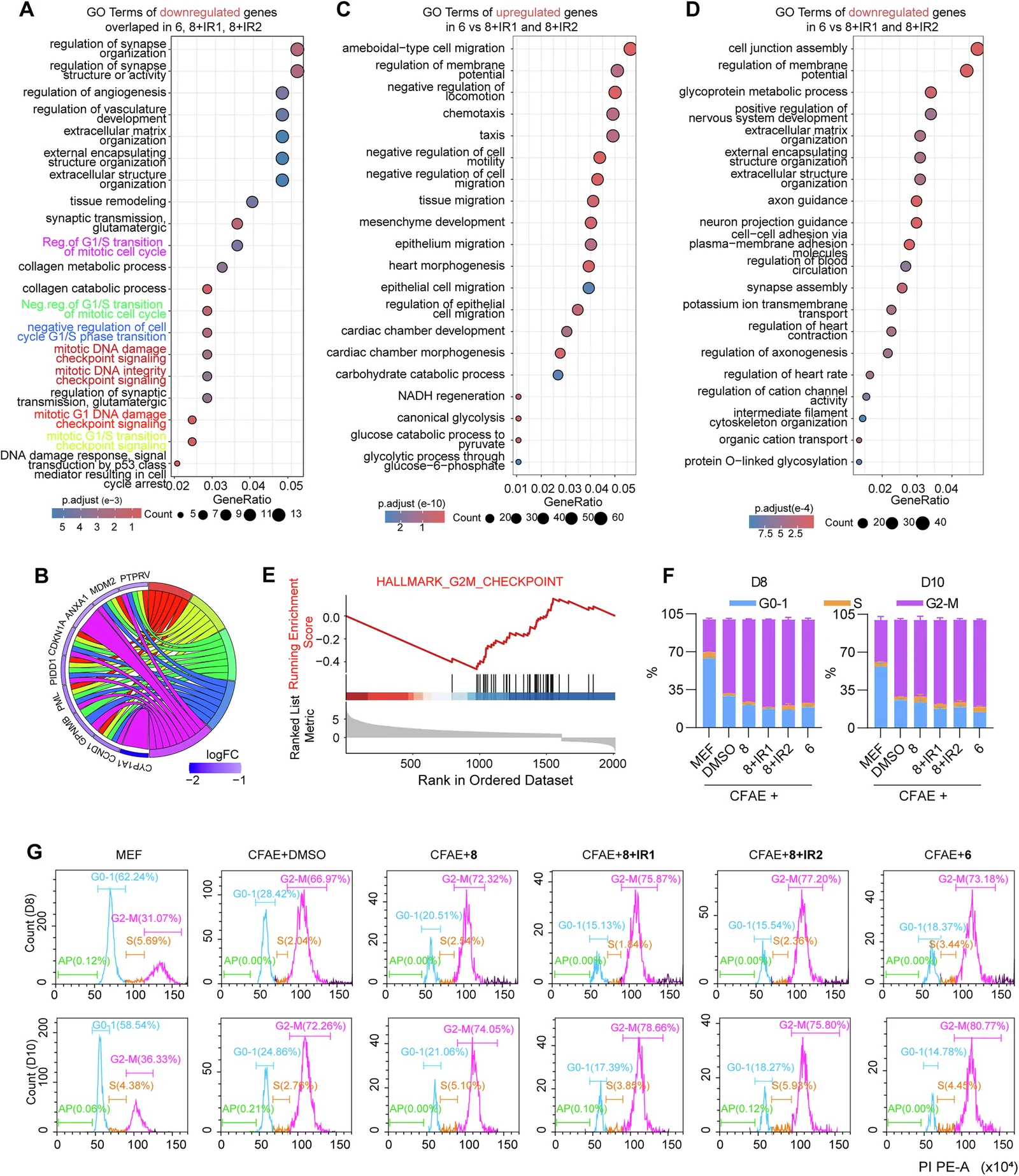
IR1 and IR2 removed the repression of cell cycle phase transition during MEF-XEN reprogramming. (**A**) Biological processes (BP) of gene ontology (GO) enrichment in downregulated genes of both CFAE + 6 and CFAE + 8 + IR1/IR2 versus CFAE + 8. Terms in colors are involved in the cell cycle. (**B**) Enrichment chord diagram of genes and cell cycle-related terms in (**A**). The *P* value was calculated using the Hypergeometric test. (**C**, **D**) Biological processes (BP) of gene ontology (GO) enrichment in upregulated (**C**) or downregulated (**D**) genes of CFAE + 6 versus CFAE + 8 + IR1/IR2. The *P* value was calculated using the Hypergeometric test. (**E**) GSEA enrichment of G2M checkpoint hallmark genes in both CFAE + 6 and CFAE + 8 + IR1/IR2. (**F**, **G)** Percentages of cell cycle phases in MEF, CFAE + DMSO, CFAE + 8, CFAE + 8 + IR1/IR2, and CFAE + 6 medium cultured cells for 8 or 10 days (*n* = 3 biological replicates), respectively. Source data are available online for this figure.

**Figure EV4:**
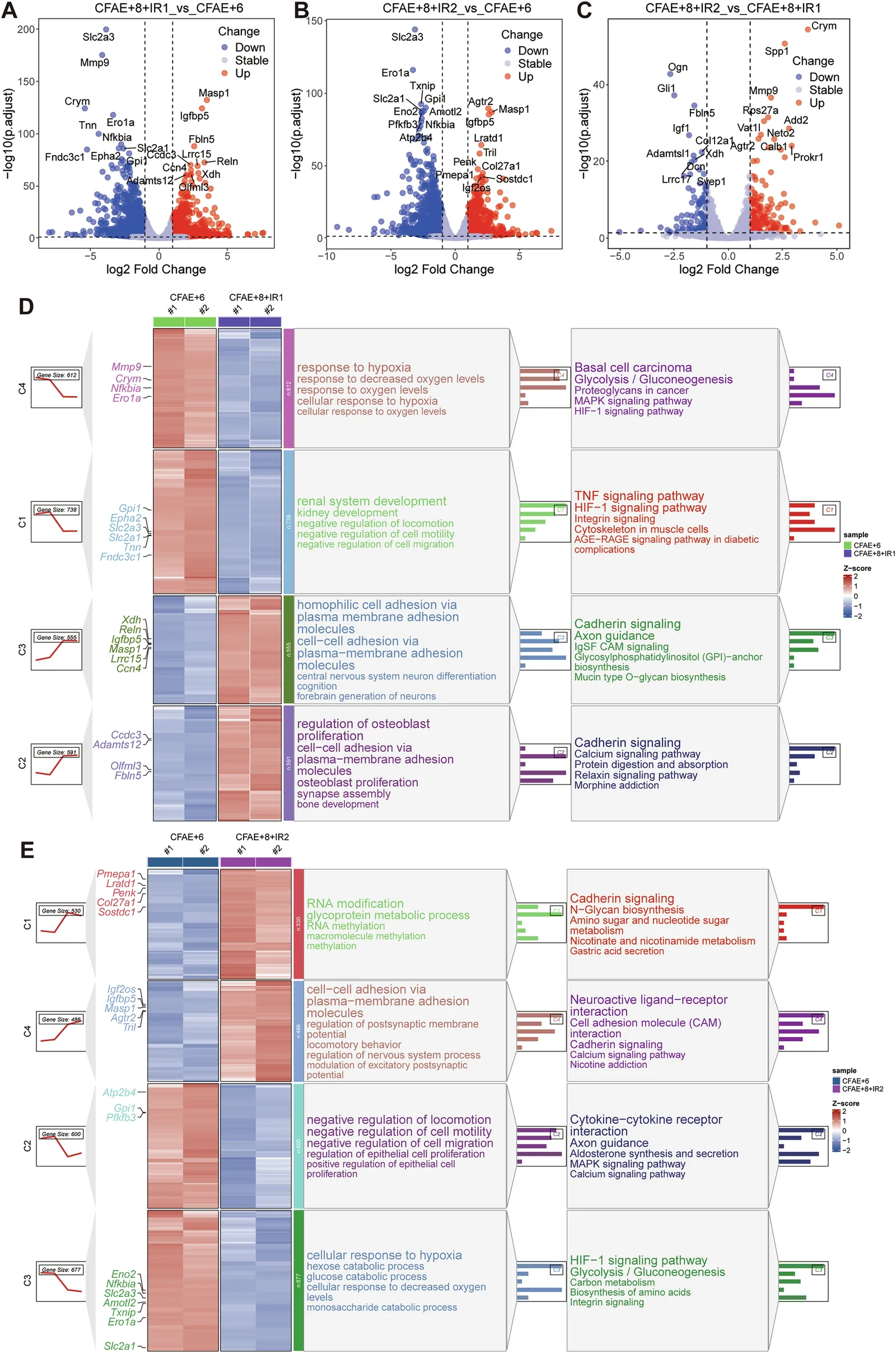
Differential gene expression and comparative transcriptomic analysis between indicated conditions. (**A**–**C**) Volcano plot of CFAE + 8 + IR1, CFAE + 8 + IR2 versus CFAE + 6, or CFAE + 8 + IR2 versus CFAE + 8 + IR1 (**A**–**C**: *n* = 2 biological replicates, respectively). The *P* value was calculated using the Wald test. Significantly up- and down-regulated genes are defined by |log2FC|> 1 and *p.*adjust <0.05. (**D**, **E**) Heatmap, GO, and KEGG enrichments of differentially expressed transcripts in CFAE + 8 + IR1 or CFAE + 8 + IR2 versus CFAE + 6. Enriched GO-BP and KEGG pathways are displayed with font size scaled to significance.

**Figure EV5:**
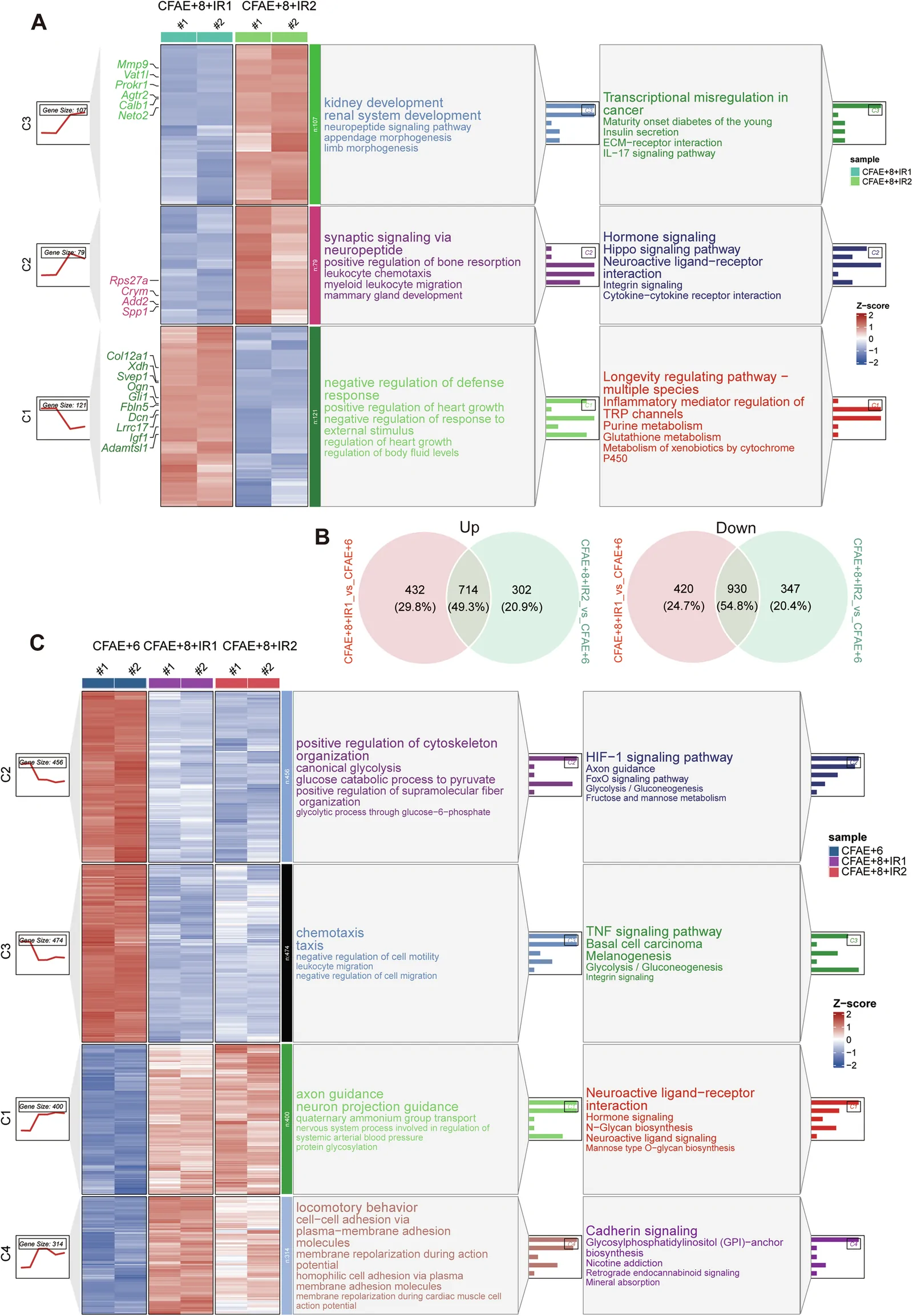
Comparative transcriptomic analysis between indicated conditions. (**A**) Heatmap, GO, and KEGG enrichments of differentially expressed transcripts in CFAE + 8 + IR2 versus CFAE + 8 + IR1. Enriched GO-BP and KEGG pathways are displayed with font size scaled to significance. (**B**) Venn diagrams show the common down- or up-regulated genes between CFAE + 8 + IR1 versus C6FAE and CFAE + 8 + IR2 versus C6FAE. (**C**) Heatmap, GO, and KEGG enrichments of transcripts in the intersection of comparisons. Enriched GO-BP terms and KEGG pathways for each cluster are displayed, with font size scaled to enrichment significance.

Further Gene Ontology (GO) analysis of downregulated genes common to both IR1/IR2-treated and E-616452-treated groups demonstrated significant enrichment in terms related to extracellular matrix organization, notably including collagen metabolic and catabolic processes, as well as critical cell cycle regulatory functions encompassing mitotic DNA damage/integrity checkpoint signaling and regulation of G1/S phase transition (Figs. 4A and EV2D–F). Key cell cycle regulators contributing to these signatures included CCND1 and CDKN1A (Fig. 4B), underscoring a shared transcriptional impact on core cell cycle machinery. These findings were corroborated by GSEA analysis results revealing significant negative enrichment of the G2/M checkpoint regulation pathway following both treatments (Fig. 4E), indicative of concerted disruption of this critical checkpoint. Contrastingly, comparative GO analysis of DEGs uniquely altered across 8-, 6-, 8 + IR1-, and 8 + IR2-treated samples indicated that E-616452 preferentially activated lineage-non-specific terms (Fig. 4C,D). Notably, no lineage-related terms were enriched in the comparisons of 6-vs-8 + IR1, 6-vs-8 + IR2, or 8 + IR1-vs-8 + IR2 (Figs. EV4 and EV5). This suggests divergent initial molecular actions despite the noted convergence on cell cycle functions. To functionally validate these transcriptional alterations impacting cell cycle progression, comprehensive flow cytometry analyses were performed on cells harvested at days 8 and 10 post-induction across six distinct conditions: MEF medium, CFAE + DMSO, CFAE + A-83-01 (CFAE + 8), CFAE + A-83-01 combined with either IR1 or IR2 (CFAE + 8 + IR1/IR2), and CFAE + E-616452 (CFAE + 6). Relative to MEF medium control, treatment with CFAE + DMSO alone by day 8 elicited a substantial cell cycle redistribution, significantly reducing the G0/G1 population by approximately 34.8% (*p* = 6.7e-6) while concurrently increasing the G2/M fraction by approximately 38.2% (*p* = 9.9e-7). This baseline shift was modestly but significantly amplified by the addition of A-83-01 (CFAE + 8 versus CFAE + DMSO: ΔG0/G1 = −8.1%, p = 2.45e-5; ΔG2/M = +7.7%, *p* = 3.44e-4). Both CFAE + 8 + IR1/IR2 and CFAE + 6 treatments induced statistically significant further reductions in G0/G1 and increases in G2/M phases relative to CFAE + 8 at day 8 (CFAE + 8 + IR1 vs CFAE + 8: ΔG0/G1 = -3.7%, *p* = 6.72e-3; ΔG2/M = +4.7%, *p* = 3.12e-4; CFAE + 8 + IR2 vs CFAE + 8: ΔG0/G1 = −4.59%, *p* = 1.11e-3; ΔG2/M = +3.22%, *p* = 0.039). By day 10, the initial redistribution induced by CFAE + DMSO persisted robustly (ΔG0-1: ~−30.9%, *p* = 8.3e-4; ΔG2/M: ~+32.13%, *p* = 5.7e-4 vs. MEF medium control), while the potentiating effect of A-83-01 alone (CFAE + 8) was no longer detectable compared to CFAE + DMSO (ΔG0/G1 = -2.44%, *p* = 0.087; ΔG2/M = +0.43%, *p* = 0.41). Strikingly, IRAK4 inhibition (CFAE + 8 + IR1/IR2) independently drove profound cell cycle remodeling relative to CFAE + 8 controls, significantly reducing G0/G1 populations (Δ = −5.68% for IR1, *p* = 0.013; Δ = −4.11% for IR2, *p* = 0.03) and significantly elevating G2/M fractions (Δ = +7.1% for IR1, *p* = 0.02; Δ = +5.2% for IR2, *p* = 0.047) (Fig. 4F,G). Collectively, these integrated transcriptional and functional analyses demonstrate that despite acting on distinct primary molecular targets, both E-616452 and IRAK4 inhibitors converge functionally to alter cell cycle dynamics. These changes are associated with accelerated reprogramming kinetics, suggesting that cell cycle remodeling may be an indispensable factor.

### Inhibition of IRAK4 enhances chromatin accessibility and promotes cell fate transition

**Figure 5:**
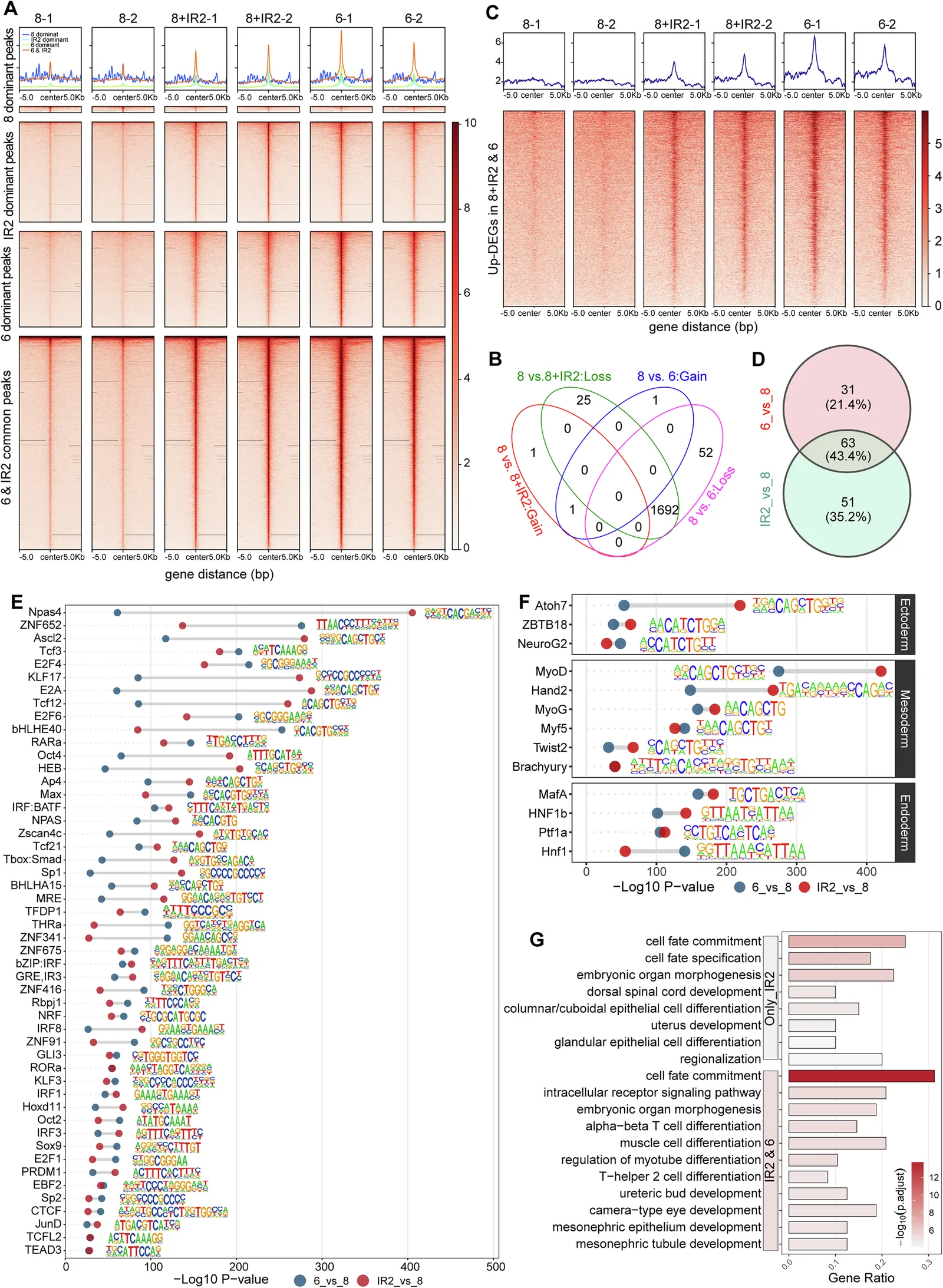
IRAK4 inhibition enhances chromatin accessibility and promotes cell fate transition. (**A**) ATAC-seq analysis of chromatin accessibility near TSS in MEF medium, CFAE + 6, CFAE + 8, and CFAE + 8 + IR2 cultured cells for 8 days, respectively. −1 and −2 represent two replicates. (**B**) Venn diagram of peaks (loss and gain) from the union of the 8 vs. 6 and 8 vs. 8 + IR2 comparisons. (**C**) PlotHeatmap of ATAC-seq data for upregulated genes overlapped in 8 + IR2 and 6. (**D**) Venn diagram of transcription factors enriched in ATAC-seq data of 6_vs_8 and 8 + IR2_vs_8. (**E**, **F**) Enriched significant transcription factors: DNA motif pairs in the intersection of 6_vs_8 and 8 + IR2_vs_8. Three germ-layer-related transcription factors: DNA motif pairs was showed in (**F**). The *P* value was calculated using the Hypergeometric test. (**G**) 63 TFs in (**D**) enriched GO terms. The *P* value was calculated using the cumulative hypergeometric distribution test.

To gain deeper insights into the role of IRAK4 in regulating the early stages of XEN-like cell induction, we next investigated changes in chromatin accessibility. MEFs cultured in CFAE + 8, CFAE + 8 + IR2, and CFAE + 6 media were harvested at day 8 of induction for ATAC-seq (assay for transposase-accessible chromatin using sequencing). Consistent with our RNA-seq results, which showed that the number of upregulated genes substantially exceeded that of downregulated genes under induction by 8 + IR2 and 6 (Fig. 5A), ATAC-seq analyses revealed that both CFAE + 8 + IR2 and CFAE + 6 conditions promoted a transition of chromatin accessibility from a relatively “closed” to an “open” state. Following peak calling of the ATAC-seq data, we identified 1692 commonly gained peaks in both IR2 and E-616452 conditions (Fig. 5B). Analysis of the union of upregulated genes in CFAE + 8 + IR2 and CFAE + E-616452 demonstrated that IR2 and E-616452 induced more “open” chromatin states, suggesting that alterations in chromatin accessibility contribute to gene activation under these conditions (Fig. 5C). Further motif analysis of the peaks revealed that 63 motifs (43.4%) were shared between IR2_vs_8 and 6_vs_8 comparisons, whereas 31 (21.4%) and 51 (35.2%) motifs were predominantly enriched in 6_vs_8 and IR2_vs_8, respectively (Fig. 5D). Upon identifying TF-DNA motif interactions, we found that the 63 common motifs in 6_vs_8 and IR2_vs_8 could be bound by multi-lineage or pluripotency-associated transcription factors, including IRF:BATF, TCF21, IRF8, NeuroG2, MyoD, Twist2, HNF1b, and Oct4 (Fig. 5E,F), further suggesting that inhibition of IRAK4 activates a cell fate transition toward a CaMP state. Gene Ontology (GO) analysis indicated that these binding transcription factors were enriched for cell fate-related terms, such as cell fate commitment and embryonic organ morphogenesis (Fig. 5G). Collectively, these results show that IRAK4 inhibition is associated with increased chromatin accessibility at lineage development-associated genes, raising the possibility that this chromatin remodeling contributes to the enhanced reprogramming.

### Inhibition of IRAK4 facilitates the chemical induction of XEN-like colonies

**Figure 6:**
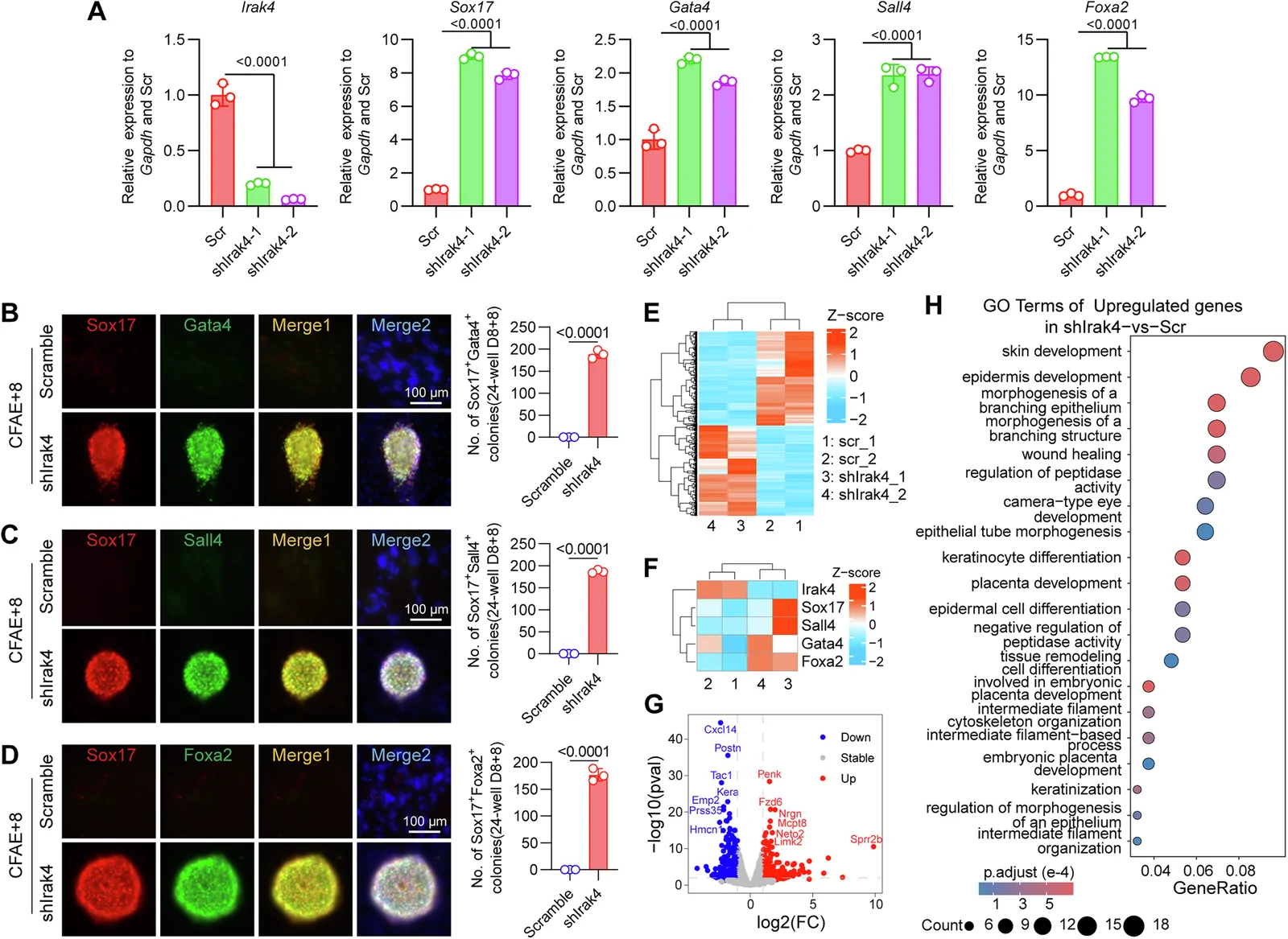
IRAK4 Knockdown induced XEN-like colonies. (**A**) Quantitative PCR for *Sox17*, *Gata4*, *Sall4*, and *Foxa2* of scramble, shIrak4-1 and shIrak4-2 in CFAE + 8 for 12 days, respectively. Data were represented as mean ± SD. *P* values were calculated using one-way ANOVA followed by Dunnett multiple comparisons test (*n* = 3 biological replicates, respectively). *Irak4*: Scr vs. shlrak4-1: *p* = 4.6e-6; Scr vs. shlrak4-2: *p* = 1.8e-6; *Sox17:* Scr vs. shlrak4-1: *p* = 3.5e-9; Scr vs. shlrak4-2: *p* = 8.7e-9; *Gata4*: Scr vs. shlrak4-1: *p* = 7.1e-6; Scr vs. shlrak4-2: *p* = 4.8e-5; *Sall4*: Scr vs. shlrak4-1: *p* = 3.2e-5; Scr vs. shlrak4-2: *p* = 2.9e-5; *Foxa2*: Scr vs. shlrak4-1: *p* = 1.0e-9; Scr vs. shlrak4-2: *p* = 8.8e-9. (**B**–**D**) Immunofluorescence co-staining of Sox17 and Gata4 (**B**), Sall4 (**C**), or Foxa2 (**D**) in *scramble* and *shIrak4* under CFAE + 8 induction for 16 days, respectively. Left panel, immunofluorescence co-staining. Scale bar: 500 μm. Right panel, statistical analysis of the number of Sox17^+^Gata4^+^, Sox17^+^Sall4^+^, and Sox17^+^Foxa2^+^ colonies. Data were represented as mean ± SD. Numbers in each panel are *P* values between the control and indicated samples (*n* = 3 biological replicates, respectively). *p* = 4.3e-6, 9.2e-8, 1.2e-5 in (**B**–**D**) respectively. *P* values were calculated using two-tailed unpaired Student’s *t*-tests. (**E**) Heatmap of differentially expressed genes in RNA-seq data between *scramble* and *shIrak4* in CFAE + 8. (**F**) Heatmap of *Irak4, Sox17, Gata4, Sall4*, and *Foxa2* expression levels from *shIrak4* versus *scramble* RNA-seq data in CFAE + 8. (**G**) Differentially expressed gene analysis of RNA-seq data from *shIrak4* versus *scramble* with DESeq2. The *P* value was calculated using the Wald test. Cutoff: −log10(*p*val) >2 and |log2(fold change)|>1. Blue represents downregulated genes. Red represents upregulated genes. Gray represents unchanged genes. (**H**) Biological processes (BP) of gene ontology (GO) enrichment in upregulated genes of *shIrak4* versus *scramble*. The *P* value was calculated using the Hypergeometric test. Source data are available online for this figure.

Building upon small molecular evidence that IRAK4 inhibitors IR1/IR2 promote reprogramming, we next employed genetic approaches to definitively establish IRAK4’s functional role. Stable knockdown of *Irak4* (*shIrak4*) in mouse embryonic fibroblasts (MEFs) robustly activated core XEN master transcription factors (*Sox17, Gata4, Sall4, Foxa2*) and significantly enhanced formation of XEN-like colonies (Fig. 6A–D). Global transcriptomic investigation revealed substantial transcriptional changes following *Irak4 knockdown*, including the upregulation of numerous genes—notably the XEN master regulators themselves. GO analysis demonstrated significant enrichment among these upregulated genes for terms associated with lineage-specific development, morphogenesis, and differentiation, including skin development, camera-type eye development, placental development, epidermal cell differentiation, and keratinocyte differentiation (Fig. 6E–H). GSEA analysis further corroborated these findings, indicating that *Irak4* knockdown promotes transcriptional programs characteristic of diverse progenitor cell types, including fetal liver hepatoblasts, kidney ureteric bud cells, and pancreatic ductal cells. Conversely, GSEA analysis highlighted significant suppression of neural lineage programs, such as those associated with midbrain neurotypes and bone marrow stromal cells (Appendix Fig. S4). Collectively, these genetic loss-of-function experiments demonstrate that Irak4 suppression potently licenses acquisition of a multi-lineage-primed state capable of adopting extraembryonic endodermal fates, providing mechanistic validation that IRAK4 functions as a bona fide barrier constraining cellular plasticity during MEF-to-XEN reprogramming. The concordance between genetic and pharmacological suppression of IRAK4—both yielding indistinguishable multi-lineage priming phenotypes—further substantiates target specificity and positions IRAK4 kinase activity as a critical gatekeeper of somatic cell identity.

### IRAK4 overexpression repressed MEF-XEN reprogramming

**Figure 7:**
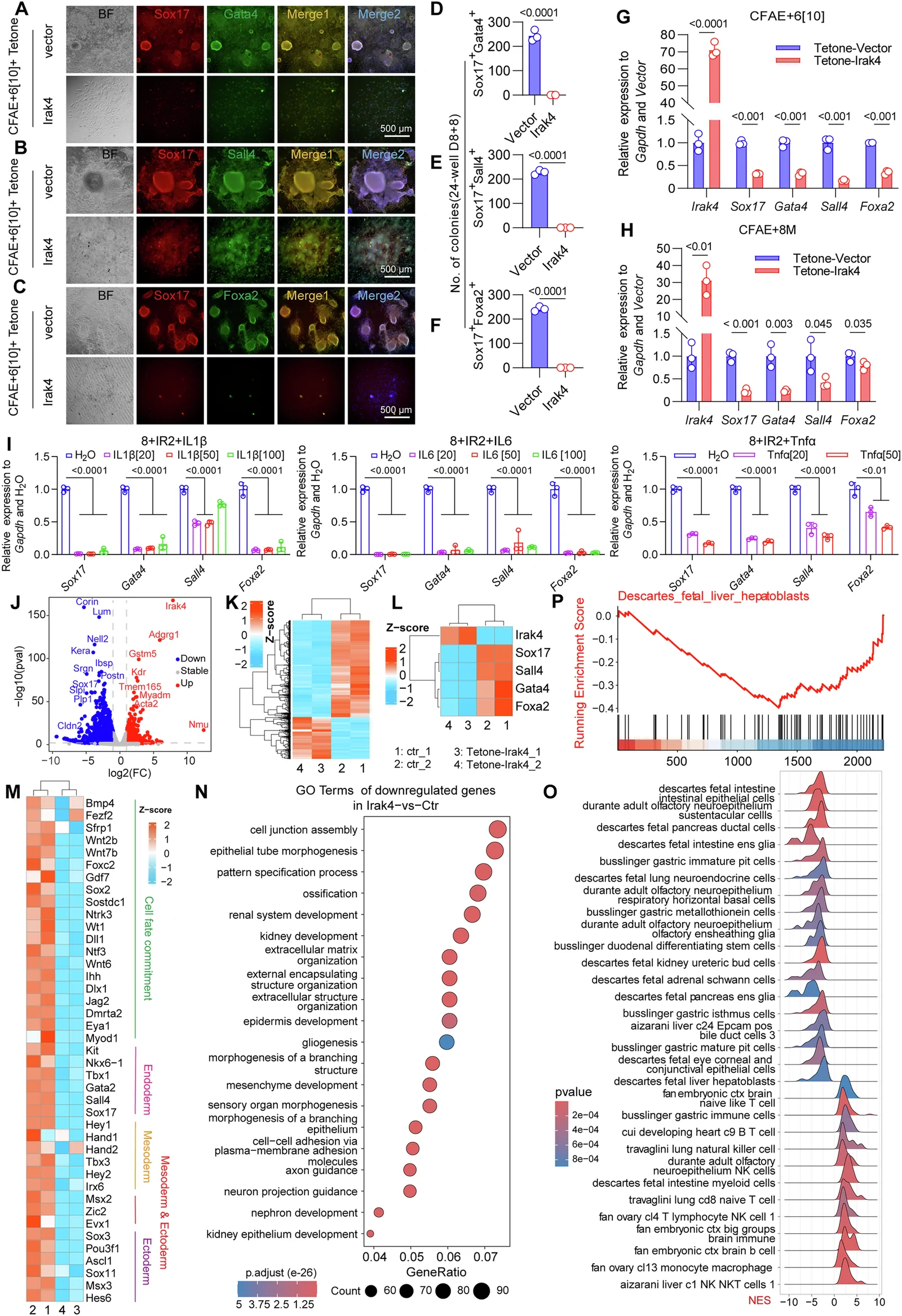
IRAK4 overexpression repressed MEF-XEN reprogramming. (**A**–**C**) Immunofluorescence co-staining of Sox17 and Gata4 (**A**), Sall4 (**B**), or Foxa2 (**C**) in *vector* and *tetone-Irak4* under CFAE + 6 induction for 16 days, respectively. Scale bar: 500 μm. (**D**–**F**) The number of Sox17^+^Gata4^+^, Sox17^+^Sall4^+^, and Sox17^+^Foxa2^+^ colonies in (**A**). Data were represented as mean ± SD. *p* = 4.4e-5, 2.0e-6, 1.6e-6 in (**D**–**F**) respectively. (**G**, **H**) Quantitative PCR of *Irak4*, *Sox17, Gata4*, *Sall4*, and *Foxa2* in *vector* and *tetone-Irak4* under CFAE + 6 (**G**) or CFAE + 8 M (**H**) induction for 16 days, respectively. M, MSC2530818. Data were represented as mean ± SD. In (**G**) Irak4: *p* = 4.73e-6; Sox17: *p* = 5.63e-4; Gata4: *p* = 5.92e-4; Sall4: *p* = 2.31e-4; Foxa2: *p* = 7.02e-4. In (**H**) Irak4: *p* = 1.96e-3; Sox17: *p* = 9.55e-4; Gata4: *p* = 3.26e-3; Sall4: *p* = 0.045; Foxa2: *p* = 0.035. For (**D**–**H**), Numbers in each panel are *P* values between DMSO and indicated samples (*n* = 3 biological replicates, respectively). *P* values were calculated using two-tailed unpaired Student’s *t*-tests. (**I**) mRNA levels of XEN master regulators in CFAE + 8 + IR2 + IL1β, IL6, and TNFα. Numbers in brackets are the concentrations (ng/mL) of cytokines. Data were presented as mean ± SD. *P* values were calculated using one-way ANOVA followed by Dunnett multiple comparisons test (*n* = 3 biological replicates, respectively). Left panel: *Sox17* (H₂O vs. IL1β[20]: *p* = 3.0e-10; H₂O vs. IL1β[50]: *p* = 3.0e-10; H₂O vs. IL1β[100]: *p* = 4.2e-10), *GATA4* (H₂O vs. IL1β[20]: *p* = 6.9e-8; H₂O vs. IL1β[50]: *p* = 7.8e-8; H₂O vs. IL1β[100]: *p* = 1.4e-7), *Sall4* (H₂O vs. IL1β[20]: *p* = 5.9e-7; H₂O vs. IL1β[50]: *p* = 5.9e-7; H₂O vs. IL1β[100]: *p* = 2.6e-4), *Foxa2* (H₂O vs. IL1β[20]: *p* = 2.1e-7; H₂O vs. IL1β[50]: *p* = 2.1e-7; H₂O vs. IL1β[100]: *p* = 3.2e-7). Middle panel: *Sox17* (H₂O vs. IL6[20]: *p* = 3.2e-11; H₂O vs. IL6[50]: *p* = 3.3e-11; H₂O vs. IL6[100]: *p* = 3.3e-11), *Gata4* (H₂O vs. IL6[20]: *p* = 4.0e-9; H₂O vs. IL6[50]: *p* = 5.5e-9; H₂O vs. IL6[100]: *p* = 4.9e-9), *Sall4* (H₂O vs. IL6[20]: *p* = 2.6e-6; H₂O vs. IL6[50]: *p* = 7.5e-6; H₂O vs. IL6[100]: *p* = 3.9e-6), *Foxa2* (H₂O vs. IL6[20]: *p* = 3.3e-8; H₂O vs. IL6[50]: *p* = 3.4e-8; H₂O vs. IL6[100]: *p* = 3.3e-8). Right panel: *Sox17* (H₂O vs. Tnfa[20]: *p* = 1.8e-7; H₂O vs. Tnfa[50]: *p* = 5.9e-8), *Gata4* (H₂O vs. Tnfa[20]: *p* = 1.3e-7; H₂O vs. Tnfa[50]: *p* = 8.9e-8), *Sall4* (H₂O vs. Tnfa[20]: *p* = 4.2e-5; H₂O vs. Tnfa[50]: *p* = 1.3e-5), *Foxa2* (H₂O vs. Tnfa[20]: *p* = 0.0017; H₂O vs. Tnfa[50]: *p* = 1.0e-4). (**J**) Volcano plot of differentially expressed gene analysis of RNA-seq data (*n* = 2 biological replicates, respectively) from *tetone-Irak4* versus *vector* under CFAE + 6 induction for 16 days with DESeq2. The *P* value was calculated using the Wald test. Cutoff: −log10(*p*val) >2 and |log2(fold change)|>1. Blue represents downregulated genes. Red represents upregulated genes. Gray represents unchanged genes. (**K**) Heatmap of differentially expressed genes in *tetone-Irak4* versus *vector*. (**L**) Heatmap of *Irak4, Sox17, Gata4, Sall4*, and *Foxa2* expression levels in *tetone-Irak4* versus *vector*. (**M**) Heatmap of cell fate commitment and three germ layers relative genes in *tetone-Irak4* and *vector*. (**N**) Biological processes (BP) of gene ontology (GO) enrichment in downregulated genes of *tetone-Irak4* versus *vector*. The *P* value was calculated using the hypergeometric test. (**O**) GSEA enrichment distributions of key differentially expressed genes in *tetone-Irak4* versus *vector*. The *P* value was calculated using the permutation test. (**P**) GSEA enrichment of fetal liver hepatoblasts fate in *tetone-Irak4* versus *vector*. Source data are available online for this figure.

To definitively establish IRAK4 as a molecular barrier to cellular reprogramming, we overexpressed Irak4 in mouse embryonic fibroblasts (MEFs) exposed to two high-efficiency small molecule cocktails known to induce XEN cell fate: CFAE + 6 and CFAE + A-83-01 + MSC2530818 (CFAE + 8 M). Strikingly, IRAK4 overexpression completely abrogated the formation of XEN-like colonies and significantly suppressed the expression of key XEN master transcription factors, including *Sox17, Gata4, Sall4*, and *Foxa2* (Fig. 7A–H). To mimic IRAK4 overexpression, we next treated cells with IL1β, IL6, and TNFα in CFAE + 8 + IR2 medium to test their effect on cell reprogramming, respectively. The results showed that these cytokines significantly downregulated the expression of XEN marker genes and blocked XEN formation (Fig. 7I). Global transcriptomic analysis revealed that IRAK4 overexpression induced a profound shift in gene expression, with significantly more downregulated than upregulated genes. This suppressed gene set encompassed numerous critical transcription factors and developmental regulators associated with mesodermal, endodermal, and ectodermal lineages (Fig. 7J–M). GO analysis of downregulated genes demonstrated significant enrichment in essential developmental processes, including kidney development, epidermis development, mesenchyme development, epithelial tube morphogenesis, and ossification. Concordantly, GSEA indicated that IRAK4 overexpression caused significant negative enrichment of diverse progenitor cell types, such as fetal intestinal cells, fetal pancreatic ductal cells, and gastric immature pit cells. Conversely, it resulted in robust positive enrichment of gene programs linked to immune cell development and function (Fig. 7N,O; Appendix Fig. S5). This pronounced pro-immunogenic shift in the transcriptional landscape is consistent with our prior observation that CDK8 inhibitors facilitate reprogramming by suppressing inflammatory signaling pathways (Li et al, 2023). However, whether the immune-related gene activation upon IRAK4 overexpression plays a causal role in blocking reprogramming or merely represents a secondary consequence of enforced IRAK4 expression remains to be determined. Critically, the profound inhibitory effect of IRAK4 overexpression on cellular plasticity directly contrasts with the phenotype elicited by its genetic knockdown or pharmacological inhibition. Specifically, the fetal liver hepatoblast transcriptional signature, which was strongly promoted by IRAK4 inhibition (Fig. 3H), was significantly suppressed upon Irak4 overexpression (Fig. 7P). Collectively, these complementary loss- and gain-of-function experiments demonstrate that IRAK4 suppression potently enhances MEF-to-XEN reprogramming efficiency. Conversely, enforced IRAK4 expression imposes a dominant barrier to cellular plasticity by globally repressing a core network of developmental transcription factors across germ layers while diverting cell fate trajectories towards immunogenic programs, firmly establishing its role as a master regulator restricting cellular identity transitions.

### IRAK4 inhibitors enhance functional reprogramming to Neuron-like cells

**Figure 8:**
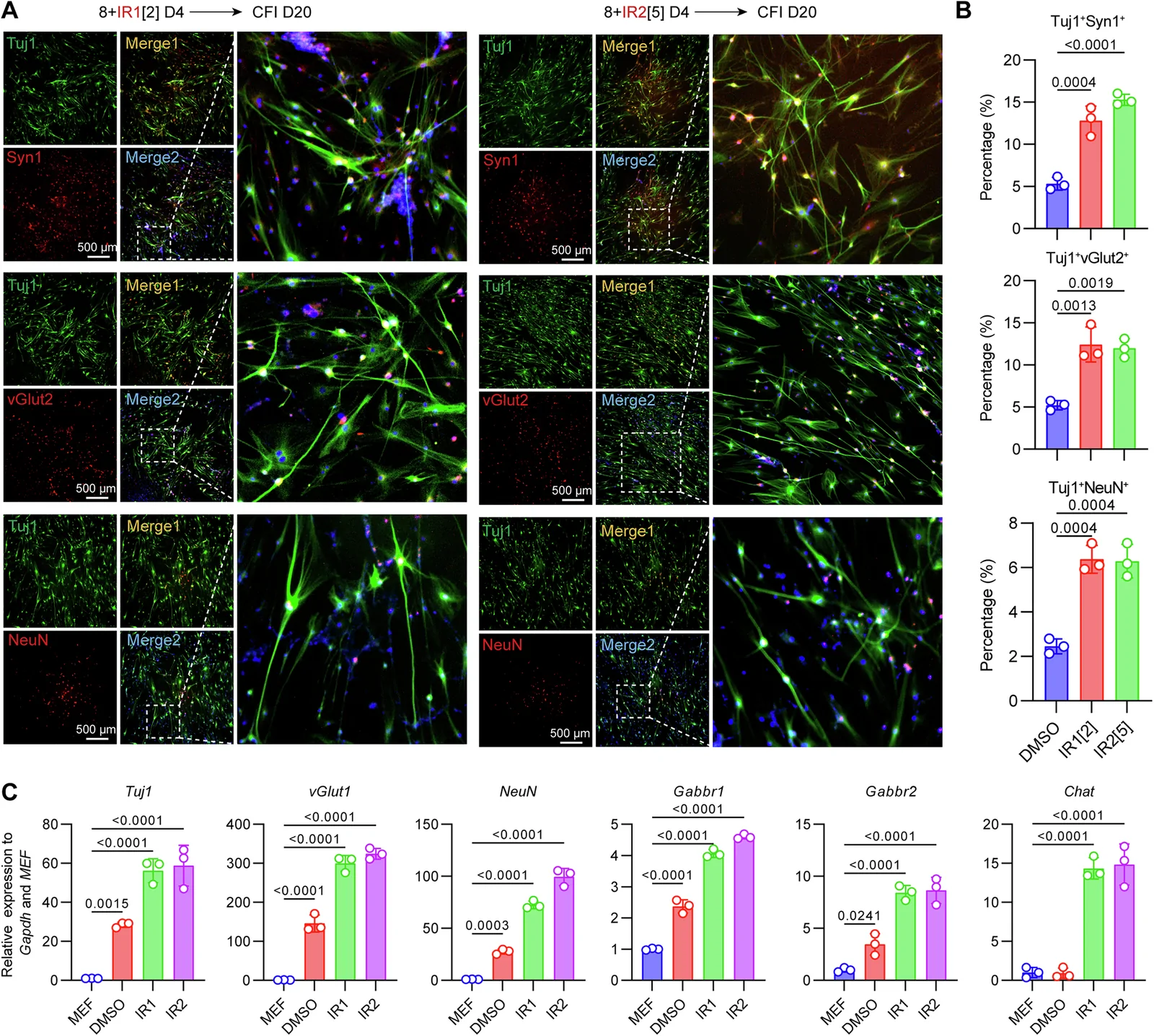
IRAK4 inhibitors enhance functional reprogramming to Neuron-like cells. (**A**) Immunostaining of chemically induced neuron-like cells induced by the indicated cocktails. Scale bar, 500 μm. CFI: CHIR99021, Forskolin, ISX-9. (**B**) Percentages of Tuj1^+^Syn1^+^ (DMSO vs. IR1[2]: *p* = 0.0004; DMSO vs. IR2[5]: *p* = 7.6e-5), Tuj1^+^vGlut2^+^ ((DMSO vs. IR1[2]: *p* = 0.0013; DMSO vs. IR2[5]: *p* = 0.0019) and Tuj1^+^NeuN^+^ neuron-like cells (DMSO vs. IR1[2]: *p* = 0.0004; DMSO vs. IR2[5]: *p* = 0.0004). Data were represented as mean ± SD. *P* values were calculated using one-way ANOVA followed by Dunnett multiple comparisons test (*n* = 3 biological replicates, respectively). (**C**) Relative mRNA expression of typical neuronal marker genes in neuron-like cells. Data were represented as mean ± SD. *P* values were calculated using one-way ANOVA followed by Dunnett multiple comparisons test (*n* = 3 biological replicates, respectively). *Tuj1*(MEF vs. DMSO: *p* = 0.0015; MEF vs. IR1: *p* = 1.0e-5; MEF vs. IR2: *p* = 7.4e-6), *vGlut1*(MEF vs. DMSO: *p* = 1.3e-5; MEF vs. IR1: *p* = 4.6e-8; MEF vs. IR2: *p* = 2.5e-8), *NeuN*(MEF vs. DMSO: *p* = 0.0003; MEF vs. IR1: *p* = 1.7e-7; MEF vs. IR2: *p* = 1.4e-8), *Gabbr1*(MEF vs. DMSO: *p* = 3.3e-6; MEF vs. IR1: *p* = 5.9e-9; MEF vs. IR2: *p* = 1.6e-9), *Gabbr2*(MEF vs. DMSO: *p* = 0.0241; MEF vs. IR1: *p* = 1.9e-5; MEF vs. IR2: *p* = 1.5e-5), *Chat*(MEF vs. DMSO: *p* = 0.9998; MEF vs. IR1: *p* = 1.5e-5; MEF vs. IR2: *p* = 1.2e-5). Source data are available online for this figure.

To validate that CaMP state cells induced during the MEF-to-XEN transition can ultimately be reprogrammed into functional cells, we selected the neural lineage as the target cell type. For neural cell fate reprogramming, cells were initially treated in CFAE + 8 + IR1/2 medium for 4 days. Following this transitory phase, the cells were transferred to neural lineage induction medium (CFI) for an additional 16 days. Upon completion of induction, immunofluorescence and qPCR analyses revealed that neural lineage marker genes, including Syn1, vGlut2, and NeuN, exhibited varying degrees of co-expression with Tuj1 in cells displaying neuronal-like morphologies (Fig. 8A,B). Furthermore, additional neural cell markers, including *vGlut1*, *Gabbr1/2*, and *Chat* mRNAs, were also significantly upregulated by IRAK4 inhibitors IR1/IR2 (Fig. 8C). These results collectively demonstrate that MEFs exposed to IRAK4 inhibitors exhibit an enhanced propensity toward neural cell fate conversion.

### IRAK4 inhibitors enhance functional reprogramming to hepatocyte-like cells

**Figure 9:**
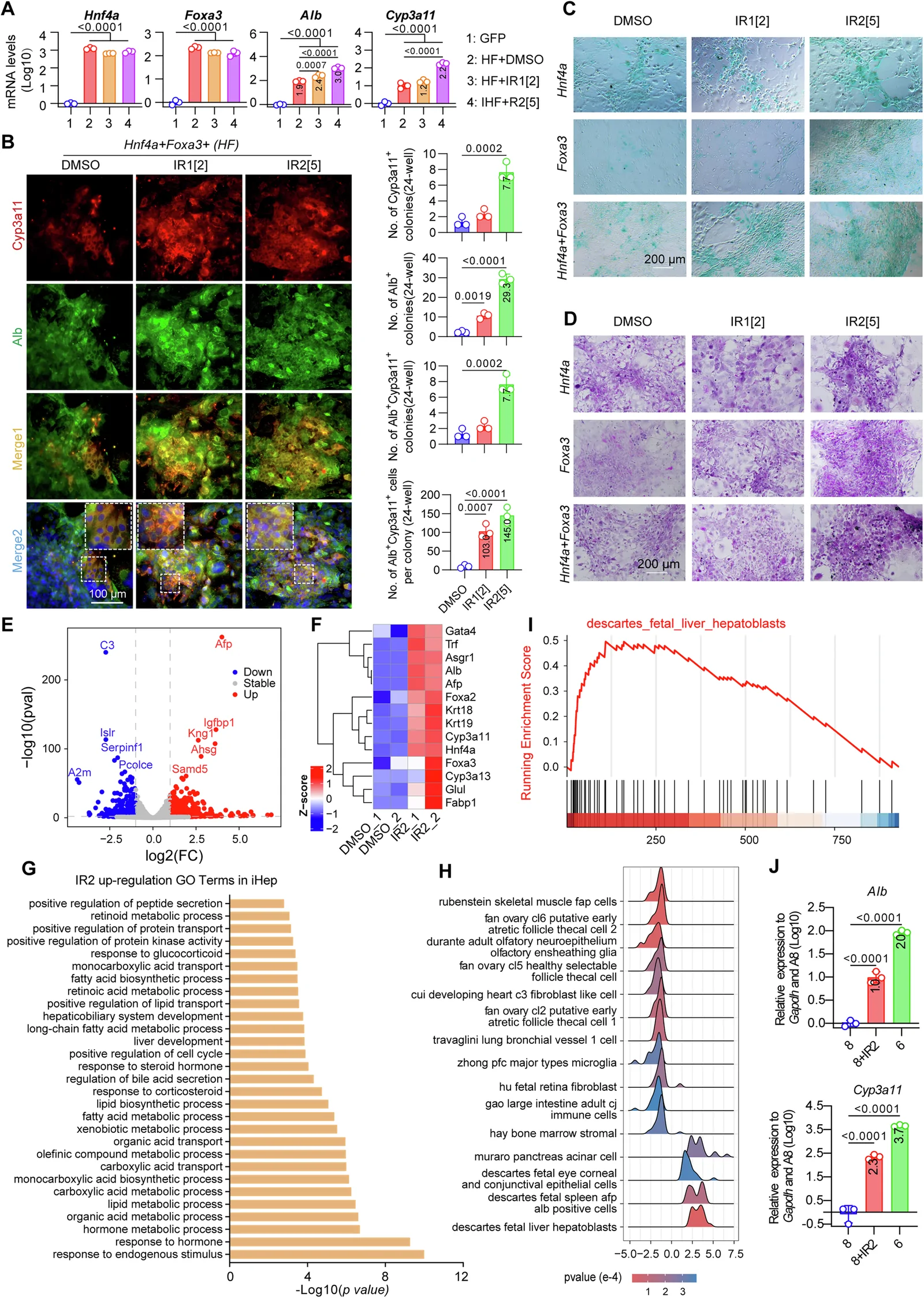
IRAK4 inhibitors enhance mouse hepatocyte reprogramming. (**A**) Quantitative PCR of *Hnf4a, Foxa3, Alb, and Cyp3a11* mRNA. Data were represented as mean ± SD. *P* values were calculated using one-way ANOVA followed by Tukey’s multiple comparisons test (*n* = 3 biological replicates, respectively). *Hnf4a*(GFP vs. HF-DMSO: *p* = 5.3e-14; GFP vs. HF-IR1[2]: *p* = 1.0e-13; GFP vs. HF-IR2[5]: *p* = 7.0e-14), *Foxa3*(GFP vs. HF-DMSO: *p* = 5.3e-9; GFP vs. HF-IR1[2]: *p* = 9.3e-9; GFP vs. HF-IR2[5]: *p* = 1.0e-8), *Alb*(GFP vs. HF-DMSO: *p* = 1.2e-8; GFP vs. HF-IR1[2]: *p* = 3.6e-9; GFP vs. HF-IR2[5]: *p* = 3.8e-10; HF-DMSO vs. HF-IR1[2]: *p* = 0.0007; HF-DMSO vs. HF-IR2[5]: *p* = 8.4e-7; HF-IR1[2] vs. HF-IR2[5]: *p* = 4.3e-5), *Cyp3a11*(GFP vs. HF-DMSO: *p* = 2.1e-5; GFP vs. HF-IR1[2]: *p* = 3.9e-6; GFP vs. HF-IR2[5]: *p* = 3.1e-8; HF-DMSO vs. HF-IR1[2]: *p* = 0.096; HF-DMSO vs. HF-IR2[5]: *p* = 4.1e-6; HF-IR1[2] vs. HF-IR2[5]: *p* = 2.2e-5). (**B**) Immunofluorescence co-staining of Alb and Cyp3a11 in *Hnf4a+*Foxa3-mediated hepatocyte reprogramming under HCM + C8B (C, CHIR99021, 8, A-38-01, B, and BMP4) plus DMSO, IR1, or IR2. Left panel, immunofluorescence co-staining. Scale bar: 100 μm. Right panel, statistical analysis of the number of positive clones or cell numbers. Data were represented as mean ± SD. Numbers in each panel are *P* values between DMSO and indicated samples (*n* = 3 biological replicates, respectively). *P* values were calculated using one-way ANOVA followed by Dunnett multiple comparisons test. Cyp3a11^+^ (DMSO vs. IR1[2]: *p* = 0.2983; DMSO vs. IR2[5]: *p* = 0.0002), Alb^+^(DMSO vs. IR1[2]: *p* = 0.0019; DMSO vs. IR2[5]: *p* = 2.4e-6), Alb^+^Cyp3a11^+^(DMSO vs. IR1[2]: *p* = 0.2983; DMSO vs. IR2[5]: *p* = 0.0002), Alb^+^Cyp3a11^+^ per colony (DMSO vs. IR1[2]: *p* = 0.0007; DMSO vs. IR2[5]: *p* = 8.9e-5). (**C**, **D**) Indocyanine green (ICG) (**C**) or Periodic acid-schiff (PAS) (**D**) staining of *Hnf4a, Foxa3, Hnf4a+*Foxa3-mediated hepatocyte reprogramming under HCM + C8B (C, CHIR99021, 8, A-38-01, B, BMP4) plus DMSO, IR1, or IR2. Scale bar: 200 μm. (**E**) Differentially expressed gene analysis of RNA-seq data in *Hnf4a+*Foxa3-mediated iHeps from *IR2[5]* versus *DMSO* under HCM induction for 24 days with DESeq2. The *P* value was calculated using the Wald test. Cutoff: −log10(*p*val) >2 and |log2(fold change)|>1. Blue represents downregulated genes. Red represents upregulated genes. Gray represents unchanged genes. (**F**) Heatmap of hepatocyte relative genes in *IR2[5]* versus *DMSO*. (**G**) Biological processes (BP) of gene ontology (GO) enrichment in upregulated genes of *IR2[5]* versus *DMSO*. The *P* value was calculated using the hypergeometric test. (**H**) GSEA enrichment distributions in *IR2[5]* versus *DMSO*. The *P* value was calculated using the Permutation test. (**I**) GSEA enrichment of fetal liver hepatoblasts fate in *IR2[5]* versus *DMSO*. (**J**) Quantitative PCR of *Alb and Cyp3a11* mRNA in chemical-induced hepatocyte reprogramming. Data were represented as mean ± SD. Numbers in each panel are *P* values between DMSO and indicated samples (*n* = 3 biological replicates, respectively). *P* values were calculated using one-way ANOVA followed by Dunnett multiple comparisons test. *Alb* (8 vs. 8 + IR2: *p* = 1.2e-5; 8 vs. 6: *p* = 2.1e-7), *Cyp3a11*(8 vs. 8 + IR2: *p* = 1.9e-5; 8 vs. 6: *p* = 1.5e-6). Source data are available online for this figure.

Based on our results that IRAK4 inhibition activates fetal liver hepatoblast signatures while its overexpression represses this identity (Figs. 3H and 7P), we hypothesized that IRAK4 functions as a conserved barrier in hepatocyte reprogramming. Leveraging our prior establishment of functional cell derivation from CaMPs (Yang et al, 2022), we applied the Hnf4α+Foxa3-mediated hepatocyte-like cell (iHep) induction protocol (Lim et al, 2016) to generate albumin-positive (Alb^+^) and cytochrome P450 3a11-positive (Cyp3a11^+^) iHeps, assessing the impact of IRAK4 inhibitors. Pharmacological inhibition of IRAK4 (IR1/IR2) significantly enhanced hepatocytic induction, evidenced by robustly elevated Alb and Cyp3a11 mRNA expression compared to DMSO controls (Fig. 9A). In detail, both IR1 and IR2 treatments significantly increased Alb expression and colony formation. IR2 generated nearly 30 Alb⁺ colonies, a ~15-fold increase compared to the DMSO control (2–3 colonies; *p* < 0.001). Furthermore, IR2 enhanced Cyp3a11 expression and colony formation by 7–8-fold (*p* < 0.001). Although only IR2 robustly expanded the number of double-positive colonies, both IR1 and IR2 significantly elevated the proportion of Alb⁺Cyp3a11⁺ double-positive cells within individual colonies (Fig. 9B). Functional validation via Periodic Acid-Schiff (PAS) staining for glycogen storage and indocyanine green (ICG) uptake assays confirmed superior hepatocyte functionality in IRAK4-inhibited iHeps. IR1/IR2-treated cells exhibited significantly higher PAS-positive area (indicating enhanced glycogen metabolism) and increased ICG uptake (reflecting improved detoxification capacity) compared to controls (Fig. 9C,D). Global transcriptomic analysis revealed that IR2 upregulated gene set encompassed numerous critical hepatocyte-related transcription factors (Fig. 9E,F). GO analysis of upregulated genes demonstrated significant enrichment in metabolic, bile acid secretion and transport processes. Furthermore, GSEA analyses indicated that IR2 resulted in robust positive enrichment of fetal liver hepatoblasts cell fate, while causing significant negative enrichment of other cell types (Fig. 9H,I). Using the protocols for chemical induction of hepatocyte-like cells (CiHeps)(Bai et al, 2022), but replacing E-616452 with A-83-01 or A-83-01 + IRAK4i (i.e., 8 + IR2) throughout the process, we found that 8 + IR2 significantly induced higher mRNA levels of *Alb* and *Cyp3a11* than A-83-01 only (Fig. 9J). Collectively, these results indicated that IRAK4 was a fundamental barrier to hepatocyte reprogramming in chemical and transgenic induction methods. Its suppression—via specific pharmacological inhibitors—licenses the acquisition of hepatocyte-like identity and function, extending IRAK4’s pivotal role from MEF-to-XEN cell fate transition to MEF-to-hepatocyte lineage reprogramming.

## Discussion

The fundamental challenge in chemical reprogramming lies in overcoming the deeply entrenched molecular barriers that maintain somatic cell identity and restrict cellular plasticity. Our study identifies interleukin-1 receptor-associated kinase 4 (IRAK4), a central mediator of innate immune signaling, as a previously unrecognized barrier to multi-lineage reprogramming. Pharmacological inhibition (using IRAK4 inhibitors IR1, IR2, and Zi) or genetic knockdown of Irak4 significantly enhances the efficiency of converting MEFs through critical intermediate states, the chemically activated multi-lineage priming (CaMP) state and the extraembryonic endoderm (XEN)-like state, en route to pluripotency or directly towards functional neuron-like cells and hepatocyte-like cells (iHeps). Conversely, overexpression of IRAK4 imposes a dominant block on this reprogramming trajectory.

Our screening approach (Drug-seq2) using transcriptome profiling identified IRAK4 inhibitors IR1, IR2, and Zi as potent enhancers of XEN-like colony formation, with induction accompanied by robust co-expression of key XEN master regulators (Sox17, Gata4, Sall4, Foxa2). Dose-response analyses established optimal concentrations, and kinase assays confirmed that IRAK4 inhibition (Fig. 2I) represents a pathway independent of known ALK5/CDK8-targeting compounds such as E-616452 (Li et al, 2023). This positions IRAK4 inhibition as a mechanistically distinct enhancer of reprogramming initiation.

Our findings demonstrate that IRAK4 functions as a stabilizer of somatic identity, similar to CDK8 (repressing lineage-priming transcription factors) (Li et al, 2023) or epigenetic regulators like SETDB1 (depositing the repressive H3K9me3 mark at pluripotency-associated loci) (Chen et al, 2023) and KAT3A/KAT3B/KAT6A (stabilizing somatic networks via H3K27 hyperacetylation) (Wang et al, 2025), but via a potentially different mechanism: increasing chromatin accessibility and modulating cell cycle kinetics. ATAC-seq analyses demonstrate that IRAK4 inhibition promotes a global shift toward more accessible chromatin states during XEN-like cell induction, consistent with the transcriptional activation observed in our RNA-seq data. While our ATAC-seq data reveal a strong correlation between IRAK4 inhibition and increased chromatin accessibility, we cannot conclude that it is sufficient for the reprogramming phenotype. The observed accessibility changes provide a plausible mechanistic framework for future investigations, such as testing whether direct modulation of chromatin remodelers can phenocopy IRAK4 inhibition. The identification of shared TF-binding motifs associated with multi-lineage and pluripotency factors further supports the conclusion that IRAK4 suppression facilitates cell fate transition toward a chemically induced pluripotent state (Fig. 5). The observed IRAK4-dependent lengthening of G2/M and shortening of G0/G1 phases upon its inhibition aligns with established paradigms linking cell cycle remodeling to enhanced reprogramming efficiency (Guo et al, 2025; Liuyang et al, 2023). An extended G2/M phase is often associated with increased chromatin plasticity and heightened expression of factors conducive to pluripotency acquisition or lineage commitment, potentially due to a more open chromatin state during DNA replication and repair processes inherent to this phase (Gonzales et al, 2015; Guo et al, 2025; Ruiz et al, 2011). Conversely, a shortened G0/G1 phase may limit the time window during which pro-differentiation or pro-senescence signals, prevalent in somatic cells, can reinforce the original identity or trigger exit from the cell cycle (Gonzales et al, 2015; Guo et al, 2025; Ruiz et al, 2011). Thus, IRAK4 inhibition appears to create a permissive cell cycle “sweet spot” that facilitates the silencing of somatic programs and the activation of the multi-lineage transcriptional networks characteristic of the CaMP and XEN-like states. These findings further align with the established cell cycle signature of pluripotent cells (characteristically possessing a shortened G1 phase and extended G2/M phase) in contrast to somatic cells, which exhibit prolonged G1 and abbreviated G2/M phases (Ruiz et al, 2011). Previous studies have demonstrated that suppression or loss of TP53 facilitates somatic cell reprogramming toward induced pluripotent stem cells (iPSCs) (Hong et al, 2009; Kawamura et al, 2009; Marión et al, 2009; Utikal et al, 2009) by enhancing cell cycle under the OSKM inducible system. However, we didn’t observe that Trp53 knockdown induced XEN-like colonies in CFAE + 8 condition, which was consistent with the indispensable roles of Trp53 in human CiPSC induction (Cheng et al, 2026). Conversely, inhibitor-based cell cycle intervention completely abolished the formation of XEN-like colonies, indicating that proper cell cycle progression is indeed an important factor supporting reprogramming. While our data demonstrate a correlation between IRAK4 inhibition and cell cycle changes, we cannot conclude that cell cycle remodeling is the primary or direct mechanism underlying the enhanced reprogramming. Thus, we present cell cycle changes as an associated and potentially permissive event rather than a demonstrated causal pathway. Collectively, despite targeting different kinases (CDK8/ALK5 for E-616452 vs. IRAK4 for IR1/IR2), both E-616452 and IR1/IR2 converge on the suppression of key cell cycle checkpoints (G1/S and G2/M) and promote a similar multi-lineage primed state. This underscores cell cycle modulation as a critical, non-redundant axis for dismantling reprogramming barriers, suggesting that diverse signaling inputs can funnel through this central regulator of cellular plasticity.

While the G1/S transition was selected for mechanistic exploration based on its established role in cell fate control and the availability of well-characterized chemical modulators, GO analysis revealed other enriched categories, including extracellular matrix (ECM) organization and collagen metabolic processes that may also contribute to the observed cell fate activation. ECM has emerged as a critical regulator of cellular plasticity and lineage specification beyond its traditional structural functions. Recent studies have demonstrated that ECM components and their biomechanical properties actively modulate chromatin accessibility and transcription factor activity through mechanotransduction pathways, thereby influencing cell fate decisions. For instance, the ECM protein asporin has been shown to induce fetal-like reprogramming in intestinal epithelium via CD44-mediated TGF-β signaling (Iqbal et al, 2025), while mechanically heterogeneous hydrogels can direct osteogenic differentiation through epigenetic modulation involving EZH2 and H3K27me3 (Ling et al, 2026). Moreover, viscoelastic ECM substrates, which more accurately mimic native tissue mechanics than purely elastic materials, induce marked changes in nuclear architecture, reduce lamin A/C expression, and promote a global increase in euchromatin marks, thereby enhancing the reprogramming efficiency of fibroblasts into neurons and induced pluripotent stem cells (Wu et al, 2025). These findings suggest that ECM remodeling may act either in parallel with or upstream of cell cycle machinery to facilitate cell fate transitions. Given the enrichment of ECM-related terms in our RNA-seq data, it is possible that ECM dynamics contribute to the lineage conversion process observed in our system, potentially by providing permissive niche signals that cooperate with cell cycle progression to stabilize the reprogrammed state. Further investigation into integrating ECM manipulation with cell cycle modulation will be required to dissect the relative contributions and potential crosstalk between these two regulatory layers.

The profound ability of IRAK4 suppression to induce a CaMP state underscores its role as a broad-spectrum switch for cellular plasticity. Transcriptomic analyses revealed that Irak4 knockdown or IR1/IR2 treatment activated a series of transcriptional programs emblematic of diverse progenitor cell fates. This multi-lineage priming signature, characterized by the co-activation of markers spanning ectodermal, mesodermal, and endodermal germ layers (e.g., enriched GO terms for epithelial tube development, neurogenesis, gland development, renal/kidney development, digestive system development, and cell fate commitment), is likely the plastic state previously described during chemical reprogramming (Yang et al, 2022). Critically, IRAK4 overexpression produced the opposite effect: a global repression of developmental transcription factors, including the core XEN master regulators (*Sox17, Gata4, Sall4, Foxa2*), and a diversion of cells towards immunogenic trajectories, evidenced by the positive enrichment of immune cell development signatures. IRAK4 thus was a promising target for direct lineage reprogramming under a specific lineage induction cocktails or transgene combinations (Wang et al, 2021). It strongly supports the model that IRAK4 functions as a molecular barrier, constraining cells within a state refractory to lineage commitment, potentially poised for immune responses. This aligns perfectly with its canonical role as a critical node in Toll-like receptor (TLR) and interleukin-1 receptor (IL-1R) signaling, where IRAK4 activation nucleates signaling complexes leading to NF-κB and MAPK activation and the production of inflammatory mediators (Flannery and Bowie, 2010). Our prior work demonstrated that CDK8 inhibitors like MSC2530818 facilitate reprogramming partly by dampening inflammatory signaling cascades (Li et al, 2023). The finding that IRAK4 overexpression actively promotes immune-associated gene signatures reinforces the emerging concept that innate immune signaling pathways may act as guardians of somatic identity (Fig. 7I). Their activation may reinforce a defensive cellular state resistant to identity change, while their suppression (via CDK8 or IRAK4 inhibition) licenses the cell for reprogramming by removing this molecular brake and altering cell cycle progression. However, whether this immune gene induction directly causes the reprogramming blockade or represents an epiphenomenon remains unclear, and additional studies are needed to establish causality.

Importantly, IRAK4 inhibition enhances both pluripotent reprogramming and direct MEF-to-iHep conversion, improving efficiency and functional maturity, including elevated expression of hepatocyte markers (Albumin, Cyp3a11), glycogen storage, and detoxification capacity. We observed that IR2 exhibited higher efficiency than IR1 in inducing iHeps. This difference in reprogramming efficiency may reflect variations in their inhibitory potency, selectivity, and downstream signaling effects, which could differentially regulate the maintenance and expansion of hepatocyte-like cells. It seems that reprogramming to pluripotency and direct trans-differentiation into neuron-like or hepatocyte-like cells were two opposing processes. This apparent paradox can be resolved by viewing IRAK4 not as a fate-instructive molecule, but as a global stabilizer of somatic identity. Our data support a model wherein IRAK4 inhibition primarily acts by overcoming transcriptional barriers that lock cells in a fibroblast state. This licenses a state of multi-lineage priming (CaMP), which we and others have characterized as a plastic intermediate amenable to multiple fate choices (Li et al, 2023; Yang et al, 2022). The ultimate developmental trajectory of these plastic cells is then dictated by the specific extrinsic signals or contexts provided. In the context of the CFAE cocktail, which promotes pluripotency, the cells are directed toward an XEN-like and ultimately pluripotent state. In contrast, when exposed to specific chemical cues, the MEFs efficiently execute specific trans-differentiation programs. The mechanistic basis for IRAK4 inhibition in MEF-to-iHep trans-differentiation remains to be fully elucidated. Specifically, whether IRAK4 knockdown similarly alters chromatin accessibility or cell cycle dynamics, induces lineage-priming states, or suppresses NF-κB signaling in this context (as observed in CiPSC induction) requires further investigation. Nevertheless, the consistent enhancement of reprogramming efficiency across these distinct systems supports IRAK4 as a context-dependent barrier whose specific molecular mechanisms may vary with the starting cell type and target lineage. Therefore, the enhancement of both reprogramming and direct conversion by IRAK4 inhibition underscores its fundamental role as a gatekeeper of cellular plasticity, rather than a specifier of a particular lineage. This concept aligns with other broad-spectrum reprogramming barriers, such as CDK8 (Li et al, 2023), whose inhibition also facilitates diverse cell fate changes.

While our study establishes IRAK4 as a key barrier and elucidates its potential mechanism of action via chromatin accessibility and cell cycle control, several intriguing questions remain. First, although IRAK4 is implicated as a constitutive component of somatic cell fate maintenance in this study, its established role involves sensing extracellular chemokine signals to transduce intracellular immune-inflammatory responses. Whether IRAK4 functions identically to this canonical immune signaling pathway during chemical reprogramming remains undetermined. If yes, what is (are) the specific extracellular signal(s) and what is their origin? Second, the context-dependency of IRAK4’s barrier function warrants exploration in different systems (such as iHep or CiHep). We compared the efficiency of existing human reprogramming enhancers (such as A-485 and WM-80140 (Wang et al, 2025)) with that of IRAK4 inhibitors under the CFAE + 8 condition in MEFs. Notably, both A-485 and WM-80140 failed to induce XEN colonies, emphasizing the mechanistic differences among these enhancers. Testing IRAK4 inhibitors in established human CiPSC or direct conversion protocols is an essential next step.

In conclusion, we establish IRAK4 as a novel barrier that integrates chromatin accessibility and immune signaling with cell cycle control to constrain somatic identity. Targeting IRAK4 efficiently dismantles these barriers, enabling robust generation of pluripotent and lineage-specific cells, with broad implications for regenerative medicine and disease modeling.

## Methods

**Table:** Reagents and tools table

Reagent/resource	Reference or source	Identifier or catalog number
**Experimental models**		
HEK-293 cells (*H. sapiens*)	ATCC	CRL-1573
MEF	This study	N/A
Trelief® 5α Chemically Competent Cell	TSINGKE	Cat# TSC-C01
**Recombinant DNA**		
TetOne-puro-backbone	Addgene	Cat# 171123
TetOne-puro-Irak4	This paper	N/A
FU-tet-o-backbone	This paper	N/A
FU-tet-o-mHnf4a	This paper	N/A
FU-tet-o-mFoxa3	This paper	N/A
pLKO.1-scramble	This paper	N/A
pLKO.1-shIrak4-1	This paper	N/A
pLKO.1-shIrak4-2	This paper	N/A
**Antibodies**		
Anti-hSOX17	R&D Systems	Cat# AF1924; RRID:AB_355060
Anti-GATA-4	Santa Cruz	Cat# sc-25310; RRID:AB_627667
Anti-Sall4	Abcam	Cat# ab29112; RRID:AB_777810
Anti-FOXA2	Abcam	Cat# ab108422; RRID:AB_11157157
Anti-Alb	Beyotime Biotechnology	Cat# AF6183
Anti-CYP3A4	Proteintech	Cat# 67110-1-Ig;RRID: AB_2882414
Anti-Tuj1	Abcam	Cat# ab78078; RRID: AB_2256751
Anti-Syn1	CST	Cat# CST5297S;RRID: AB_2616578
Anti-vGlut2	SYSY	Cat# 135 402; RRID: AB_2187539
Anti-NeuN	Beyotime Biotechnology	Cat# AF1072
Donkey anti-Goat IgG (H+L) Cross-Adsorbed Secondary Antibody, Alexa Fluor™ 555	Thermo Fisher Scientific	Cat# A21432; RRID: AB_2535853
Donkey anti-Goat IgG (H+L) Cross-Adsorbed Secondary Antibody, Alexa Fluor™ 555	Thermo Fisher Scientific	Cat# A21202; RRID:AB_141607
Goat anti-Rabbit IgG H&L	Abcam	Cat# ab150077; RRID: AB_2630356
Goat anti-Rabbit Recobinant Secondary Antibody (H+L)	Proteintech	Cat# RGAR003; RRID: AB_3073507
Goat anti-Mouse Recombinant Secondary Antibody (H+L)	Proteintech	Cat#；RGAM003; RRID: AB_3068539
**Oligonucleotides and other sequence-based reagents**		
mIrak4_CDS_F1	Tsingke	tcctaccctcgtaaagaattcgccaccATGAACAAGCCGTTGACACC
mIrak4_CDS_R1	Tsingke	ccagtttatcgacttggatccTTAAGCAGACATCTCTTGTAGCAGC
Scramble-F	Tsingke	CCGGGTGACATTAGCTACCATACATTCAAGAGATGTATGGTAGCTAATGTCACTTTTTG
Scramble-R	Tsingke	AATTCAAAAAGTGACATTAGCTACCATACATCTCTTGAATGTATGGTAGCTAATGTCAC
shIrak4-F1	Tsingke	ccggGCTGGATATTAAAGAAGAGATCTCGAGATCTCTTCTTTAATATCCAGCTTTTTg
shIrak4-R1	Tsingke	aattcAAAAAGCTGGATATTAAAGAAGAGATCTCGAGATCTCTTCTTTAATATCCAGC
shIrak4-F2	Tsingke	ccggCCTGTGCTTAGTGTATGCTTACTCGAGTAAGCATACACTAAGCACAGGTTTTTg
shIrak4-R2	Tsingke	aattcAAAAACCTGTGCTTAGTGTATGCTTACTCGAGTAAGCATACACTAAGCACAGG
qGapdh_F1	Tsingke	AGGTCGGTGTGAACGGATTTG
qGapdh_R1	Tsingke	TGTAGACCATGTAGTTGAGGTCA
qSox17_F1	Tsingke	GTCAACGCCTTCCAAGACTTG
qSox17_R1	Tsingke	GTAAAGGTGAAAGGCGAGGTG
qSall4_F1	Tsingke	CCCTGGGAACTGCGATGAAG
qSall4_R1	Tsingke	TCAGAGAGACTAAAGAACTCGGC
qGata4_F1	Tsingke	CCCTACCCAGCCTACATGG
qGata4_R1	Tsingke	ACATATCGAGATTGGGGTGTCT
qFoxa2_F1	Tsingke	CCCTACGCCAACATGAACTCG
qFoxa2_R1	Tsingke	GTTCTGCCGGTAGAAAGGGA
qmIrak4_F1	Tsingke	CATACGCAACCTTAATGTGGGG
qmIrak4_R1	Tsingke	GGAACTGATTGTATCTGTCGTCG
qHnf4a-all-F1	Tsingke	CACGCGGAGGTCAAGCTAC
qHnf4a-all-R1	Tsingke	CCCAGAGATGGGAGAGGTGAT
qHnf4a_en-F1	Tsingke	tctgtaagcactgacctggg
qHnf4a_en-R1	Tsingke	ccacccactctccacattct
qFoxa3-all-F1	Tsingke	CTACATGACCTTGAACCCACTC
qFoxa3-all-R1	Tsingke	GGGCTACATACCCGGAAGC
qFoxa3_en-R1	Tsingke	agcacttccaaacgcatctg
qHnf4a_en-F1	Tsingke	tctgtaagcactgacctggg
qAlb-F1	Tsingke	GCCACCATTTGAAAGGCCAG
qAlb-R1	Tsingke	TCACACCATCAAGCTTCGGG
qCyp3a11-F1	Tsingke	CCGAGTGGATTTTCTTCAGC
qCyp3a11-R1	Tsingke	GAGCCTCATCGATCTCATCC
qTubb3-F1	Tsingke	TAGACCCCAGCGGCAACTAT
qTubb3-R1	Tsingke	GTTCCAGGTTCCAAGTCCACC
qSlc17a7-F1	Tsingke	GGTGGAGGGGGTCACATAC
qSlc17a7-R1	Tsingke	AGATCCCGAAGCTGCCATAGA
qGabbr1-F1	Tsingke	ACGTCACCTCGGAAGGTTG
qGabbr1-R1	Tsingke	CACAGGCAGGAAATTGATGGC
qGabbr2-F1	Tsingke	AAGACCCCATAGAGGACATCAA
qGabbr2-R1	Tsingke	GGGTGGTACGTGTCTGTGG
qRbfox3-F1	Tsingke	GGCTCCAAGGTCAATAATGCC
qRbfox3-R1	Tsingke	CCACAGGGTTTAGCTTCCAGC
qChat-F1	Tsingke	CCATTGTGAAGCGGTTTGGG
qChat-R1	Tsingke	GCCAGGCGGTTGTTTAGATACA
**Chemicals, enzymes and other reagents**		
bFGF	PeproTech	Cat# AF-100-18B
Human FGF4	Peprotech	Cat# AF-100-31-25UG
Human HGF	Peprotech	Cat# 100-39-2
EGF	Peprotech	Cat# 100-47
Human BMP4	Peprotech	Cat# 120-05-100
CHIR99021	Selleck	Cat# S2924; CAS: 252917-06-9
E-616452	TargetMol	Cat# T6337; CAS 446859-33-2
Forskolin	TargetMol	Cat# T2939; CAS 66575-29-9
EPZ00477	TargetMol	Cat# T3081; CAS: 1338466-77-5
AM580	TargetMol	Cat# T5854; CAS: 102121-60-8
IRAK inhibitor 1	TargetMol	Cat# T5094; CAS: 1042224-63-4
IRAK4-IN-1	TargetMol	Cat# T4140; CAS: 1820787-94-7
5-Iodotubercidin	TargetMol	Cat# T6745; CAS: 24386-93-4
UNC2025	TargetMol	Cat# T7007; CAS: 1429881-91-3
Nintedanib esylate	TargetMol	Cat# T5001; CAS: 656247-18-6
Dovitinib lactate	TargetMol	Cat# T7104; CAS: 692737-80-7
Zimlovisertib	TargetMol	Cat# TQ0167; CAS: 1817626-54-2
MSC230818	TargetMol	Cat# TQ0266; CAS: 1883423-59-3
ISX-9	MCE	Cat# HY-12323; CAS: 832115-62-5
Dexamethosone	Selleck	Cat# S5956; CAS: 2392-39-4
A-83-01	Selleck	Cat# S7692; CAS: 909910-43-6
EndoFree Maxi Plasmid Kit	TIANGEN	Cat# DP117
MolPure® Endo-free Plasmid Mini Kit	YEASEN	Cat# 19021ES50
FastPure Gel DNA Extraction Mini Kit	Vazyme	Cat# DC301-01
2 × Phanta Max Master Mix	Vazyme	Cat# P515-01
T4 DNA Ligase	NEB	Cat# M0202T
Seamless Cloning and Assembly Kit	OBIO	Cat# OBCR(A) 20133001
TruePrep DNA Library Prep Kit V2 for Illumina	Vazyme	Cat# TD502-01
RNAeasy™ Plus Kit	Beyotime Biotechnology	Cat# R0032
HiScript III RT SuperMix for qPCR	Vazyme	Cat# R333-01
Taq Pro Universal SYBR qPCR Master Mix	Vazyme	Cat# Q712-03
Cell cycle staining kit	MULTI SCIENCES	Cat# CCS012
BamH I		Cat# R0136T
EcoR I-HF	NEB	Cat# R3101T
AgeI-HF	NEB	Cat# R3552S
T4 DNA Ligase	NEB	Cat# M0202T
Knockout™ SR	Thermo Fisher Scientific	Cat# 10828028
KnockOut™ DMEM	Thermo Fisher Scientific	Cat# 10829018
High-glucose DMEM	Thermo Fisher Scientific	Cat# 10829018
Trypsin 0.25% EDTA	Thermo Fisher Scientific	Cat# 15090046
2.5 %Trypsin	Thermo Fisher Scientific	Cat# 15090046
FBS	VISTECH	Cat# SE100-B
GlutaMax-1	Gibco	Cat# 35050087
NEAA	Thermo Fisher Scientific	Cat# 11140050
DMSO	Solarbio	Cat# D8371
β-mercaptoethanol	Solarbio	Cat# 60-24-2
N2 supplement	Gibco	Cat# 17502048
B27 SUPPLEMENT	Gibco	Cat# 17504044
PEI Max	PolySciences	Cat# 24765-2
Opti-MEM	Gibco	Cat# 31985070
DMEM/F-12	Gibco	Cat# 11330057
Penicillin-Streptomycin SOL20 X 100 ML	Gibco	Cat# 15140163
Doxycycline hyclate	Solarbio	Cat# D8960; CAS:24390-14-5
ITS	Thermo Fisher Scientific	Cat# 41400045
4% Paraformaldehyde	DINGGUO	Cat# AR-0211
Nicotinamide	Sigma	Cat# N0636-100G
Periodic acid-Schiff	Solarbio	Cat# G1360
Indocyanine green	Solarbio	Cat# I8870
**Software**		
Hisat2 (v2.0.5)	https://daehwankimlab.github.io/hisat2/	N/A
Subread tool (v1.5.0-p3)	https://github.com/ShiLab-Bioinformatics/subread	N/A
R version 5.4.0	https://www.r-project.org	N/A
RStudio (2023.6.1.524)	https://www.rstudio.com	N/A
DESeq2 (v1.34.0)	https://bioconductor.org/packages/release/bioc/html/DESeq2.html	N/A
clusterProfiler (v4.14.6)	https://bioconductor.org/packages//release/bioc/html/clusterProfiler.html	N/A
ClusterGVis (v0.1.3)	https://github.com/junjunlab/ClusterGVis	N/A
ggplot2 (v3.5.2)	https://ggplot2.tidyverse.org/	N/A
scRNAtoolVis (v.0.1.0)	https://github.com/junjunlab/scRNAtoolVis	N/A
Bowtie2(v2.5.4)	http://bowtie-bio.sourceforge.net/bowtie2/index.shtml	N/A
Samtools (v1.9)	http://samtools.sourceforge.net/	N/A
deepTools (v3.5.6)	https://deeptools.readthedocs.io/en/latest/	N/A
MACS2 (v2.2.9.1)	https://pypi.org/project/MACS2/	N/A
HOMER (v5.1)	http://homer.ucsd.edu/homer/	N/A
GraphPad Prism 9	GraphPad Software Inc.	N/A
**Other**		
RNA-Seq	This paper	GEO: GSE303071, GSE303072, GSE303073, GSE303074
ATAC-seq	This paper	GEO: GSE327913

### Isolation and culture of mouse embryonic fibroblasts (MEFs)

Mouse embryonic fibroblasts (MEFs) were isolated and cultured as previously described (Li et al, 2023; Zhao et al, 2015). Briefly, MEFs were isolated from E13.5 embryos of ICR wild-type mice. Embryos were decapitated. Head, limbs and viscera were removed. The remaining tissues were mechanically dissected and digested in 0.25% trypsin-EDTA (Thermo Fisher Scientific, Cat# 15090046) at 37 °C for 10 min. Dissociated cells were collected and cultured in MEF medium consisting of high-glucose Dulbecco’s modified Eagle’s medium (DMEM) (Thermo Fisher Scientific, Cat# 10829018) supplemented with 10% fetal bovine serum (FBS), 1% GlutaMAX, 1% nonessential amino acids (NEAA) (Thermo Fisher Scientific, Cat# 11140050), 0.055 mM β-mercaptoethanol, and 1% penicillin-streptomycin.

### Plasmid construction

For the construction of Tet-one-mouse Irak4, full-length cDNAs encoding Irak4 was cloned into pLVX-TetOne-Puro backbone (Addgene Catalog #171123) after EcoR I (NEB, Cat# R3101T) and BamH I (NEB, Cat# R0136T) digestion. FU-tet-o-mouse Foxa3 and FU-tet-o-mouse Hnf4a plasmids were from a previous paper (Li et al, 2023). For the construction of mouse Irak4 knockdown plasmids, shRNA oligos were annealed from 100 °C to 25 °C by decreasing 1 °C per minute in a PCR cycler and then cloned into pLKO.1-TRC cloning vector (Addgene Catalog #10878) after EcoR I and Age I (NEB, Cat# R3552S) digestion. All primers or shRNA oligos are listed in the Reagents and tools table. Reagents and kits used in plasmid construction include EndoFree Maxi Plasmid Kit (TIANGEN, Cat# DP117), MolPure® Endo-free Plasmid Mini Kit (YEASEN, Cat# 19021ES50), FastPure Gel DNA Extraction Mini Kit (Vazyme, Cat# DC301-01), 2 × Phanta Max Master Mix (Vazyme, Cat# P515-01), T4 DNA Ligase (NEB, Cat# M0202T), and Seamless Cloning and Assembly Kit (OBIO, Cat# OBCR(A) 20133001). Competent Cells used in plasmid construction were Trelief® 5α Chemically Competent Cell (TSINGKE, Cat# TSC-C01).

### Generation of XEN-like cells

XEN-like cells and CiPSC were induced from MEFs via a stage-specific approach as previously described (Li et al, 2023; Zhao et al, 2015). During the process, MEF medium consisting of DMEM/high glucose (Gibco), 10% FBS, 1% NEAAs, 1% GlutaMAX, 0.055 mM β-mercaptoethanol, 1% penicillin–streptomycin was used to seed MEFs. XEN basal medium consisting of KnockOut DMEM, 10% FBS, 10% KnockOut Serum Replacement (KSR), 0.055 mM β-mercaptoethanol, 1% NEAA, 1% GlutaMAX, 1% penicillin–streptomycin), 50 ng/mL bFGF (PeproTech, Cat# AF-100-18B) was used for cell reprogramming with specific chemical cocktails. For XEN-like induction, MEFs of passages 1–2 were seeded into 12-well plates at a density of 3.6 × 10^5^ cells per plate with MEF medium. The next day, MEF medium was changed to stage I medium. Stage I medium (C6FAE): XEN basal medium, 20 μM CHIR99021, 10 μM E-616452, 50 μM Forskolin, 0.05 μM AM580, and 5 μM EPZ004777 as previously described (Li et al, 2023; Yang et al, 2022). Stage I medium was changed every 4 days for 12 days. Cells were re-plated (1:6) and incubated in stage I medium with 25 ng/mL bFGF, 10 μM CHIR99021, and 10 μM Forskolin for another 4 days. For testing the candidate chemicals from Drug-seq2 (Li et al, 2023), 2 μM candidates were added to C8FAE cocktails to induce XEN-like colonies for 12 days. For testing Irak4i function, 2 μM IR1 or 5 μM IR2 were added to C8FAE. Cells on day 12 or 16 were harvested for immunofluorescence or mRNA quantification.

### Generation of neuron-like cells from the CaMP state

Neuron-like cells were induced from CaMP state cells as previously described (Yang et al, 2022). MEFs were seeded at 30,000 cells per well in 12-well plates and maintained in MEF culture medium. On day 0, the medium was switched to CFAE + 8, CFAE + 8 + 2 μM IR1, or CFAE + 8 + 5 μM IR2 medium. On day 4, cells were transferred to neuron-like cell reprogramming medium composed of neurobasal medium supplemented with 2% B27, 1% GlutaMAX, 1% penicillin-streptomycin, and the small-molecule cocktail CFI (3 μM CHIR99021, 10 μM Forskolin, and 10 μM ISX-9) for 16 days. Medium was refreshed every 4 days.

### Generation of hepatocyte-like cells

Hepatocyte-like cells were induced from MEFs as previously described (Bai et al, 2022; Li et al, 2023). Briefly, for the generation of transcription factors (TF)-induced hepatocytes, 5 × 10⁴ MEFs per well (24-well plate) were transduced for 24 h with tetracycline-inducible lentiviral vectors expressing *Hnf4a* and *Foxa3*. Following transduction, the medium was replaced with MEF medium. After an additional 24 h, cultures were switched to standard hepatocyte culture medium (HCM) containing DMEM/F-12, 10% FBS, 1% ITS, 1% penicillin-streptomycin, 1% GlutaMAX, 0.1 μM dexamethasone, 10 mM nicotinamide, 10 ng/mL FGF4, 20 ng/mL HGF, 20 ng/mL EGF, 10 ng/mL BMP4, 2 μM A-83-01, and 3 μM CHIR99021—supplemented with or without Irak4 inhibitors (2 μM IR1 or 5 μM IR2) for 24 days. The medium was refreshed every 4 days. For the generation of chemically induced hepatocytes. MEFs were plated in 12-well plates at 3 × 10⁴ cells per well in MEF medium. At initiation (Day 0), the medium was replaced with Stage I XEN chemically defined medium. On Day 4, cultures were transitioned to HCM-based induction medium supplemented with 20 µM CHIR99021, 10 µM E-616452, and 50 µM Forskolin (C6F_H). On Day 8, compounds were reduced to 3 µM CHIR99021, 2 µM E-616452, and 2 µM Forskolin(C6F_L), with medium changes every 4 days until Day 24. For testing IRAK4 inhibitor (IR2), E-616452 was substituted with 2 µM A-83-01 only or 2 µM A-83-01 plus 5 µM IR2.

### PAS staining and ICG uptake assays

These assays were conducted according to previously described methods (Bai et al, 2022). Cells underwent staining with either periodic acid-Schiff (PAS; Solarbio, Cat# G1360) according to the manufacturer’s protocol or 1 mg/mL indocyanine green (ICG; Solarbio, Cat# I8870) for 1 h at 37 °C. Following three PBS washes, images were acquired using a Leica microscope.

### Bulk RNA-seq data analysis

Fragmented and randomly primed cDNA libraries were constructed and sequenced (2 × 150 bp paired-end) on an Illumina HiSeq X Ten system. Bulk RNA-seq reads were aligned and processed to generate a gene-level count matrix for all samples using hisat2 (v2.0.5) (Kim et al, 2019) and featureCounts of the Subread tool (v1.5.0-p3) (Liao et al, 2014). The count matrix was imported into the DESeq2 package (v1.34.0) (Love et al, 2014) in R (v5.4.0) for differential expression analysis. Genes with |log2 fold change|>1 and *p* value <0.05 were defined as differentially expressed genes (DEGs). Functional enrichment analysis of DEGs was performed using the clusterProfiler package (v4.1.6) (Wu et al, 2021) for Gene Ontology (GO) biological processes, Kyoto Encyclopedia of Genes and Genomes (KEGG) pathways, and gene set enrichment analysis (GSEA). Heatmaps, cluster line plots, GO, and Kyoto KEGG enrichment analyses were produced with the ClusterGVis package (v0.1.3). Volcano plots were generated using the ggplot2 package (v3.5.2).

### Single-cell RNA-sequencing data processing

The publicly available scRNA-seq dataset GSE144097, generated by Smart-seq2, was analyzed using the Seurat R package (v5.4.0). Quality control filtering was applied to cells with 500–10,000 detected genes. This was followed by data normalization, variable gene selection, and scaling. t-SNE dimensionality reduction and clustering were performed based on the first 15 principal components (PCA). The expression of the genes of interest was visualized on t-SNE plots with the scRNAtoolVis package (v.0.1.0). To assess the co-expression of genes associated with the three germ layers, we used the 10th percentile of non-zero expression values for each gene as a dynamic threshold, calculated the proportion of co-expressing cells for different gene combinations, and visualized the results.

### ATAC-seq library preparation and data analysis

ATAC-seq library was conducted as previously described (Li et al, 2023). Briefly, a total of 1 × 10⁴ cells were lysed in buffer containing 10 mM Tris-HCl (pH 7.4), 10 mM NaCl, 3 mM MgCl₂, and 0.5% NP-40. Nuclei were collected by centrifugation at 500×*g* for 5 min at 4 °C. Subsequently, sequencing libraries were prepared using the TruePrep DNA Library Prep Kit V2 for Illumina (TD502-01, Vazyme) according to the manufacturer’s instructions. For ATAC-seq data analysis, reads were aligned to the mm39 reference genome using Bowtie2 (v2.5.4) (Langmead and Salzberg, 2012), excluding unmapped and non-uniquely mapped reads. BAM files were generated by Samtools (v1.9) (Li et al, 2009), and PCR duplicates were removed using Picard MarkDuplicates. BamCoverage (bamCoverage -p 50 --numberOfProcessors max --binSize 50 --normalizeUsing RPKM --bam --outFileName) and computeMatrix (computeMatrix reference-point -S file.bw -R file.bed --referencePoint center -a 5000 -b 5000 --outFileName -p max) of deepTools (v3.5.6)(Ramírez et al, 2016) were used to visualize normalized read counts (RPKM). Peak calling were performed using MACS2 (v2.2.9.1) (Gaspar, 2018; Zhang et al, 2008) and HOMER (v5.1)(Heinz et al, 2010). Motif analysis results were obtained from HOMER and filtered at a threshold of *P* < 1e-10. We identified the shared motifs between the “6 vs 8” and “8 + IR2 vs 8” comparison groups. From this intersection, we specifically selected markers representing the three germ layers: endoderm, mesoderm, and ectoderm. These markers were visualized using dumbbell plots with the ggplot2 package (v.3.5.2). The remaining motifs in the intersection were presented in a separate dumbbell plot. Subsequently, we analyzed three subsets: only_6, only_8 + IR2, and the intersection. GO and KEGG enrichment analyses were performed using the compareCluster function in the clusterProfiler package (v.4.14.6). All enrichment results were visualized using ggplot2.

### Immunofluorescence

Immunofluorescence was performed as previously described (Yang et al, 2020). The primary antibodies used in this study included anti-SOX17 (R&D, AF1924, 1:200), anti-GATA4 (Santa Cruz, sc-25310, 1:300), anti-SALL4 Abcam, (ab29112, 1:200), anti-FOXA2 (Abcam, ab108422, 1:200), anti-ALB (Beyotime Biotechnology, AF6183, 1:100), anti-CYP3A4 (Proteintech, 67110-1-Ig, 1:400), anti-Tuj1 (Abcam, ab78078, 1:1000), anti-Syn1 (CST, CST5297S 1:200), anti-vGlut2 (SYSY, 135 402, 1:500), and anti-NeuN (Beyotime, AF1072, 1:100). The secondary antibodies were included donkey anti-goat IgG (H + L) cross-adsorbed secondary antibody (Thermo Fisher Scientific, A21432,1:2000), goat anti-Rabbit IgG H&L (Abcam, ab150077,1:800), goat anti-Rabbit Recobinant Secondary Antibody (H + L) (Proteintech, RGAR003,1:500), donkey anti-mouse IgG (H + L) cross-adsorbed secondary antibody (Thermo Fisher Scientific, A21202,1:2000), and goat anti-Mouse Recombinant Secondary Antibody (H + L) (Proteintech, RGAM003,1:500). Images were captured using a Leica DMi8 fluorescence microscope.

### Quantitative PCR

Total RNA was isolated from cells using the RNAeasy™ Plus Kit (Beyotime Biotechnology, R0032) according to the manufacturer’s protocol. Subsequently, 1 µg of total RNA was reverse-transcribed into cDNA using the HiScript III RT SuperMix for qPCR (Vazyme, R333-01) under the following conditions: 50 °C for 15 min, followed by enzyme inactivation at 85 °C for 5 s. Quantitative real-time PCR (qRT-PCR) was performed using Taq Pro Universal SYBR qPCR Master Mix (Vazyme, Q712-03) on Applied Biosystems instruments (Life Technologies, Quant Studio 5 and 6). RT-qPCR primer sequences are provided in the Reagents and tools table.

### Cell cycle analysis

Cell cycle analysis was conducted using a commercial cell cycle staining kit (MULTI SCIENCES, CCS012) following the manufacturer’s instructions. Briefly, reprogrammed cells at days 8 or 10 were gently dissociated with trypsin, and cell pellets were collected at a density of 1 × 10⁶ cells per sample. Pellets were resuspended in a 250 μL PBS:750 μL ice-cold ethanol mixture to fix cells, then incubated at 4 °C overnight. The following day, fixed cells were centrifuged, resuspended in ice-cold PBS, and washed twice with cold PBS to remove residual ethanol. Cell pellets were resuspended in staining solution containing 100 µg/mL RNase A and 100 µg/mL propidium iodide (PI) in PBS and incubated at 37 °C for 30 min in the dark. Flow cytometry analysis of PI-stained cells was performed within 1 h of staining to preserve signal stability using a flow cytometer (Becton Dickinson, Franklin Lakes, USA).

### Statistical analysis

Statistical analyses were performed using GraphPad Prism 9.5.1 (GraphPad Software, San Diego, CA). Differences between the two groups were analysed using unpaired Student’s *t*-tests. Comparisons among three or more groups were assessed by one-way analysis of variance (ANOVA).

## Supplementary information

Appendix

Peer Review File

Source data Fig. 1

Source data Fig. 2

Source data Fig. 4

Source data Fig. 6

Source data Fig. 7

Source data Fig. 8

Source data Fig. 9

Figure EV1 Source Data

Expanded View Figures

## Data Availability

Supplemental sequencing data are available in the Gene Expression Omnibus database under accession numbers GSE303071, GSE303072, GSE303073, GSE303074, and GSE327913. All microscopy images of this paper are collected in the following database record: biostudies: S-BIAD3324. The source data of this paper are collected in the following database record: biostudies:S-SCDT-10_1038-S44319-026-00855-9.
