# Supplementary material for: IRAK4 constrains cellular plasticity during chemically-induced cell fate reprogramming into multiple lineages
Source: EMBO Rep. 2026 Jul 15;27(16):4903–34. doi: 10.1038/s44319-026-00855-9 (PMC13503836; doi:10.1038/s44319-026-00855-9)
Supplement: Supplementary file 1 [file 44319_2026_855_MOESM11_ESM.pdf]

Expanded View Figures

Figure EV1. Identifying the candidate chemicals from the DRUG-seq2 screen that initiate MEF-XEN reprogramming.

(**A**) The morphologies of colonies in CFAE + 8 plus candidate chemicals from the DRUG-seq2 screen or in CFAE + 6 on day 12. (C CHIR99021, F Forskolin, A AM580, E EPZ04777, 6 E-616452, 8 A-83-01, Dov dovitinib lactate, Nin nintedanib esylate, UNC UNC2025, ITU 5-iodotubercidin, IR1 IRAK inhibitor 1, IR2 IRAK4-IN-1, Zi zimlovisertib, MSC MSC2530818). (**B**) Quantifying colony numbers in (**A**). Data were represented as mean ± SD. $n = 3$ biological replicates, respectively. (**C–E**) Individual raw images and merged panel related to Fig. 1A. Immunofluorescence staining of XEN master regulators Sox17, Gata4, Sall4, and Foxa2 in CFAE + 8 plus ITU/IR1/IR2/Zi/MSC or CFAE + 6, respectively. Scale bar: 500 µm. Source data are available online for this figure.

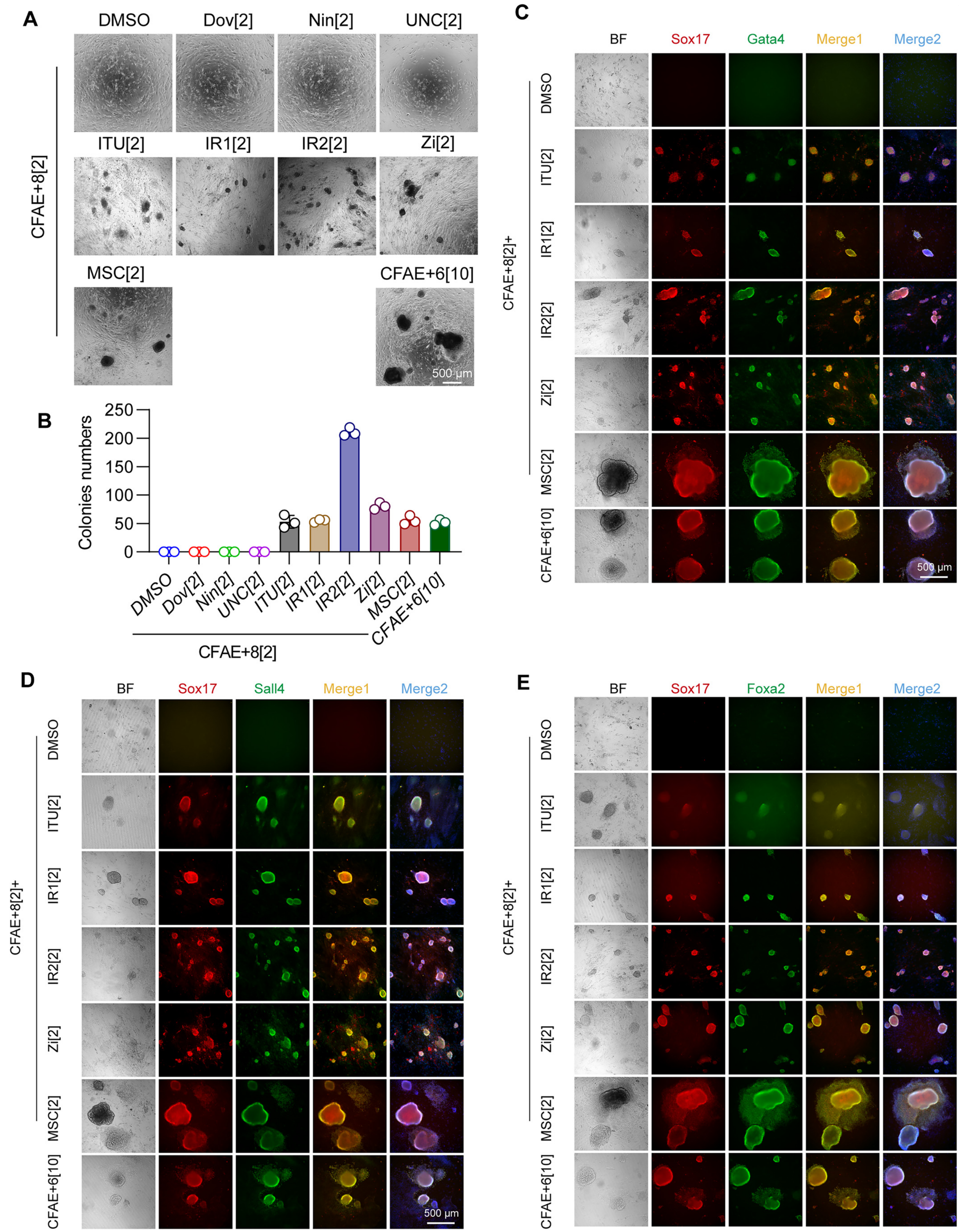
A
DMSO
Dov[2]
Nin[2]
UNC[2]
ITU[2]
IR1[2]
IR2[2]
Zi[2]
MSC[2]
CFAE+6[10]
CFAE+8[2]
500 μm
B
Colonies numbers
250
200
150
100
50
0
DMSO
Dov[2]
Nin[2]
UNC[2]
ITU[2]
IR1[2]
IR2[2]
Zi[2]
MSC[2]
CFAE+6[10]
CFAE+8[2]
C
BF
Sox17
Gata4
Merge1
Merge2
CFAE+8[2]+
500 μm
D
BF
Sox17
Sall4
Merge1
Merge2
CFAE+8[2]+
500 μm
E
BF
Sox17
Foxa2
Merge1
Merge2
CFAE+8[2]+

© The Author(s)

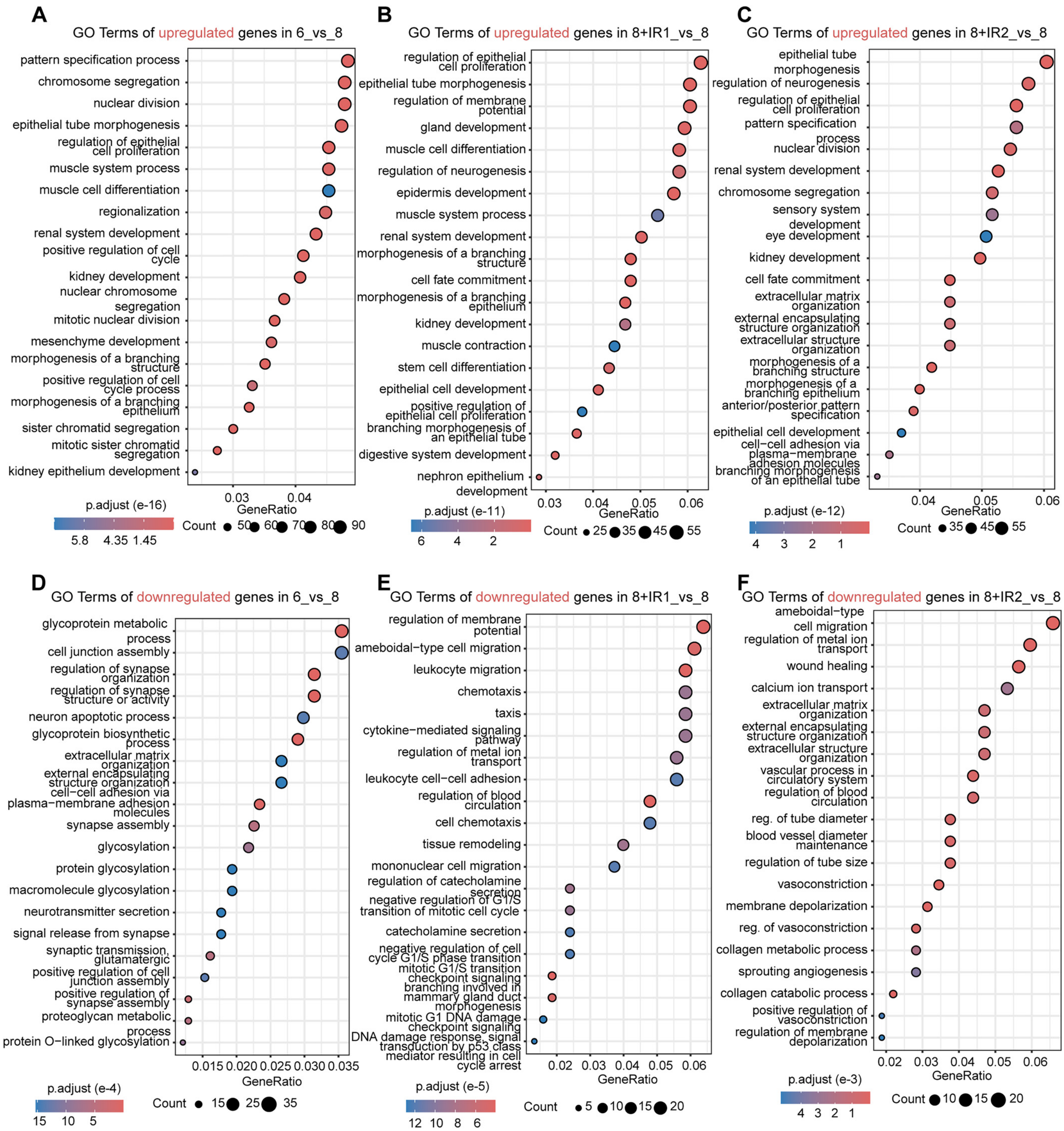

Figure EV2. Gene ontology analysis of DEGs in 6, 8 + IR1, or 8 + IR2 versus 8.

Biological processes (BP) of gene ontology (GO) enrichment in upregulated (**A–C**) or downregulated (**D–F**) genes of 6, 8 + IR1, or 8 + IR2 versus 8. The *P* value was calculated using the Hypergeometric test. Basal medium: CFAE. Optimal concentration determination of IR2 during MEF-XEN reprogramming. Related to Figs. 3 and 4.

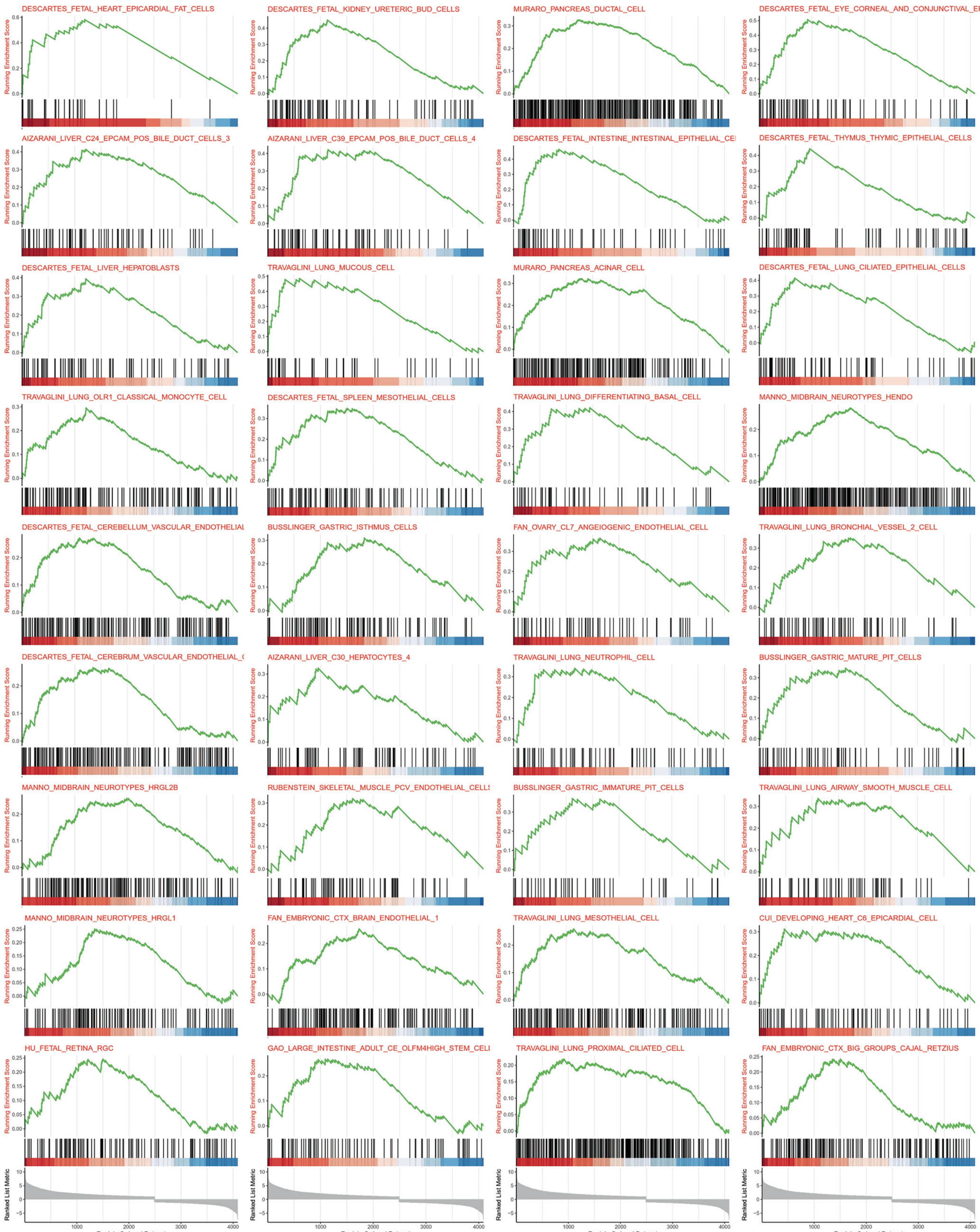

Figure EV3. GSEA of multi-lineage fate pathways in both CFAE + 6 and CFAE + 8 + IR1/IR2 versus the CFAE + 8 control.

 © The Author(s)

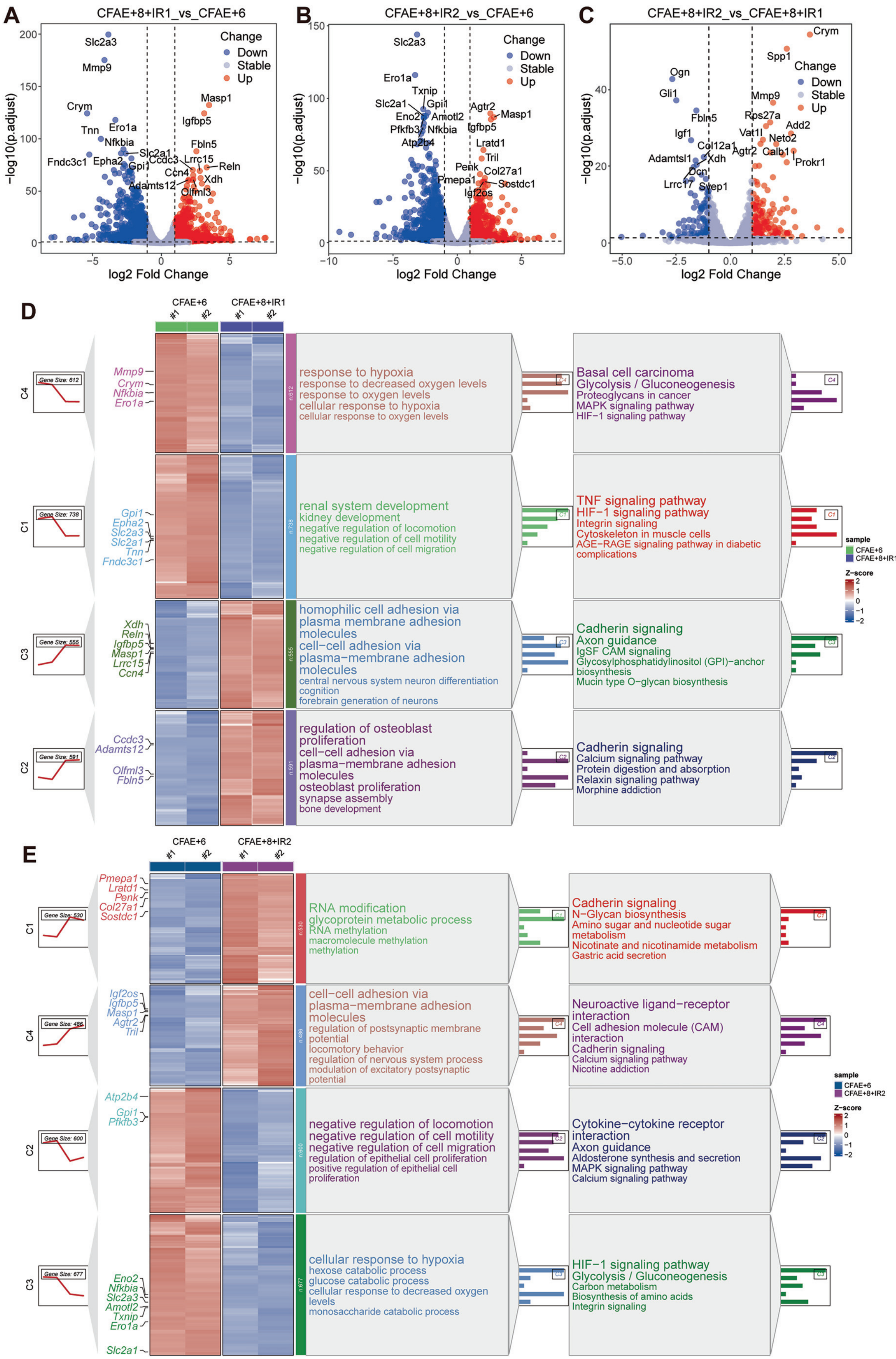
A
CFAE+8+IR1_vs_CFAE+6
B
CFAE+8+IR2_vs_CFAE+6
C
CFAE+8+IR2_vs_CFAE+8+IR1
Change
Down
Stable
Up
−log10(p.adjust)
log2 Fold Change
D
CFAE+6
CFAE+8+IR1
response to hypoxia
response to decreased oxygen levels
response to oxygen levels
cellular response to hypoxia
cellular response to oxygen levels
Basal cell carcinoma
Glycolysis / Gluconeogenesis
Proteoglycans in cancer
MAPK signaling pathway
HIF−1 signaling pathway
renal system development
kidney development
negative regulation of locomotion
negative regulation of cell motility
negative regulation of cell migration
TNF signaling pathway
HIF−1 signaling pathway
Integrin signaling
Cytoskeleton in muscle cells
AGE−RAGE signaling pathway in diabetic complications
homophilic cell adhesion via plasma membrane adhesion molecules
cell−cell adhesion via plasma−membrane adhesion molecules
central nervous system neuron differentiation
cognition
forebrain generation of neurons
Cadherin signaling
Axon guidance
IgSF CAM signaling
Glycosylphosphatidylinositol (GPI)−anchor biosynthesis
Mucin type O−glycan biosynthesis
regulation of osteoblast proliferation
cell−cell adhesion via plasma−membrane adhesion molecules
osteoblast proliferation
synapse assembly
bone development
Cadherin signaling
Calcium signaling pathway
Protein digestion and absorption
Relaxin signaling pathway
Morphine addiction
sample
Z−score
E
CFAE+6
CFAE+8+IR2
RNA modification
glycoprotein metabolic process
RNA methylation
macromolecule methylation
methylation
Cadherin signaling
N−Glycan biosynthesis
Amino sugar and nucleotide sugar metabolism
Nicotinate and nicotinamide metabolism
Gastric acid secretion
cell−cell adhesion via plasma−membrane adhesion molecules
regulation of postsynaptic membrane potential
locomotory behavior
regulation of nervous system process
modulation of excitatory postsynaptic potential
Neuroactive ligand−receptor interaction
Cell adhesion molecule (CAM) interaction
Cadherin signaling
Calcium signaling pathway
Nicotine addiction
negative regulation of locomotion
negative regulation of cell motility
negative regulation of cell migration
regulation of epithelial cell proliferation
positive regulation of epithelial cell proliferation
Cytokine−cytokine receptor interaction
Axon guidance
Aldosterone synthesis and secretion
MAPK signaling pathway
Calcium signaling pathway
cellular response to hypoxia
hexose catabolic process
glucose catabolic process
cellular response to decreased oxygen levels
monosaccharide catabolic process
HIF−1 signaling pathway
Glycolysis / Gluconeogenesis
Carbon metabolism
Biosynthesis of amino acids
Integrin signaling

Figure EV4. Differential gene expression and comparative transcriptomic analysis between indicated conditions.

(**A–C**) Volcano plot of CFAE + 8 + IR1, CFAE + 8 + IR2 versus CFAE + 6, or CFAE + 8 + IR2 versus CFAE + 8 + IR1 (**A–C**: $n = 2$ biological replicates, respectively). The *P* value was calculated using the Wald test. Significantly up- and down-regulated genes are defined by |log2FC|> 1 and *p*.adjust <0.05. (**D**, **E**) Heatmap, GO, and KEGG enrichments of differentially expressed transcripts in CFAE + 8 + IR1 or CFAE + 8 + IR2 versus CFAE + 6. Enriched GO-BP and KEGG pathways are displayed with font size scaled to significance.

 © The Author(s)

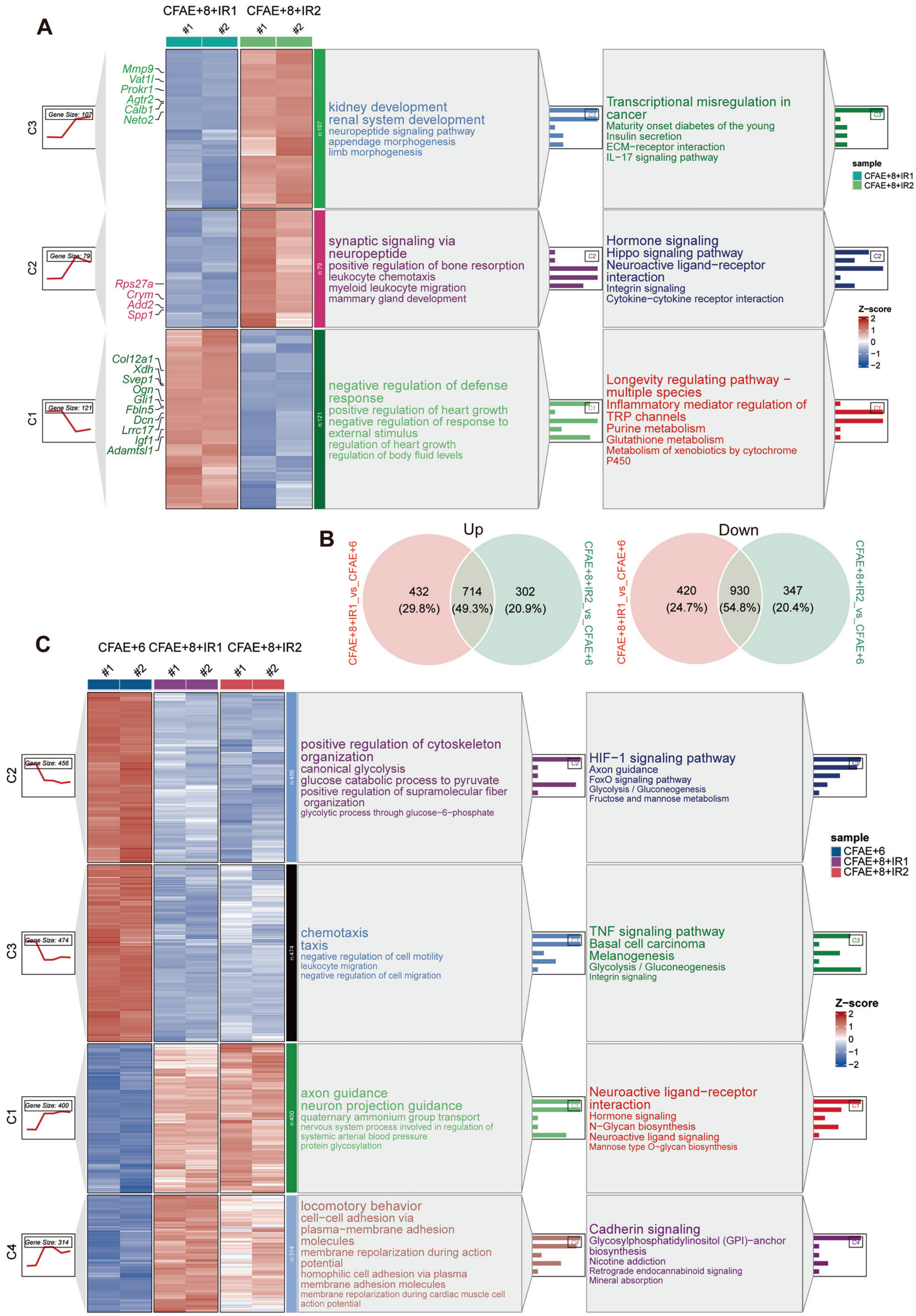
A
CFAE+8+IR1 CFAE+8+IR2
#1 #2 #1 #2
C3 Gene Size: 107
Mmp9 Vat1l Prokr1 Agtr2 Calb1 Neto2
kidney development
renal system development
neuropeptide signaling pathway
appendage morphogenesis
limb morphogenesis
Transcriptional misregulation in cancer
Maturity onset diabetes of the young
Insulin secretion
ECM−receptor interaction
IL−17 signaling pathway
sample
CFAE+8+IR1
CFAE+8+IR2
C2 Gene Size: 79
Rps27a Crym Add2 Spp1
synaptic signaling via neuropeptide
positive regulation of bone resorption
leukocyte chemotaxis
myeloid leukocyte migration
mammary gland development
Hormone signaling
Hippo signaling pathway
Neuroactive ligand−receptor interaction
Integrin signaling
Cytokine−cytokine receptor interaction
Z−score
C1 Gene Size: 121
Col12a1 Xdh Svep1 Ogn Gli1 Fbln5 Dcn Lrrc17 Igf1 Adamtsl1
negative regulation of defense response
positive regulation of heart growth
negative regulation of response to external stimulus
regulation of heart growth
regulation of body fluid levels
Longevity regulating pathway − multiple species
Inflammatory mediator regulation of TRP channels
Purine metabolism
Glutathione metabolism
Metabolism of xenobiotics by cytochrome P450
B
Up
CFAE+8+IR1_vs_CFAE+6
CFAE+8+IR2_vs_CFAE+6
432 (29.8%)
714 (49.3%)
302 (20.9%)
Down
420 (24.7%)
930 (54.8%)
347 (20.4%)
C
CFAE+6 CFAE+8+IR1 CFAE+8+IR2
C2 Gene Size: 456
positive regulation of cytoskeleton organization
canonical glycolysis
glucose catabolic process to pyruvate
positive regulation of supramolecular fiber organization
glycolytic process through glucose−6−phosphate
HIF−1 signaling pathway
Axon guidance
FoxO signaling pathway
Glycolysis / Gluconeogenesis
Fructose and mannose metabolism
sample
CFAE+6
CFAE+8+IR1
CFAE+8+IR2
C3 Gene Size: 474
chemotaxis
taxis
negative regulation of cell motility
leukocyte migration
negative regulation of cell migration
TNF signaling pathway
Basal cell carcinoma
Melanogenesis
Glycolysis / Gluconeogenesis
Integrin signaling
Z−score
C1 Gene Size: 400
axon guidance
neuron projection guidance
quaternary ammonium group transport
nervous system process involved in regulation of systemic arterial blood pressure
protein glycosylation
Neuroactive ligand−receptor interaction
Hormone signaling
N−Glycan biosynthesis
Neuroactive ligand signaling
Mannose type O−glycan biosynthesis
C4 Gene Size: 314
locomotory behavior
cell−cell adhesion via plasma−membrane adhesion molecules
membrane repolarization during action potential
homophilic cell adhesion via plasma membrane adhesion molecules
membrane repolarization during cardiac muscle cell action potential
Cadherin signaling
Glycosylphosphatidylinositol (GPI)−anchor biosynthesis
Nicotine addiction
Retrograde endocannabinoid signaling
Mineral absorption

Figure EV5. Comparative transcriptomic analysis between indicated conditions.

(**A**) Heatmap, GO, and KEGG enrichments of differentially expressed transcripts in CFAE + 8 + IR2 versus CFAE + 8 + IR1. Enriched GO-BP and KEGG pathways are displayed with font size scaled to significance. (**B**) Venn diagrams show the common down- or up-regulated genes between CFAE + 8 + IR1 versus C6FAE and CFAE + 8 + IR2 versus C6FAE. (**C**) Heatmap, GO, and KEGG enrichments of transcripts in the intersection of comparisons. Enriched GO-BP terms and KEGG pathways for each cluster are displayed, with font size scaled to enrichment significance.

 © The Author(s)
