# Supplementary material for: IRAK4 constrains cellular plasticity during chemically-induced cell fate reprogramming into multiple lineages
Source: EMBO Rep. 2026 Jul 15;27(16):4903–34. doi: 10.1038/s44319-026-00855-9 (PMC13503836; doi:10.1038/s44319-026-00855-9)
Supplement: Supplementary file 2 [file 44319_2026_855_MOESM1_ESM.pdf]

Appendix Table of Contents

Appendix Figure S1………………………………. 2

Appendix Figure S2………………………………. 3

Appendix Figure S3………………………………. 4

Appendix Figure S4………………………………. 5

Appendix Figure S5………………………………. 6

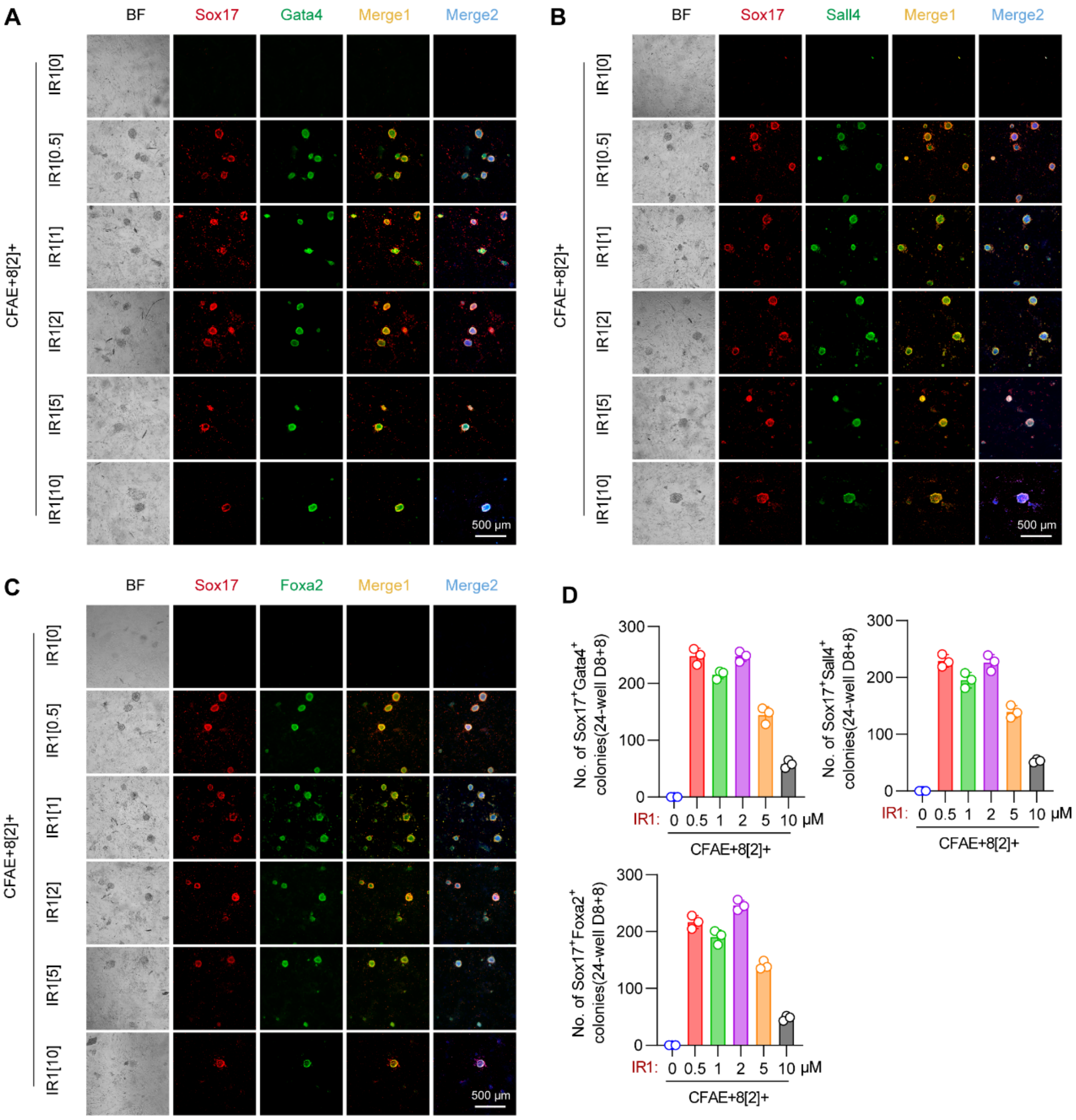

Appendix Figure S1. Optimal concentration determination of IR1 during MEF-XEN reprogramming. Related to Figure 2. (A)-(C) Immunofluorescence staining of XEN master regulators Sox17, Gata4, Sall4, and Foxa2 under CFAE+8 medium with a series of doses of IR1 induction for 16 days, respectively. Scale bar: 500 μm. (D) The number of $Sox17^{+}Gata4^{+}$, $Sox17^{+}Sall4^{+}$, and $Sox17^{+}Foxa2^{+}$ colonies in (A)-(C). Data are represented as mean ± SD. n=3 biological replicates, respectively.

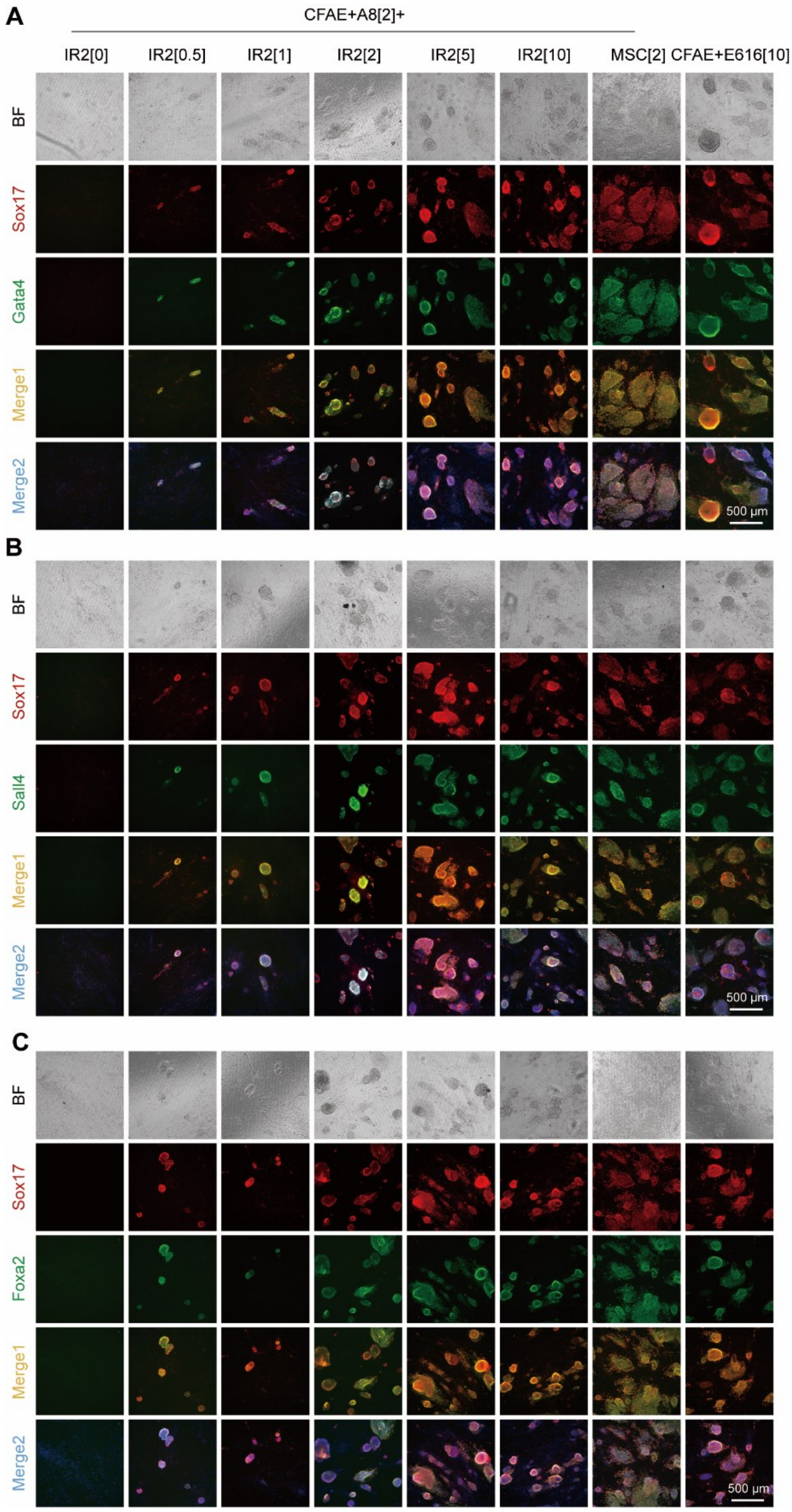

Appendix Figure S2. Optimal concentration determination of IR2 during MEF-XEN reprogramming. Individual raw images and merged panel related to **Figure 2A**. (A)-(C) Immunofluorescence staining of XEN master regulators Sox17, Gata4, Sall4, and Foxa2 under CFAE+8 medium with a series of doses of IR2 induction for 12 days, respectively. Scale bar: 500 µm.

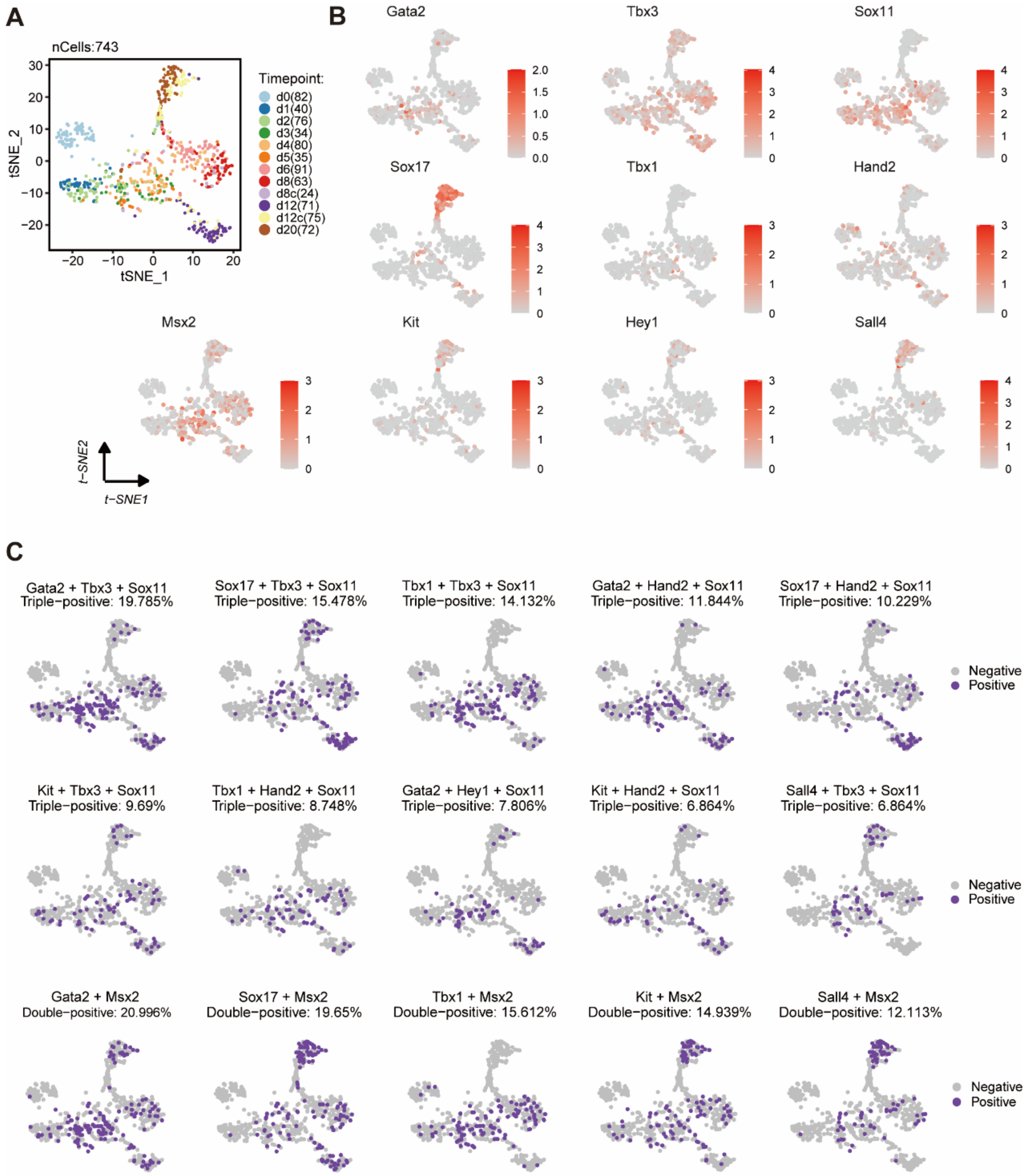

Appendix Figure S3 (**A**) t-SNE visualization of single cells from the MEF-to-XEN reprogramming time course. Cells were collected at indicated time points (d0 to d20) and analyzed by Smart-seq2. (**B**) Expression patterns of representative marker genes for the three germ layers. (**C**) Co-expression patterns of the top-ranked gene combinations.

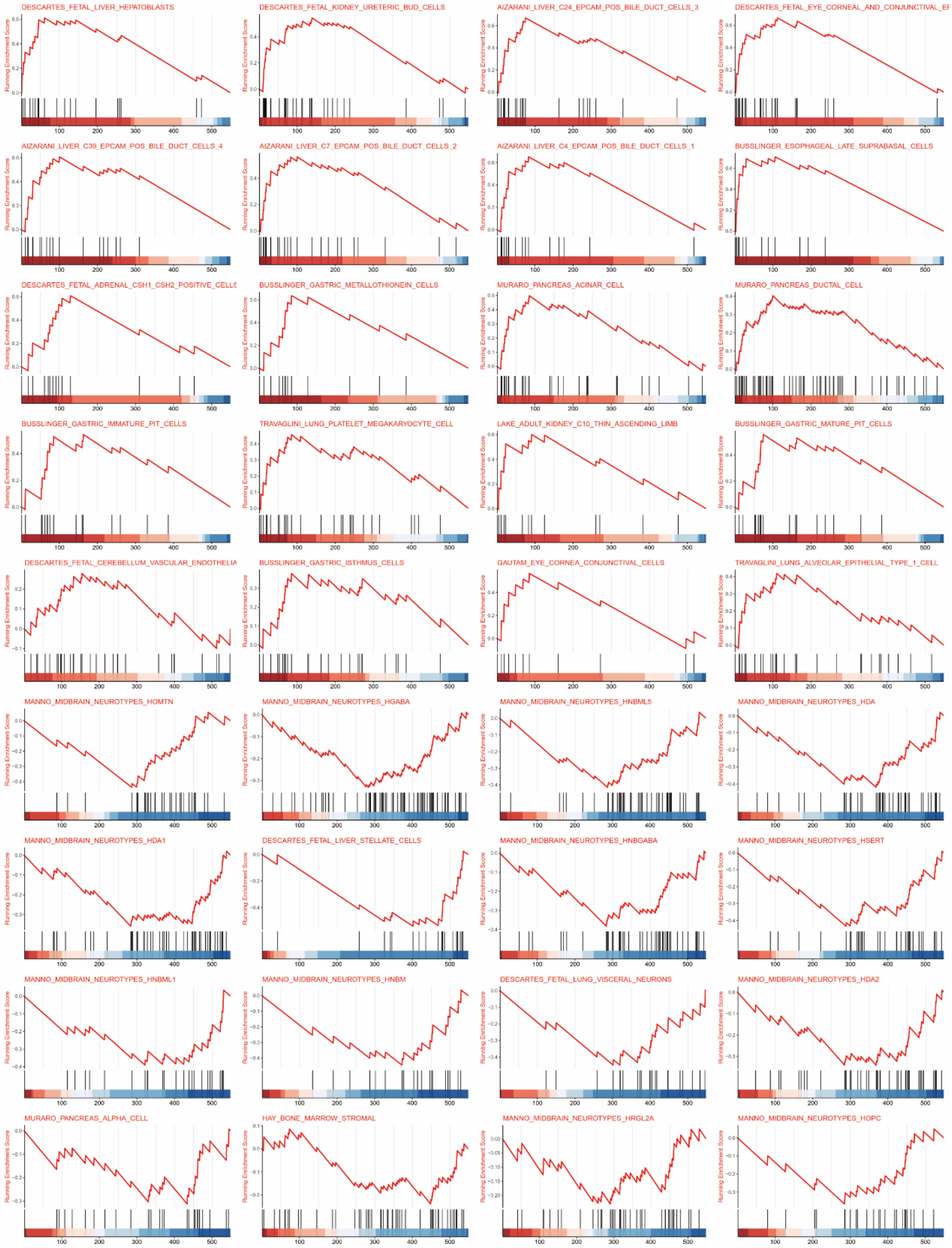

Appendix Figure S4 GSEA enrichments of multi-lineage cell fates in shIrak4 versus scramble.

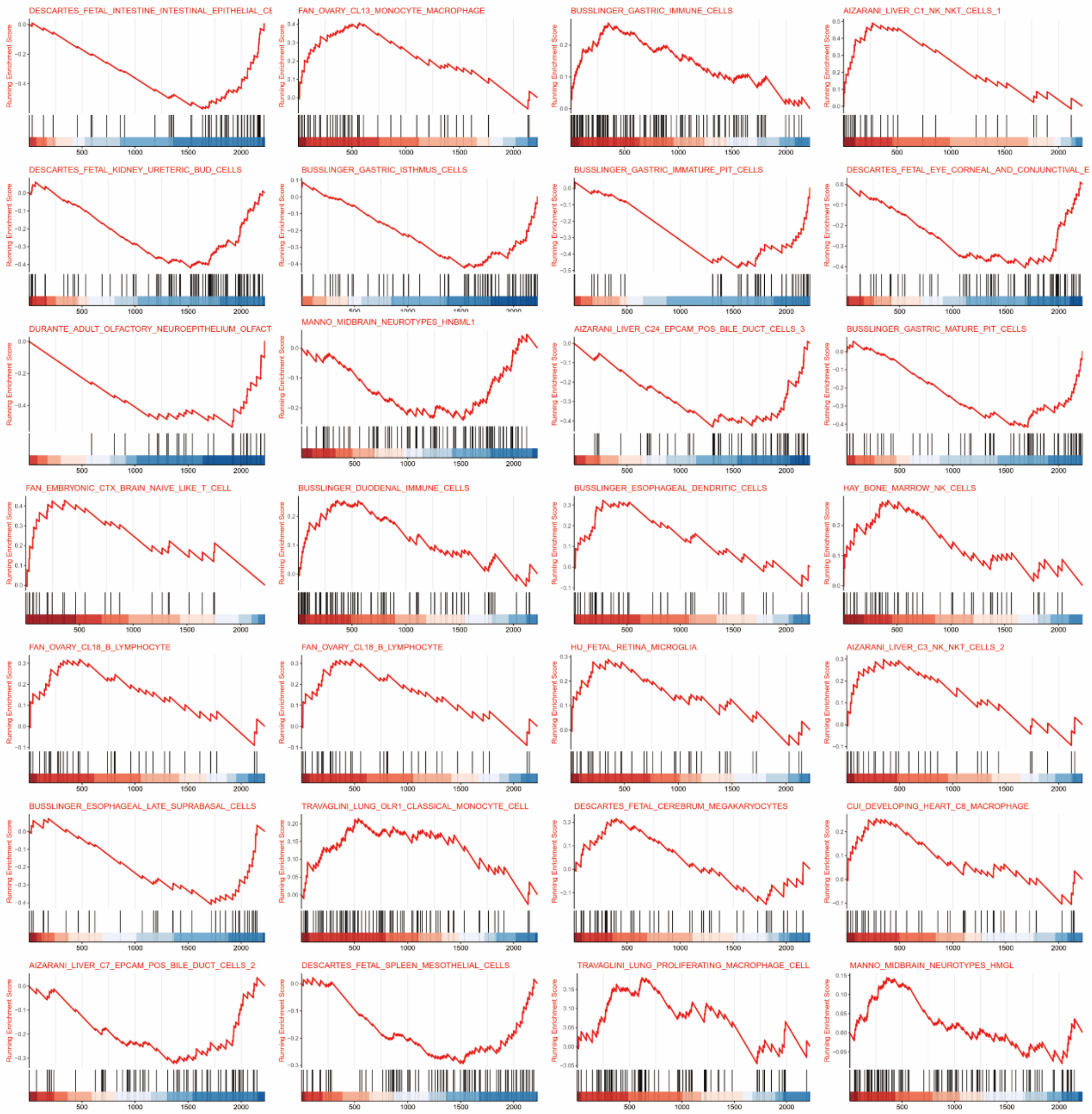

Appendix Figure S5 GSEA enrichments of multi-lineage cell fates in *Tetone-Irak4* versus *vector*.
