# Supplementary material for: IRAK4 constrains cellular plasticity during chemically-induced cell fate reprogramming into multiple lineages
Source: EMBO Rep. 2026 Jul 15;27(16):4903–34. doi: 10.1038/s44319-026-00855-9 (PMC13503836; doi:10.1038/s44319-026-00855-9)
Supplement: Supplementary file 3 [file 44319_2026_855_MOESM2_ESM.pdf]

EMBO reports

IRAK4 constrains cellular plasticity during chemically-induced cell fate reprogramming into multiple lineages

Chuanshu Huang, Xiaoyun Han, Tao Wang, Yang Zhao, and Jun Li

Corresponding author(s): Yang Zhao (yangzhao@pku.edu.cn), Chuanshu Huang (huangchuanshu@ojlab.AC.cn), Jun Li (jun.li@wmu.edu.cn).

Review Timeline:

Submission Date:	11th Nov 25
Editorial Decision:	13th Jan 26
Revision Received:	30th Apr 26
Editorial Decision:	8th Jun 26
Revision Received:	14th Jun 26
Accepted:	19th Jun 26

Editor: Esther Schnapp

Transaction Report:

(Note: With the exception of the correction of typographical or spelling errors that could be a source of ambiguity, letters and reports are not edited. Depending on transfer agreements, referee reports obtained elsewhere may or may not be included in this compilation. Referee reports are anonymous unless the Referee chooses to sign their reports.)

Dear Dr. Li,

Thank you for the submission of your manuscript to EMBO reports. We have now received the comments from 2 referees, which are pasted below. Referee 2 unfortunately never sent a report, despite several reminders, but we can proceed with the 2 reports we have.

As you will see, both referees acknowledge that the findings are potentially interesting. However, they also have several suggestions for how the study should be improved and strengthened. I think all suggestions from referee 2 should be addressed. Referee 1 point 1 might not have to be addressed, but if you have more data on this point, please do add it. Referee 1 point 3 might also be difficult to address but if you have an idea please provide more data. Referee 1 point 4 could be addressed in the ms text, and all following points from this referee must be addressed. Please let me know if you have any questions or comments regarding the revisions, and we can discuss this further, also in a video chat, i you like.

I would thus like to invite you to revise your manuscript with the understanding that the referee concerns must be fully addressed and their suggestions taken on board. Please address all referee concerns in a complete point-by-point response. Acceptance of the manuscript will depend on a positive outcome of a second round of review. It is EMBO reports policy to allow a single round of major revision only and acceptance or rejection of the manuscript will therefore depend on the completeness of your responses included in the next, final version of the manuscript.

We realize that it is difficult to revise to a specific deadline. In the interest of protecting the conceptual advance provided by the work, we recommend a revision within 3 months (15th Apr 2026). Please discuss the revision progress ahead of this time with the editor if you require more time to complete the revisions.

IMPORTANT NOTE: we perform an initial quality control of all revised manuscripts before re-review. Your manuscript will FAIL this control and the handling will be DELAYED if the following APPLIES:
1) A data availability section providing access to data deposited in public databases is missing. If you have not deposited any data, please add a sentence to the data availability section that explains that.
2) Your manuscript contains statistics and error bars based on n=2. Please use scatter blots in these cases. No statistics should be calculated if n=2.

When submitting your revised manuscript, please review the submission guidelines (https://link.springer.com/journal/44319/submission-guidelines#cms-Revised-submissions) and carefully review the instructions that follow below. Failure to include requested items will delay the evaluation of your revision.

1) a .docx formatted version of the manuscript text (including legends for main figures, EV figures and tables). Please make sure that the changes are highlighted to be clearly visible.

2) individual production quality figure files as .eps, .tif, .jpg (one file per figure). See hhttps://media.springernature.com/original/springer-cms/rest/v1/content/27825798/data/v1 for more info on how to prepare your figures.

3) We replaced Supplementary Information with Expanded View (EV) Figures and Tables that are collapsible/expandable online. A maximum of 5 EV Figures can be typeset. EV Figures should be cited as 'Figure EV1, Figure EV2" etc... in the text and their respective legends should be included in the main text after the legends of regular figures.

- For the figures that you do NOT wish to display as Expanded View figures, they should be bundled together with their legends in a single PDF file called *Appendix*, which should start with a short Table of Content. Appendix figures should be referred to in the main text as: "Appendix Figure S1, Appendix Figure S2" etc. See 'Expanded View' section of the submission guidelines.

- Additional Tables/Datasets should be labeled and referred to as Table EV1, Dataset EV1, etc. Legends have to be provided in a separate tab in case of .xls files. Alternatively, the legend can be supplied as a separate text file (README) and zipped together with the Table/Dataset file.

4) a .docx formatted letter INCLUDING the reviewers' reports and your detailed point-by-point responses to their comments. As part of the EMBO Press transparent editorial process, the point-by-point response is part of the Review Process File (RPF), which will be published alongside your paper.

5) a complete author checklist, which you can download from our author guidelines. Please insert information in the checklist that is also reflected in the manuscript. The completed author checklist will also be part of the RPF.

6) Please note that all corresponding authors are required to supply an ORCID ID for their name upon submission of a revised manuscript (<https://orcid.org/>), which can be linked to your profile in the manuscript submission system.

7) Before submitting your revision, primary datasets produced in this study need to be deposited in an appropriate public database. Please remember to provide a reviewer password if the datasets are not yet public. The accession numbers and database should be listed in a formal "Data Availability" section placed after Methods. Please note that the Data Availability Section is restricted to new primary data that are part of this study. * Note - All links should resolve to a page where the data can be accessed. *

If your study has not produced novel datasets, please mention this fact in the Data Availability Section.

8) At EMBO Press we ask authors to provide source data for the main manuscript figures. You will receive a separate email with instructions for providing source data with your revised manuscript, including how to upload and organize the files.

9) Our journal also encourages inclusion of *data citations in the reference list* to directly cite datasets that were re-used and obtained from public databases. Data citations in the article text are distinct from normal bibliographical citations and should directly link to the database records from which the data can be accessed. In the main text, data citations are formatted as follows: "Data ref: Smith et al, 2001" or "Data ref: NCBI Sequence Read Archive PRJNA342805, 2017". In the Reference list, data citations must be labeled with "[DATASET]". A data reference must provide the database name, accession number/identifiers and a resolvable link to the landing page from which the data can be accessed at the end of the reference.

10) Regarding data quantification, the following points must be specified in each figure legend:

- the name of the statistical test used to generate error bars and P values,

- the number (n) of independent experiments (please specify technical or biological replicates) underlying each data point,

- the nature of the bars and error bars (s.d., s.e.m.),

- If the data are obtained from n Program fragment delivered error ``Can't locate object method "less" via package "than" (perhaps you forgot to load "than"?) at //ejpvfs23/sites23b/embor_www/letters/embor_decision_revise_and_review.txt line 54.' 2, use scatter blots showing the individual data points.

Discussion of statistical methodology can be reported in the materials and methods section, but figure legends should contain a basic description of n, P and the test applied.

- Please also include scale bars in all microscopy images.

11) The journal requires a statement specifying whether or not authors have competing interests (defined as all potential or actual interests that could be perceived to influence the presentation or interpretation of an article). In case of competing interests, this must be specified in your disclosure statement.

12) All Materials and Methods need to be described in the main text using our 'Structured Methods' format, which is required for all research articles. According to this format, the Methods section includes a separate Reagents and Tools Table file (listing key reagents, experimental models, software and relevant equipment and including their sources and relevant identifiers) and a Methods and Protocols section describing the methods using a step-by-step protocol format. The aim is to facilitate adoption of the methodologies across labs. More information on how to adhere to this format as well as a downloadable template (.docx) for the Reagents and Tools Table can be found in our author guidelines.

We would also welcome the submission of cover suggestions, or motifs to be used by our Graphics Illustrator in designing a cover.

As part of the EMBO publication's Transparent Editorial Process, EMBO reports publishes online a Review Process File (RPF) to accompany accepted manuscripts. This File will be published in conjunction with your paper and will include the referee reports, your point-by-point response and all pertinent correspondence relating to the manuscript.

You are able to opt out of this by letting the editorial office know (emboreports@embo.org). If you do opt out, the Review Process File link will point to the following statement: "No Review Process File is available with this article, as the authors have chosen not to make the review process public in this case."

I look forward to seeing a revised form of your manuscript when it is ready.

Kind regards,
Esther

Esther Schnapp, PhD
Senior Editor
EMBO reports

Referee #1:

This manuscript identifies IRAK4 as a previously unrecognized barrier to chemical reprogramming and investigates its role during the MEF→CaMP→XEN-like transition as well as MEF→iHep transdifferentiation. The overall concept is interesting, the dataset is sizeable, and the work has potential significance.
However, several key conclusions lack sufficient mechanistic support, the causal links between observations remain incomplete, and certain data presentations require clarification or strengthening. With additional mechanistic evidence and improved analytical rigor, the manuscript could reach a publishable standard.
Major Concerns
1. Insufficient mechanistic evidence for how cytoplasmic IRAK4 alters cell-cycle regulation
IRAK4 is a cytosolic innate immune kinase, yet the manuscript claims that its inhibition remodels cell-cycle checkpoints. This conclusion currently relies only on GO/GSEA enrichment and FACS profiles (reduced G0/G1 and increased G2/M), without providing direct molecular evidence.
Stronger mechanistic data-such as changes in CDK activity, checkpoint kinase phosphorylation, DNA-damage signaling, or mitotic markers-are required to support a direct causal relationship between IRAK4 inhibition and cell-cycle control.
2. Missing evidence that direct manipulation of the cell cycle promotes reprogramming
To argue that "IRAK4 promotes reprogramming through cell-cycle remodeling," one must show that independent cell-cycle perturbation (e.g., G1 shortening or G2/M extension) also enhances reprogramming efficiency.
Without such functional validation, the mechanistic chain remains incomplete.
3. Lack of causal explanation for how IRAK4 inhibition drives CaMP and XEN-like conversion
RNA-seq analysis shows activation of XEN markers (Sox17, Gata4, Sall4), but the mechanism by which IRAK4 inhibition initiates such lineage-specific programs is not explored.
For example, it remains unclear whether this occurs via NF-κB suppression, chromatin accessibility changes, enhancer activation, or alternative pathways.
4. Insufficient evidence that IRAK4 is a "shared" barrier for multiple reprogramming paradigms
The manuscript proposes IRAK4 as a common barrier for CiPSC induction and MEF→iHep transdifferentiation.
However, only functional hepatocyte readouts (Alb^{+}/$Cyp3a11^{+}$) are presented for the iHep context, without mechanistic parallels.
Key questions remain unanswered:
• Does IRAK4 inhibition similarly alter cell-cycle profiles in MEF→iHep?
• Does it induce a comparable "lineage-priming" state?
• Does it suppress NF-κB target genes in this context?
Without such evidence, the conclusion that "IRAK4 is a pleiotropic barrier to cellular plasticity" is premature.
5. Quantification concerns in Figure 1B-D
The number of positive colonies in the IR2-treated group is dramatically higher than in the CFAE6-treated group, yet Sox17, Gata4, Sall4, and Foxa2 expression levels appear similar between them.
This raises a methodological question:
• Were IF images taken after normalizing cell numbers across groups?
• Is colony quantification accurate and based on standardized criteria?
Clarification is needed to ensure reliability.
6. CFAE+6 should be explicitly indicated as a positive control
Given the known difference in reprogramming efficiencies between CFAE+8 and CFAE+6, it is important to clearly label CFAE+6 as a positive control within the main text.
7. Inconsistent figure-spacing format
Figure numbering and spacing (notably on pages 93, 100, and 101) are inconsistent.
The manuscript should be carefully rechecked for uniform formatting.
8. GO interpretation appears selective and insufficiently justified
Although G1/S transition appears in the GO terms, it is not the most enriched category.
Why was this term chosen for further mechanistic exploration over more enriched terms such as extracellular matrix organization or collagen metabolic processes?
A clearer rationale is needed to avoid the impression of biased interpretation.
9. IRAK4 overexpression induces immune genes-cause or by-product?
Activation of immune-related genes under IRAK4 overexpression may reflect a nonspecific shift in cellular state rather than mechanistic relevance to reprogramming.
The manuscript implicitly assumes that IRAK4 acts through the same pathways used in innate immunity, but this might represent a "moonlighting" activity rather than a mechanistic necessity.
A more cautious interpretation is advised.
10. CaMP-state interpretation may overstate functional plasticity

RNA-seq reveals induction of genes from diverse lineages (neural, renal, pancreatic, etc.) following IRAK4 inhibition.
The authors interpret this as entry into a multipotent "CaMP" state.
However, this could alternatively reflect:
• transcriptional noise,
• identity destabilization,
• or transient mixed-lineage expression preceding fate acquisition.
Without functional differentiation assays (e.g., directed induction toward neural or renal lineages), the claim of genuine multipotent priming remains speculative.
The conclusions should be balanced accordingly.
Minor Concerns
11. Figure fonts require unification
Figures-particularly Figures 3, 5, 6, and 7-display inconsistent font types and sizes, giving a disorganized appearance. Standardization is needed.
12. Volcano plot gene labels unclear
In Figures 5F and 6I, gene annotations in the volcano plots are difficult to read.
Other figures should also be carefully reviewed to ensure clarity.
13. Comparison with existing reprogramming enhancers
It would be useful to discuss whether IRAK4 inhibition provides higher or comparable reprogramming efficiency relative to previously published small-molecule enhancers.
This would help highlight the innovation and practical relevance of the findings.
14. Nomenclature issue: "shIrak4" used as a substitute noun
The term "shIrak4" is used as a stand-alone noun in several places, which is unconventional and confusing.
A more standard phrasing (e.g., "IRAK4 knockdown cells") should be adopted throughout.
15. English writing issues
Representative examples:
• "have the potential to initiate MEF-XEN reprogramming" is awkward and overly tentative.
• "moderately higher vs slightly elevated" is vague; quantitative fold-change or p-values should be provided instead of subjective descriptors.
A thorough language revision is recommended.
16. The Discussion section feels rather lengthy and could be more concise. I suggest reducing redundancy and improving clarity.

Summary Recommendation
The manuscript contains substantial data and addresses an interesting direction.
However, several core conclusions-particularly those linking IRAK4 inhibition to cell-cycle remodeling and enhanced plasticity-require stronger mechanistic support.
Once key experiments are added and major conceptual claims are stated more cautiously, the work could become significantly more compelling and suitable for publication.

Referee #3:

In this study, Li and colleagues attempted to show that the innate immune kinase IRAK4 functions as a previously unappreciated barrier to somatic cell plasticity and chemical reprogramming. Using pharmacological or genetic inhibition of IRAK4, the authors report enhanced multi lineage reprogramming of MEFs through CaMP and XEN like intermediate states, accompanied by increased colony formation and robust activation of core XEN transcriptional regulators. Mechanistically, they propose that IRAK4 suppression reshapes cell cycle dynamics to promote transcriptional plasticity. Importantly, the study further suggests that IRAK4 inhibition broadly facilitates cell fate conversion by significantly improving direct reprogramming of fibroblasts into functional hepatocyte-like cells. Overall, the manuscript is clearly written and logically organized, and most conclusions are supported by the presented data. However, a major limitation of the study is the lack of a clear mechanistic explanation for how IRAK4 inhibition erases somatic identity and facilitates acquisition of XEN or hepatocyte gene signatures.

Major comments:
1. The immunostaining images presented in Figures 1 and S1 are of very low quality. Staining for nuclear transcription factors such as SALL4, SOX17, or GATA4 should exhibit clear nuclear localization, which is not evident in these images. It is unclear whether this reflects image compression during the journal conversion process or suboptimal staining procedures.
2. In Figures 1b-d, the authors show that the number of double positive colonies (SOX17 plus an additional XEN marker) is substantially higher in the CFAE+8+IR2 condition compared to CFAE+6. However, in Figures 1e-h, expression levels of all tested XEN genes are markedly higher in CFAE+6. How can these observations be reconciled? Does the CFAE+6 condition induce stronger XEN gene expression per colony, whereas CFAE+8+IR2 increases colony number with low XEN gene expresion? What biological conclusions can be drawn from this discrepancy?
3. The authors state that "both treatments activated genes associated with cell fate specification and markers representative of

all three germ layers" (Figure 3F). However, little to no expression of endoderm markers is detectable in CFAE+8+IR1 or CFAE+6 conditions in the corresponding figure. This statement should be revised or better supported.
4. Clear transcriptional differences exist between CFAE+8+IR1, CFAE+8+IR2, and CFAE+6, including in core XEN markers. For example, Foxa2 is not upregulated in CFAE+6 (Figure 3I). The authors are encouraged to perform a deeper comparative analysis highlighting all major transcriptional differences between these three conditions, beyond GO term enrichment (Figures 4c-d), and to propose potential explanations for these differences.
5. The authors show that IRAK4 and IRAK1 expression is not detected in CFAE+6. To determine whether the effects of IR1 and IR2 are indeed mediated specifically through IRAK4/1 inhibition, the authors should test CFAE+6 conditions with and without IR1 or IR2 and quantify colony numbers. An increase in colonies upon IR1 or IR2 addition would suggest IRAK independent effects of these inhibitors.
6. Although CFAE+8+IR1, CFAE+8+IR2, and CFAE+6 slightly reduce the G0/G1 phase relative to CFAE+8, the reviewer is not convinced that this modest change in cell cycle dynamics is the key determinant enabling XEN induction. The major shift in cell cycle distribution already occurs with CFAE+8 alone. Therefore, the abstract statement that "IRAK4 inhibition remodels cell cycle dynamics, shortening G0/G1 and lengthening G2/M phases to license transcriptional plasticity" is not sufficiently supported. To strengthen this claim, the authors should test whether accelerating the cell cycle under CFAE+8 conditions alone (for example using p53 knockout MEFs) is sufficient to induce a XEN like transcriptional program. Such experiments would directly assess whether cell cycle acceleration per se accounts for the IR1/IR2-mediated induction of XEN gene expression.
7. As noted above, immunostaining in Figures 5a-c is not convincing due to the lack of clear nuclear localization of the indicated transcription factors.
8. In Figure 5E, the two independent shRNAs targeting IRAK4 induce non-consistent activation of XEN markers. While both shRNAs downregulate IRAK4 to a similar extent, SOX17 and SALL4 are much more strongly induced by shRNA-1, whereas GATA4 is preferentially induced by shRNA-2. The authors should address and explain this inconsistency.
9. In Figure 6, IRAK4 overexpression blocks MEF to XEN conversion, but the authors do not describe the effects of IRAK4 overexpression on cell cycle progression. Do these cells stop proliferating? If so, impaired proliferation could readily explain the failure of reprogramming. The reviewer suggests overexpressing IRAK4 in p53-deficient fibroblasts and re-testing reprogramming efficiency.
10. For hepatocyte reprogramming, it is unclear whether the PCR assays detecting Hnf4a and Foxa3 measure endogenous or exogenous transcripts. Demonstration of endogenous activation is essential to support successful reprogramming.
11. The immunostaining images in Figure 7 are of low quality and difficult to interpret. The reviewer is not convinced by the reported hepatocyte like morphology.
12. Do IR1 and IR2 similarly accelerate the cell cycle during hepatocyte conversion? Additionally, what explains the differences in hepatocyte reprogramming efficiency between IR1 and IR2?
13. The authors should assess expression of fibroblast identity markers following IRAK4 inhibition (for example Thy1, Postn, Col1a1, Col5a2) to directly demonstrate erasure of somatic identity.

Minor comments
1. Line 191: "teems" should be corrected to "terms".
2. The statement that "erasure of somatic identity and initiation of the early reprogramming phase represent the principal rate limiting steps" is inaccurate. Early reprogramming and somatic identity loss occur efficiently in most cells, whereas activation of endogenous late pluripotency or lineage defining genes is generally rate limiting. This statement should be revised.

Dear Esther Schnapp,

We sincerely thank you and reviewers for their time and insightful comments, which have led to substantial improvements in our manuscript. All concerns raised have been carefully addressed, and we hope you will agree that the revised version is now suitable for publication in ***EMBO Reports***. We have also re-evaluated the contributions of all authors and, with prior approval from the editorial office, updated the author order in the revised manuscript accordingly. We are deeply grateful for your guidance throughout this process.

Sincerely,
Jun Li, Ph.D. (Lead Contact)

Reviewers' comments:

Referee #1:

This manuscript identifies IRAK4 as a previously unrecognized barrier to chemical reprogramming and investigates its role during the MEF→CaMP→XEN-like transition as well as MEF→iHep transdifferentiation. The overall concept is interesting, the dataset is sizeable, and the work has potential significance.

However, several key conclusions lack sufficient mechanistic support, the causal links between observations remain incomplete, and certain data presentations require clarification or strengthening. With additional mechanistic evidence and improved analytical rigor, the manuscript could reach a publishable standard.

Major Concerns

1. Insufficient mechanistic evidence for how cytoplasmic IRAK4 alters cell-cycle regulation IRAK4 is a cytosolic innate immune kinase, yet the manuscript claims that its inhibition remodels cell-cycle checkpoints. This conclusion currently relies only on GO/GSEA enrichment and FACS profiles (reduced G0/G1 and increased G2/M), without providing direct molecular evidence. Stronger mechanistic data-such as changes in CDK activity, checkpoint kinase phosphorylation, DNA-damage signaling, or mitotic markers-are required to support a direct causal relationship between IRAK4 inhibition and cell-cycle control.

Response: While the editor indicated that this concern does not have to be addressed, we thank the reviewer for this insightful suggestion. We have now provided additional data to clarify this point. Beyond its canonical cytoplasmic residence, IRAK4 is also capable of

entering the nucleus to mediate nuclear-specific functions[1,2]. After considering all reviewers' constructive suggestions and our experimental results, we conclude that IRAK4i promotes cellular reprogramming through multiple molecular mechanisms, including accelerating the cell cycle and inhibiting the NF-κB pathway. Nevertheless, altering these two factors individually in the CFAE+8 background alone is insufficient to mimic the effects of IRAK4i. Instead, enhanced chromatin accessibility, which enables the establishment of multiple lineages, may play a more crucial role. (**Response Figure 1-3**)

2. Missing evidence that direct manipulation of the cell cycle promotes reprogramming. To argue that "IRAK4 promotes reprogramming through cell-cycle remodeling," one must show that independent cell-cycle perturbation (e.g., G1 shortening or G2/M extension) also enhances reprogramming efficiency. Without such functional validation, the mechanistic chain remains incomplete.

Response: We thank the reviewer for this insightful and constructive suggestion. Indeed, multiple studies have demonstrated that suppression or loss of TP53 facilitates somatic cell reprogramming toward induced pluripotent stem cells (iPSCs)[3-6] under OSKM inducible system. Based on this rationale, we initially planned to test whether accelerating the cell cycle under CFAE+8 conditions alone, for example using TP53 knockout MEFs (suggested by Referee #3), would be sufficient to induce a XEN-like transcriptional program. However, after consulting multiple mouse vendors and other alternative strategies, we found that homozygous TP53 knockout mice frequently exhibit severe developmental defects, compromised viability, or embryonic lethality, making it extremely difficult to reliably obtain suitable embryos for MEF derivation. Consequently, we were unable to carry out this experiment as originally proposed. Alternatively, we employed shRNA-mediated knockdown of Trp53 (shTrp53) to establish Trp53-deficient conditions. However, Trp53 depletion alone failed to induce XEN-like colony formation. Unlike the OSKM system, which alone can induce pluripotent conversion, the CFAE+8 system alone cannot. Therefore, these two systems are distinct, and it seems reasonable that Trp53 knockdown in these systems does not yield similar results, which was inconsistent with the indispensable roles of Trp53 in human CiPSC induction[7].

As an alternative approach, we investigated cell cycle modulation using small-molecule inhibitors AT9283 (an Aurora kinase A/B inhibitor) and Barasertib-HQPA (a selective Aurora kinase B inhibitor) under CFAE+8+IR2 conditions. Notably, treatment with either inhibitor completely abolished the formation of XEN-like colonies, indicating that proper cell cycle progression is indeed an important factor supporting reprogramming (**Response Figure 1**). However, since this approach only demonstrates that disruption of the cell cycle impairs reprogramming, rather than proving that cell cycle acceleration is sufficient to induce XEN-like fate under CFAE+8 conditions, we agree with the reviewer that cell cycle remodeling alone cannot fully account for IR1/IR2-mediated effects. According to next suggestion of the reviewer, we investigated the chromatin landscape and found that

enhanced chromatin accessibility underlies the mechanism by which IR2 promotes cell reprogramming (**Response Figure 2**).

Therefore, we have revised the manuscript to moderate our conclusion and clarify that cell cycle regulation represents an important, but not exclusive, contributor to the acquisition of XEN-like identity (page 9 lines 343-349).

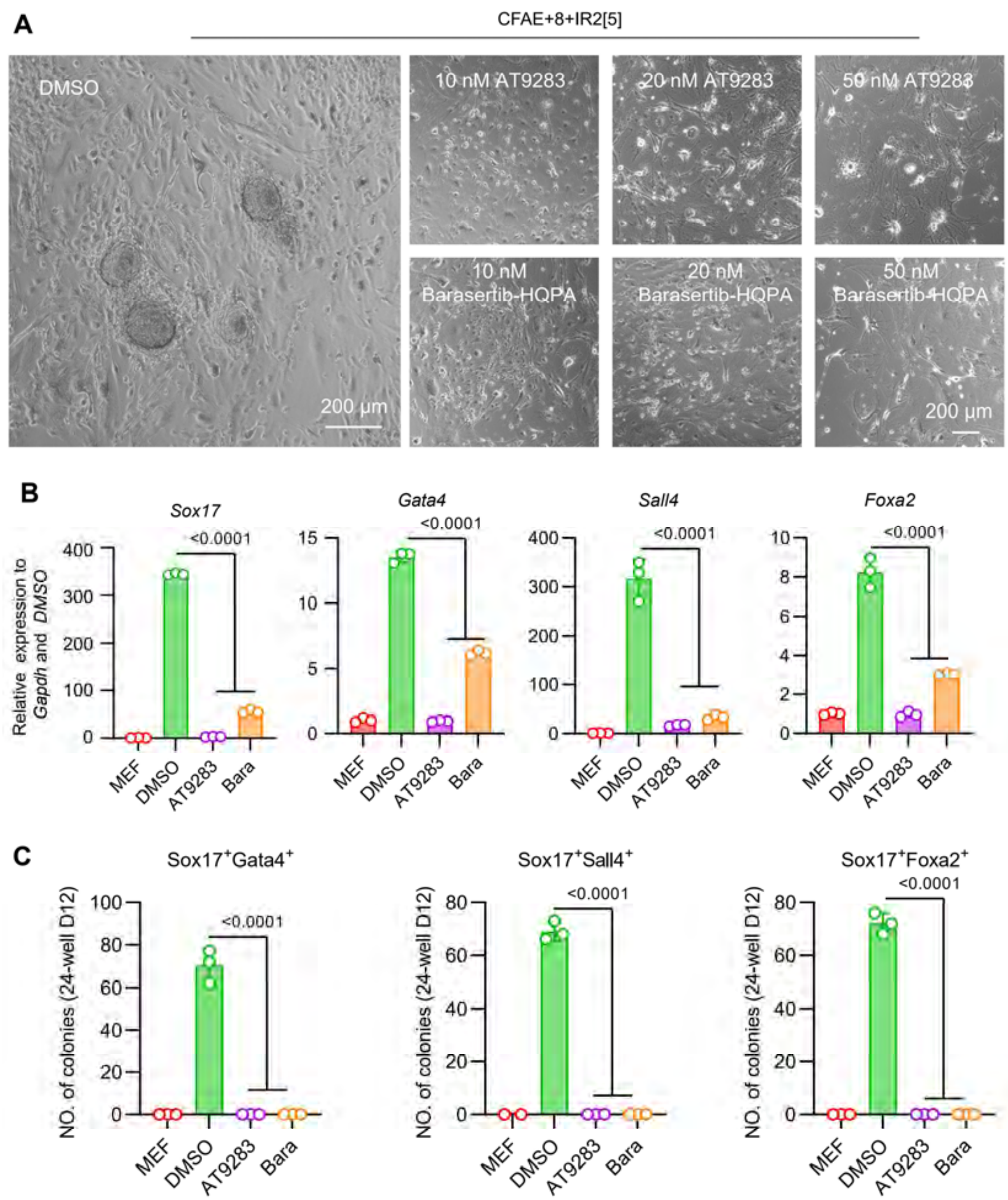

Response Figure 1 Disruption of the cell cycle impairs cell reprogramming
(**A**) Morphology of colonies or cells induced by indicated cocktails in bright field. Scale bar, 200 μm. (**B**) Relative mRNA expression of *Sox17, Gata4, Sall4,* and *Foxa2* in indicated samples in MEF or CFAE+8+IR2 basal medium for 12 days, respectively. Bara:

Barasertib-HQPA. (**C**) Percentages of $Sox17^{+}Gata4^{+}$, $Sox17^{+}Sall4^{+}$, and $Sox17^{+}Foxa2^{+}$ colonies.

3. Lack of causal explanation for how IRAK4 inhibition drives CaMP and XEN-like conversion RNA-seq analysis shows activation of XEN markers (Sox17, Gata4, Sall4), but the mechanism by which IRAK4 inhibition initiates such lineage-specific programs is not explored. For example, it remains unclear whether this occurs via NF-κB suppression, chromatin accessibility changes, enhancer activation, or alternative pathways.

Response: We appreciate the reviewer's insightful comment regarding the causal mechanism underlying IRAK4 inhibition-driven CaMP and XEN-like conversion. We acknowledge that while our RNA-seq analysis demonstrates activation of XEN markers (Sox17, Gata4, Sall4) upon IRAK4 inhibition, the specific molecular pathway linking IRAK4 suppression to lineage-specific program initiation remains unexplored in this study. The reviewer appropriately highlights several possible mechanisms (including NF-κB suppression, chromatin accessibility alterations, enhancer activation, or alternative pathways) that could mediate this phenotypic transition. To address this critical point, we have performed additional rescue experiments demonstrating that NF-κB inhibition contributes, at least in part, to this reprogramming process. Specifically, exogenous TNF-α treatment effectively blocked IRAK4 inhibitor-mediated cell fate conversion (**Response Figure 3**), suggesting that attenuation of NF-κB signaling is necessary for the observed XEN-like transition. Meanwhile, we investigated the chromatin landscape and found that enhanced chromatin accessibility underlies the mechanism by which IR2 promotes cell reprogramming (**Response Figure 2**).

In the revised manuscript, we have incorporated Response Figure 2 as main **Figure 5** and Response Figure 3 as main **Figure 7I**. Additionally, we have expanded the relevant section to discuss the possible mechanisms highlighted by the reviewer (page 5-6 lines190-211, page 9, lines 325-331 and page 7 lines 240-243). We believe the additional data and revised discussion provide appropriate mechanism for our findings. We thank the reviewer again for this valuable suggestion, which has significantly strengthened the scientific rigor of our study.

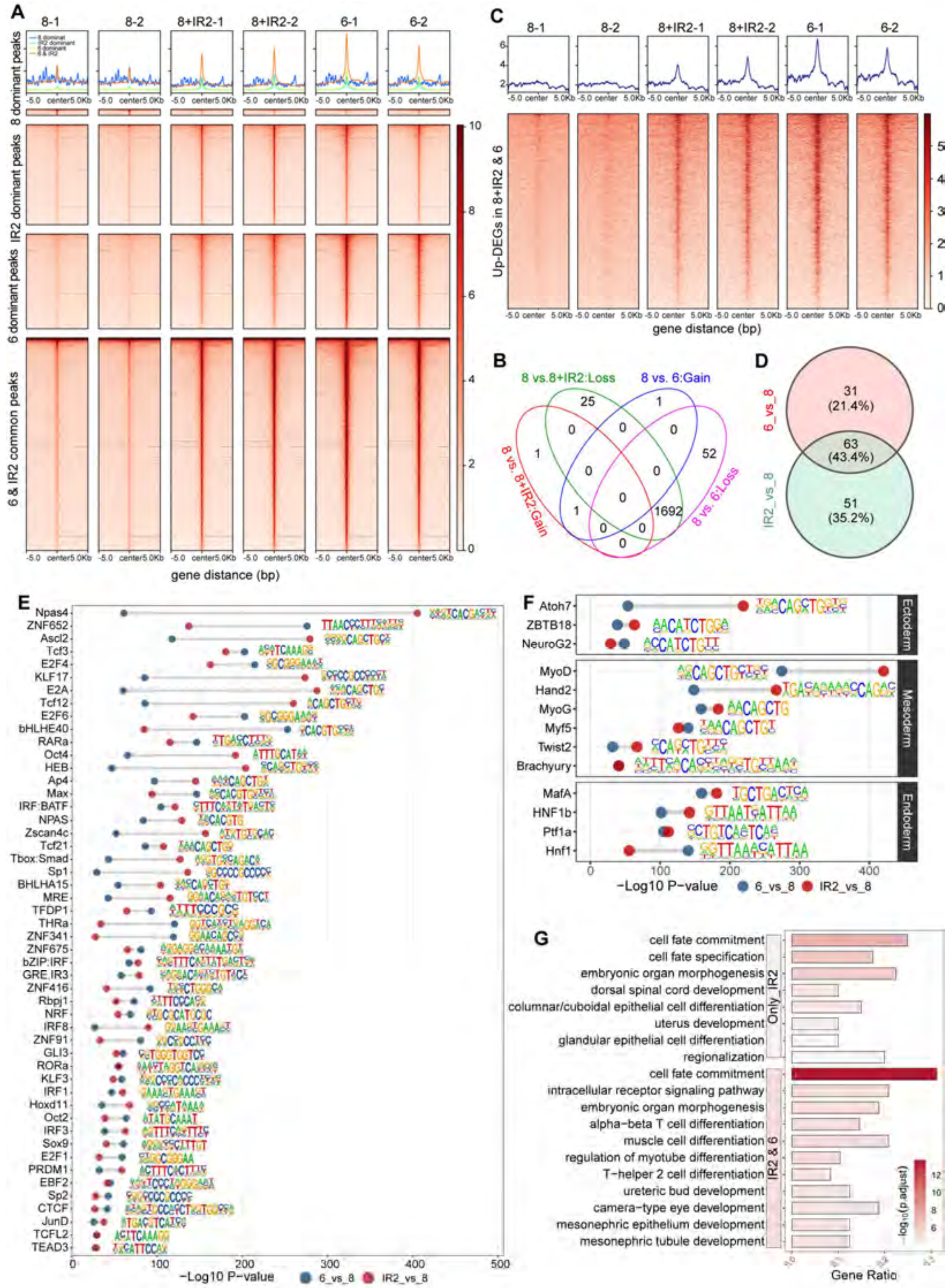

Response Figure 2 IRAK4 inhibition enhances chromatin accessibility and promotes cell fate transition.

(**A**) ATAC-seq analysis of chromatin accessibility near TSS in MEF medium, CFAE+6, CFAE+8, and CFAE+8+IR2 cultured cells for 8 days, respectively. -1 and -2 represent two replicates. (**B**) Venn diagram of peaks (loss and gain) from the union of the 8 vs. 6 and 8 vs. 8+IR2

comparisons. (**C**) PlotHeatmap of ATAC-seq data for up-regulated genes overlapped in 8+IR2 and 6. (**D**) Venn diagram of transcription factors enriched in ATAC-seq data of 6_vs_8 and 8+IR2_vs_8. (**E**)-(**F**) Enriched significant transcription factors: DNA motif pairs in the intersection of 6_vs_8 and 8+IR2_vs_8. Three germ layers related transcription factors: DNA motif pairs was showed in (**F**). (**G**) 63 TFs in (**D**) enriched GO terms.

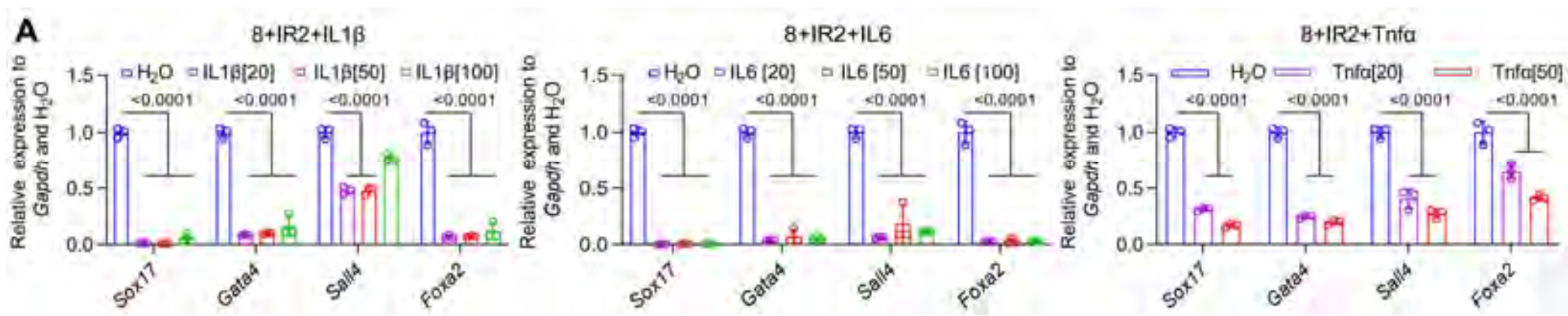

Response Figure 3 IL1β, IL-6, and TNFα disrupted XEN master regulators in CFAE+8+IR2. Numbers in brackets are the concentrations (ng/mL) of cytokines. Data are presented as mean ± SD.

4. Insufficient evidence that IRAK4 is a "shared" barrier for multiple reprogramming paradigms. The manuscript proposes IRAK4 as a common barrier for CiPSC induction and MEF→iHep transdifferentiation. However, only functional hepatocyte readouts (Alb^+/$Cyp3a11^+$) are presented for the iHep context, without mechanistic parallels. Key questions remain unanswered:

• Does IRAK4 inhibition similarly alter cell-cycle profiles in MEF→iHep?
• Does it induce a comparable "lineage-priming" state?
• Does it suppress NF-κB target genes in this context?

Without such evidence, the conclusion that "IRAK4 is a pleiotropic barrier to cellular plasticity" is premature.

Response: We are very grateful to the reviewer for this careful reading of our paper. Based on the editor's suggestion, we have now revised the text in the manuscript as follows:

- We have softened the conclusion from "underscoring its role as a pleiotropic barrier to cellular plasticity" to "underscoring its context-dependent role as a barrier to cell plasticity across multiple reprogramming contexts" (page 2, lines 77-78 and page 11, lines 410-414).

- We acknowledged that mechanistic parallels between the CiPSC and iHep systems remain to be fully established, emphasizing that the shared phenotype (enhanced reprogramming efficiency) across distinct systems supports IRAK4 as a context-dependent barrier, though underlying mechanisms may differ (page 11, lines 410-420). We also noted this as a limitation of the current study in the Discussion section (page 12, line 431).

We thank the reviewer again for raising this valuable and key question.

5. Quantification concerns in Figure 1B-D

The number of positive colonies in the IR2-treated group is dramatically higher than in the CFAE6-treated group, yet Sox17, Gata4, Sall4, and Foxa2 expression levels appear similar between them.

This raises a methodological question:

• Were IF images taken after normalizing cell numbers across groups?

• Is colony quantification accurate and based on standardized criteria?

Clarification is needed to ensure reliability.

Response: We thank the reviewer for the careful assessment of our manuscript and the valuable comment regarding the quantification in Figure 1B-D. Regarding the apparent discrepancy between colony number and marker expression in IR2-treated versus CFAE+6-treated groups, we would like to clarify:

1) For imaging standardization, all immunofluorescence images were captured under identical microscope magnification using scale bars as reference. Since all groups started with the same initial cell numbers and most colonies are multilayered structures where individual cells within colonies cannot be reliably visualized by microscopy, we did not perform cell number normalization within colonies. Therefore, all figures were raw images with the same scale bar without normalizing cell numbers across groups.

2) While the IR2-treated group indeed showed higher colony numbers, the colony sizes were smaller at the 2 μM concentration in Figure 1, which may have contributed to the reviewer's observation. After optimizing the concentration to 5 μM in Figure 2A, we found that colony sizes became comparable between groups. Colony quantification was conducted using consistent and standardized criteria across all groups, with all colonies counted based on double-positive immunofluorescence staining, ensuring the reliability and comparability of our quantitative data. In all groups, colonies exhibited a wide range of sizes from small to large, with both monolayer and multilayer morphologies observed. We both counted monolayer (over 20 cells) and multilayer colonies in Figure 1B-D.

6. CFAE+6 should be explicitly indicated as a positive control. Given the known difference in reprogramming efficiencies between CFAE+8 and CFAE+6, it is important to clearly label CFAE+6 as a positive control within the main text.

Response: We thank the reviewer for this helpful suggestion. In accordance with the comment, we have now explicitly indicated CFAE+6 as a positive control in the main text (page 3, line 91 and 98).

7. Inconsistent figure-spacing format. Figure numbering and spacing (notably on pages 93, 100, and 101) are inconsistent. The manuscript should be carefully rechecked for uniform formatting.

Response: We thank the reviewer for pointing out the formatting inconsistencies in the figure numbering and spacing. We have carefully rechecked the entire manuscript and have now ensured that all figures are numbered uniformly and that spacing between figures and text is consistent throughout. We apologize for any oversight and appreciate the reviewer's careful reading.

8. GO interpretation appears selective and insufficiently justified. Although G1/S transition appears in the GO terms, it is not the most enriched category. Why was this term chosen for further mechanistic exploration over more enriched terms such as extracellular matrix organization or collagen metabolic processes? A clearer rationale is needed to avoid the impression of biased interpretation.

Response: We thank the reviewer for this insightful comment regarding our selection of the G1/S transition term for further mechanistic exploration. Our rationale was primarily driven by experimental observations: we consistently noted that treated cells exhibited significantly accelerated proliferation, which naturally directed our attention toward cell-cycle-related pathways. Regarding the highly enriched terms such as extracellular matrix (ECM) organization and collagen metabolic processes, we fully agree that these represent prominent features among the downregulated genes. In cellular reprogramming, successful lineage conversion is known to involve an initial phase of fibroblast identity erasure, which entails extensive ECM remodeling, followed by the establishment of other cell fate. Accordingly, we interpret the enrichment of ECM-related terms as an early **indicator** of successful reprogramming initiation.

However, as the reviewer rightly points out, we cannot exclude the possibility that ECM remodeling may actively contribute to the establishment of alternative cell fates. While we currently lack a definitive experimental strategy to directly test this hypothesis, we believe this represents an important and promising avenue for future investigation, particularly given that analogous regulatory roles of the ECM have been documented in other cell reprogramming systems[8]. We haved accordingly expanded our Discussion (page 10, lines 355-375) to acknowledge this possibility and clarify the potential functional significance of ECM dynamics beyond its role as an early reprogramming marker.

By incorporating these considerations, we aim to provide a more balanced rationale for our focus on cell-cycle mechanisms while avoiding any impression of selective interpretation. We apologize for this oversight and thank the reviewer again for the kind reminder.

9. IRAK4 overexpression induces immune genes-cause or by-product? Activation of immune-related genes under IRAK4 overexpression may reflect a nonspecific shift in cellular state rather than mechanistic relevance to reprogramming. The manuscript implicitly assumes that IRAK4 acts through the same pathways used in innate immunity, but this might represent a "moonlighting" activity rather than a mechanistic necessity. A more cautious interpretation is advised.

Response: We appreciate the reviewer's insightful concern regarding the interpretation of immune-related gene activation upon IRAK4 overexpression. We agree that a cautious distinction between causal drivers and secondary consequences is essential. In this regard, it's reported that chemical reprogramming of human somatic cells into pluripotent stem cells requires suppression of the JNK pathway, which acts by inhibiting pro-inflammatory signaling to enable the induction of cellular plasticity and a regeneration-like program[9]. Notably, this work establishes that the inhibition of inflammatory pathways is a key factor for successful cell fate reprogramming. IRAK4 is a canonical mediator of pro-inflammatory signaling upstream of NF-κB and JNK. In our context, IRAK4 overexpression may thus inadvertently trigger inflammatory responses that oppose the reprogramming process. The observed upregulation of immune-related genes could represent a non-specific stress response or a by-product of pathway activation that the cell must overcome to achieve lineage conversion. This interpretation aligns with the emerging understanding that IRAK family members also possess non-canonical, MyD88-independent functions, including nuclear localization and interaction with chromatin remodeling complexes such as PRC2[2], which can directly influence epigenetic states and stem cell maintenance independent of inflammatory signaling. Therefore, while IRAK4 overexpression does induce immune gene expression, this may reflect either a confounding stress response or a moonlighting activity unrelated to the core reprogramming mechanism. A more parsimonious interpretation is that successful cell fate activation requires fine-tuned suppression of inflammatory signals—as exemplified by the chemical reprogramming paradigm—and that IRAK4 manipulation may impact reprogramming through its non-canonical chromatin-related functions rather than through the immune pathways it canonically governs.

We thank the reviewer for raising this important mechanistic concern. We agree that the observed activation of immune-related genes under IRAK4 overexpression could reflect non-specific cellular stress responses or intermediate-state plasticity rather than direct mechanistic involvement in reprogramming.

10. CaMP-state interpretation may overstate functional plasticity. RNA-seq reveals induction of genes from diverse lineages (neural, renal, pancreatic, etc.) following IRAK4 inhibition. The authors interpret this as entry into a multipotent "CaMP" state. However, this could alternatively reflect:

• transcriptional noise,
• identity destabilization,
• or transient mixed-lineage expression preceding fate acquisition.
Without functional differentiation assays (e.g., directed induction toward neural or renal lineages), the claim of genuine multipotent priming remains speculative.

The conclusions should be balanced accordingly.

Response: We appreciate the reviewer's critical assessment regarding the interpretation of the CaMP state. We have addressed this concern by performing directed differentiation assays. To functionally validate the multipotent potential of IRAK4-inhibited cells, we conducted directed differentiation experiments toward the neural lineage using a standard MEF-to-neuron conversion protocol **(Methods section, page13, lines 481-487)**. Immunofluorescence staining confirmed the generation of $Tuj1^{+}Syn1^{+}$, $Tuj1^{+}vGlut2^{+}$ and $Tuj1^{+}NeuN^{+}$ neuron-like cells (**Response Figure 4 A-B**). Quantitative PCR analysis demonstrated significant upregulation of neural lineage-specific genes including *Tubb3*, *vGlut1*, *Gabbr1*, *Gabbr2* and *Chat* compared to control DMSO and MEFs (**Response Figure 4 C**). These results provide functional evidence that IRAK4 inhibition primes cells toward a multipotent state rather than merely inducing transcriptional noise or identity destabilization. We acknowledge that the CaMP state may represent a transient, metastable condition with mixed-lineage features preceding full fate commitment. We sincerely apologize that we were unable to find pure small-molecule induction protocols for reprogramming toward other lineages (such as renal, pancreatic, etc.), and therefore only expended our functional validation to the induction of neuron-like cells.

In the revised manuscript, we have incorporated Response Figure 4 as main Figure 8. Additionally, we have expanded the relevant context to emphasize the CaMP state during chemical reprogramming (page 7 lines 265-275). We thank the reviewer again for the valuable suggestion.

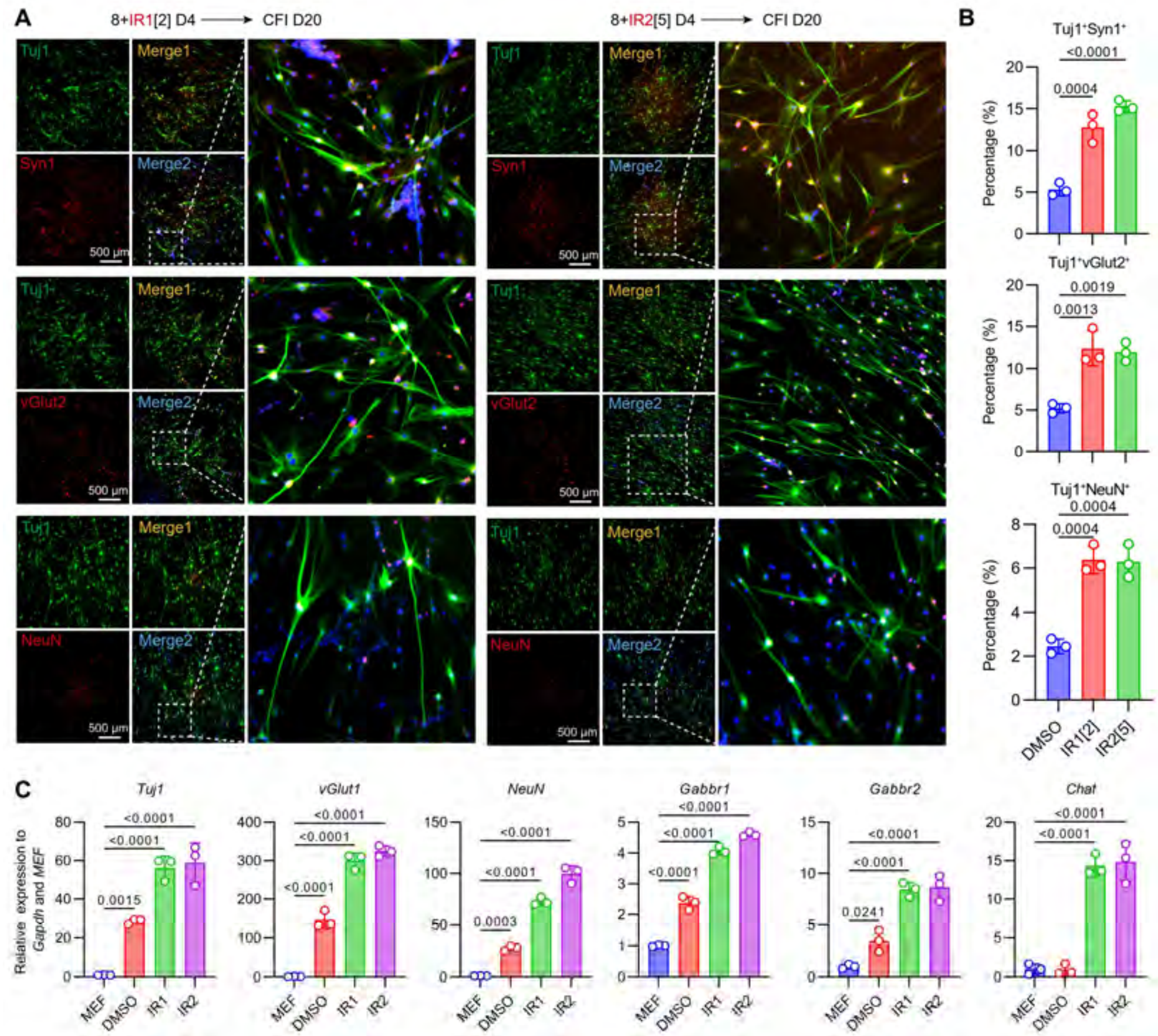

Response Figure 4 IRAK4 inhibitors enhances functional reprogramming to Neuron-like cells

(**A**) Immunostaining of chemically induced neuron-like cells induced by indicated cocktails. Scale bar, 500 μm. CFI: CHIR99021, Forskolin, ISX-9. (**B**) Percentages of Tuj1$^+$Syn1$^+$, Tuj1$^+$vGlut2$^+$ and Tuj1$^+$NeuN$^+$ neuron-like cells. (**C**) Relative mRNA expression of typical neuronal marker genes in neuron-like cells.

Minor Concerns

11. Figure fonts require unification

Figures-particularly Figures 3, 5, 6, and 7-display inconsistent font types and sizes, giving a disorganized appearance. Standardization is needed.

Response: We thank the reviewer for this suggestion regarding figure presentation. We have carefully reviewed and standardized the fonts across all figures, particularly original

Figures 3, 5, 6, and 7 (now renumbered as 3, 6, 7 and 9). All text elements, including axis labels, tick labels, legends, and annotations, now consistently use Arial font to ensure a professional and organized appearance throughout the manuscript. The revised figures have been regenerated using Adobe Illustrator to guarantee vector-quality output with consistent typography that meets publication standards.

12. Volcano plot gene labels unclear. In Figures 5F and 6I, gene annotations in the volcano plots are difficult to read. Other figures should also be carefully reviewed to ensure clarity.

Response: We appreciate the reviewer's comment regarding the readability of gene labels in the volcano plots. We have carefully revised original Figures 5F and 6I (now renumbered as 6G and 7J) by repositioning overlapping labels. Additionally, we have reviewed all other figures (including now renumbered as Figures 3D and 9E) throughout the manuscript to verify that similar label clarity issues do not occur elsewhere and have made corresponding adjustments where needed to maintain consistent visual quality and readability across all figure panels.

13. Comparison with existing reprogramming enhancers. It would be useful to discuss whether IRAK4 inhibition provides higher or comparable reprogramming efficiency relative to previously published small-molecule enhancers. This would help highlight the innovation and practical relevance of the findings.

Response: We appreciate this constructive suggestion. To address the point, we tested several previously reported reprogramming enhancers, including small molecules[10] A-485, WM-8014 and A-485+WM-8014 combination at concentrations of 0.25μM, 0.5 μM and 1 μM, in combination with CFAE+8; however, none of these conditions successfully induced XEN colony formation under our experimental settings (data not shown). We have now incorporated the information into the revised manuscript (page 12 lines 431-434).

14. Nomenclature issue: “shIrak4” used as a substitute noun. The term “shIrak4” is used as a stand-alone noun in several places, which is unconventional and confusing. A more standard phrasing (e.g., “IRAK4 knockdown cells”) should be adopted throughout.

Response: We thank the reviewer for pointing out this important nomenclature issue. We have revised the manuscript throughout to replace the unconventional stand-alone use of “shIrak4” with standard descriptive phrase “IRAK4 knockdown” (page 6 lines 218 and 223, page 10 line 377).

15. English writing issues. Representative examples:

• “have the potential to initiate MEF-XEN reprogramming” is awkward and overly tentative.

• “moderately higher vs slightly elevated” is vague; quantitative fold-change or p-values should be provided instead of subjective descriptors.
A thorough language revision is recommended.

Response: We thank the reviewer for pointing out these important language issues. We have carefully revised the manuscript to improve clarity, precision, and scientific rigor throughout. Specifically, the phrase “have the potential to initiate MEF-XEN reprogramming” has been replaced with more precise wording such as “facilitate the initiation of MEF-to-XEN reprogramming” (page 3 line 82) to reduce ambiguity and better reflect our experimental evidence. We removed “but showing moderately higher expression with E616452” to prevent misinterpretation, as Subcluster 3 shows largely similar gene expression patterns between E616452 and IR1/2. In addition, we conducted a comprehensive language revision of the entire manuscript to ensure professional, clear, and concise English expression, thereby improving readability and overall scientific presentation.

16. The Discussion section feels rather lengthy and could be more concise. I suggest reducing redundancy and improving clarity.

Response: We appreciate the reviewer's suggestion regarding the Discussion section. According to our additional Figures, we have thoroughly revised this section to improve conciseness and clarity by eliminating redundant statements, consolidating overlapping points, and streamlining the overall structure (page 8-12, lines 307-439).

Referee 2:

This study identifies Interleukin-1 Receptor-Associated Kinase 4 (IRAK4), one of innate immune related kinases, as an obstacle to chemical reprogramming in mouse cells. The authors found that pharmacologically inhibiting IRAK4 promotes the generation of extraembryonic endoderm (XEN)-like cells during chemical reprogramming. The role of IRAK4 in regulating XEN cell production was further confirmed through genetic knockdown and overexpression experiments. Mechanistic analyses revealed that IRAK4 inhibition affects the cell cycle, prolonging the G2/M phase and thereby enhancing cellular plasticity. Additionally, IRAK4 suppression facilitates the conversion of mouse fibroblasts into hepatocyte-like cells. While the study uncovers a regulatory target in chemical reprogramming, several major issues need to be addressed to strengthen the reliability and novelty of the conclusions.

1. Although more XEN colonies are generated upon IRAK4 inhibition, the colonies appear smaller compared to those treated with E-616452 and MSC. This seems inconsistent with the proposed mechanism of accelerated cell cycle progression by IRAK4 inhibition.

Please provide a detailed analysis of this observation.

Response: We thank the reviewer for raising this important point regarding the apparent discrepancy between colony size and cell cycle progression. We have carefully analyzed this observation and provide the following detailed explanation:

Our data demonstrate that both IRAK4 inhibitors and E-616452 shorten the G0-G1 phase and prolong the G2-M phase compared to the control 8, indicating accelerated cell cycle progression relative to the control. This conclusion is based on the comparison between the treatment groups and the control (8), not between IRAK4 inhibitors and E-616452 treatment groups.

Regarding colony size, we observed dose-dependent effects of IRAK4 inhibition: At suboptimal concentrations (2 μM in Figure 1A; 0.5, 1, 2 and 10 μM in Figure 2A), IRAK4 inhibitor-induced colonies were indeed smaller than those induced by E-616452 and MSC. However, at the optimal concentration (5 μM), colony sizes were slightly smaller but without statistically significant difference compared to E-616452 (Figure 2A). At the optimal concentrations of IRAK4 inhibitors, E-616452 and MSC, colonies exhibited a wide range of sizes from small to large, with both monolayer and multilayer morphologies observed. These variations are commonly encountered in such experiments.

Therefore, we consider that accelerated cell cycle progression by IRAK4 inhibition and colony size are not mutually exclusive.

2. Previous studies have shown that activation of innate immunity can promote cell fate reprogramming towards iPSCs. In contrast, this study suggests that inhibiting an innate immune kinase facilitates reprogramming. Please explain the potential reasons for this discrepancy and discuss why such differences may arise.

Response: We thank the reviewer for raising this important point. The observation that inhibiting an innate immune kinase (IRAK4) facilitates reprogramming in our study may initially seem to contrast with previous reports demonstrating that activation of innate immunity promotes iPSC generation. However, after carefully considering the relevant literature—including the seminal work by Lee et al. (Cell, 2012) [11], the recent chemical reprogramming studies by Guan et al. (Nature, 2022) [9], and the conceptual framework of "transflammation" articulated by Meng et al. (Antioxid Redox Signal, 2017) [12], we believe these findings are not contradictory. Rather, they reveal the complex and context-dependent roles of innate immune signaling in regulating cellular plasticity.

We consider this apparent discrepancy in light of the distinct experimental systems involved. In the work by Lee et al. (Cell, 2012) [11], innate immune activation occurs through viral or mRNA delivery of reprogramming factors, where the nucleic acid cargo itself serves as a pathogen-associated molecular pattern (PAMP) that engages TLR3. This acute activation triggers rapid epigenetic remodeling and chromatin opening, facilitating transgene expression and subsequent reprogramming. In this context, innate immunity

functions as an exogenous trigger to overcome initial epigenetic barriers. However, as Meng et al. discuss in their review on transflammation, the downstream effectors of these same pathways can function quite differently. As Meng et al. discuss, there appears to be a "Goldilocks zone" for innate immune signaling in reprogramming—an optimal range where signaling is sufficient to promote plasticity but not so excessive as to trigger cell death or senescence[12]. Lee et al. similarly noted that higher levels of TLR3 activation could be counterproductive.

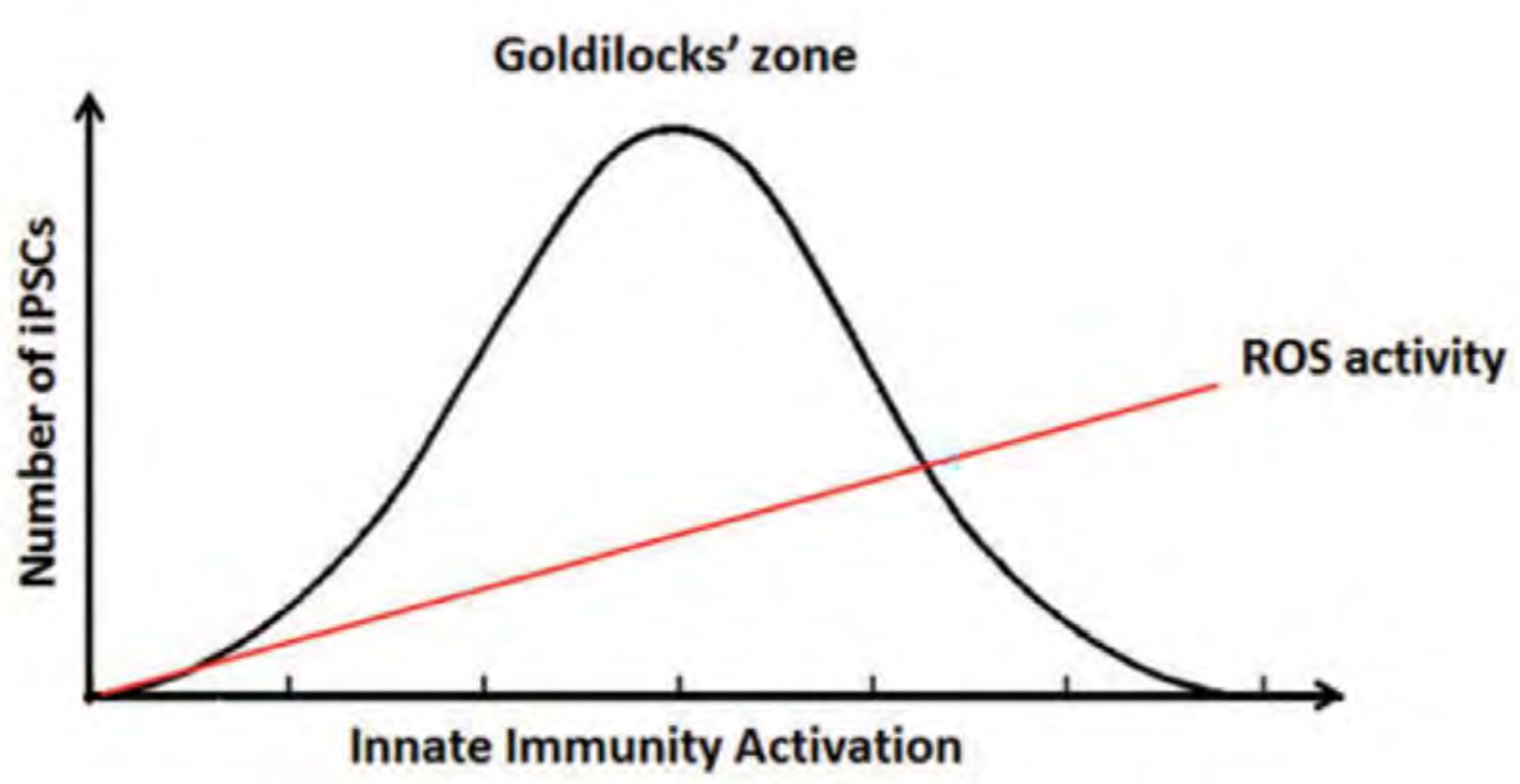

A Goldilocks zone for innate immune signaling and nuclear reprogramming.[12]

In contrast, our study employs chemical reprogramming (lacking viral components or exogenous nucleic acids). Here, we identify IRAK4, a downstream kinase integrating signals from multiple TLR and IL-1 receptor pathways, as an endogenous barrier that stabilizes somatic cell identity. Notably, recent work by Guan et al. (Nature, 2022)[9] demonstrates that chemical reprogramming of human somatic cells requires inhibition of another innate immune kinase (JNK) to suppress pro-inflammatory pathways and induce a regeneration-like plastic state. This aligns well with our findings and suggests that while acute upstream activation (e.g., TLR3 stimulation) may facilitate initial chromatin opening, sustained signaling through downstream effectors (IRAK4, JNK) reinforces somatic identity and must be relieved for successful chemical reprogramming.

Mechanistically, both TLR3 activation (Lee et al.) and IRAK4 inhibition (our study) may ultimately converge on promoting cellular plasticity, albeit through distinct routes. Lee et al. reported TLR3-mediated global changes in epigenetic modifiers. Our data reveal that IRAK4 inhibition enhances chromatin accessibility (Figure 5) and remodels cell cycle dynamics (shortening G0/G1 and lengthening G2/M), creating a permissive temporal window for cell fate transitions. Interestingly, both perturbations activate a similar "regeneration-like" gene program involving LIN28A, SALL4, MSX1, and HOXB9—genes

recently implicated in the intermediate plastic state during human chemical reprogramming (Guan et al., 2022).

In summary, we view our findings not as a contradiction to previous work but as an extension that reveals the multidimensional architecture of innate immune regulation in cell fate decisions. The same network that evolved to defend against pathogens can license the cellular plasticity required for nuclear reprogramming. These observations likely reflect a dual role for innate immunity: transient activation of upstream sensors (TLR3) can initiate plasticity, while constitutive activity of downstream kinases (IRAK4, JNK) maintains somatic identity and must be suppressed. This integrated view addresses the seemingly opposing findings and highlights the importance of context when targeting innate immune pathways for reprogramming applications.

3. The article shows that after treatment with IR1/IR2 or E-616452, cells acquire a CaMP state and express genes associated with different lineages. However, it remains unclear whether these lineage-specific genes are expressed in distinct cell populations or within the same cells. Please investigate and clarify this point.

Response: We thank the reviewer for this important point. To address this question, scRNA-seq technology provides high resolution and is well-suited for this analysis. Due to tha lack of scRNA-seq data from treatment with IR1/IR2, we downloaded and analyzed the single-cell RNA sequencing data (Smart-seq2) for the CFAE+6 condition from the GEO database (accession number: GSE144097). The results demonstrate that genes from all three germ layers can be co-expressed in the same cell, and the proportion of such cells is substantial. For example, cells co-expressing *Gata2*, *Tbx3*, and *Sox11* accounted for 19.785%, and those co-expressing *Sox17*, *Tbx3*, and *Sox11* represented 15.478%. In addition, cells co-expressing two germ layer genes were also observed, such as *Gata2* and *Msx2* double-positive cells at 20.996%. These data indicate that the lineage-specific genes are not restricted to distinct subpopulations but rather co-exist within individual cells. (**Response Figure 5, Response Table 1-2**)

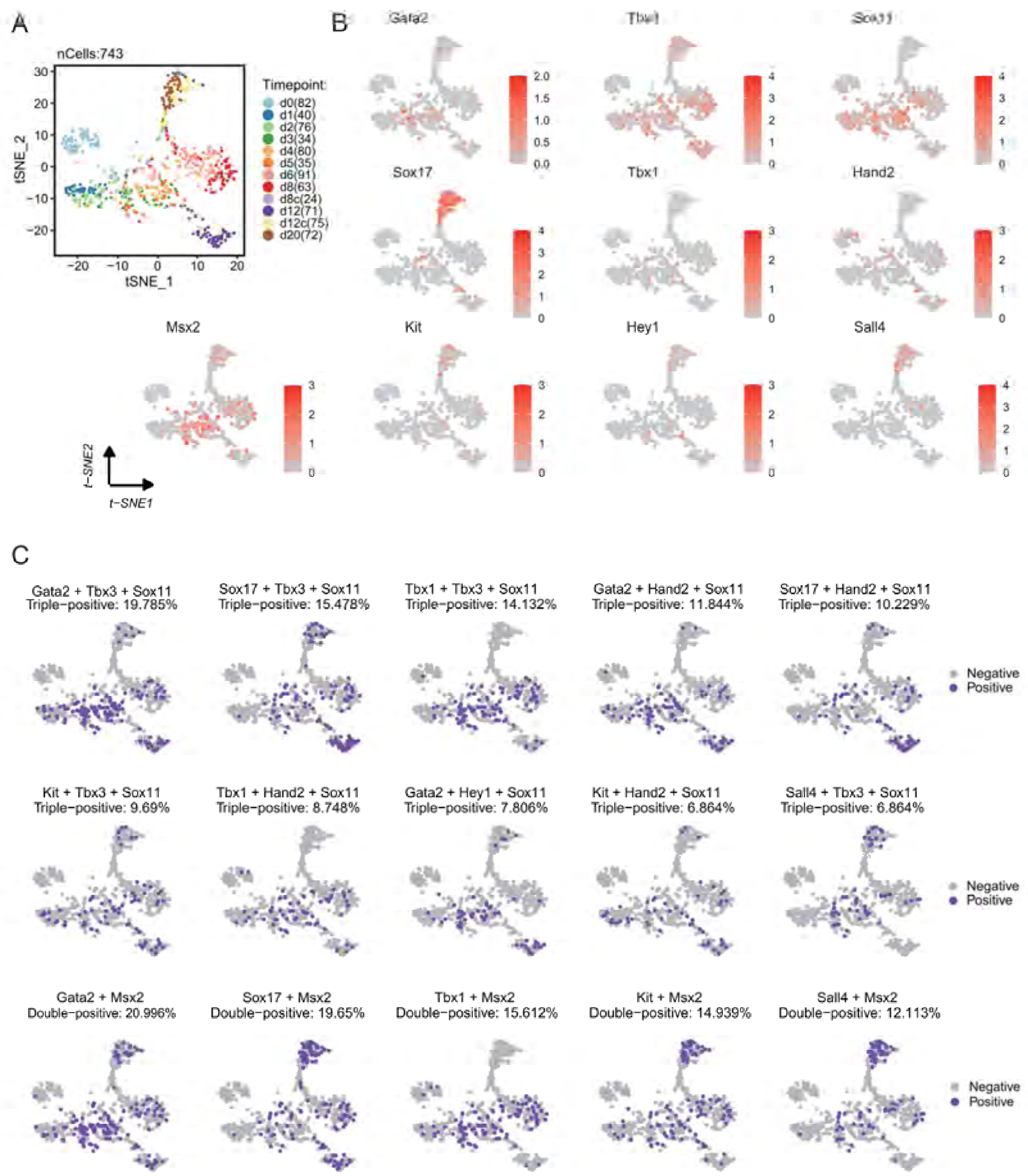

Response Figure 5 (**A**) t-SNE visualization of single cells from the MEF-to-XEN reprogramming time course. Cells were collected at indicated time points (d0 to d20) and analyzed by Smart-seq2. (**B**) Expression patterns of representative marker genes for the three germ layers. (**C**) Co-expression patterns of the top-ranked gene combinations.

Endoderm	Mesoderm & Ectoderm	CoExpRate
Gata2	Msx2	0.2099596
Sox17	Msx2	0.1965007
Tbx1	Msx2	0.1561238
Kit	Msx2	0.1493943
Sall4	Msx2	0.1211306
Nkx6-1	Msx2	0.0632571
Sox17	Zic2	0.0188425
Sall4	Evx1	0.0148048
Gata2	Evx1	0.013459
Kit	Zic2	0.0107672
Gata2	Zic2	0.0107672
Sall4	Zic2	0.0107672
Tbx1	Evx1	0.0107672
Sox17	Evx1	0.0107672
Tbx1	Zic2	0.0094213
Kit	Evx1	0.0094213
Nkx6-1	Evx1	0.0080754
Nkx6-1	Zic2	0.0067295

Response Table 1 The summary of co-expression rates between endoderm and mesoderm & ectoderm genes

Endoderm	Mesoderm	Ectoderm	CoExpRate
Gata2	Tbx3	Sox11	0.1978466
Sox17	Tbx3	Sox11	0.1547779
Tbx1	Tbx3	Sox11	0.141319
Gata2	Hand2	Sox11	0.1184388
Sox17	Hand2	Sox11	0.102288
Kit	Tbx3	Sox11	0.0969044
Tbx1	Hand2	Sox11	0.0874832
Gata2	Hey1	Sox11	0.0780619
Kit	Hand2	Sox11	0.0686406
Sall4	Tbx3	Sox11	0.0686406
Sox17	Hey1	Sox11	0.0632571
Nkx6-1	Tbx3	Sox11	0.0605653
Tbx1	Hey1	Sox11	0.0538358
Gata2	Tbx3	Msx3	0.0457604
Kit	Hey1	Sox11	0.0363392
Nkx6-1	Hey1	Sox11	0.0363392
Nkx6-1	Hand2	Sox11	0.0363392
Gata2	Irx6	Sox11	0.0336474
Tbx1	Tbx3	Msx3	0.0336474
Sall4	Hand2	Sox11	0.0323015
Tbx1	Hey2	Sox11	0.0269179
Gata2	Hey2	Sox11	0.025572
Sox17	Hey2	Sox11	0.025572
Gata2	Hand2	Msx3	0.0242261
Sox17	Tbx3	Pou3f1	0.0228802
Sall4	Hey1	Sox11	0.0228802
Kit	Tbx3	Msx3	0.0228802
Sox17	Tbx3	Ascl1	0.0215343
Sall4	Hey2	Sox11	0.0215343
Tbx1	Irx6	Sox11	0.0201884
Gata2	Hey1	Msx3	0.0201884
Sox17	Tbx3	Msx3	0.0201884
Kit	Tbx3	Ascl1	0.0174966
Gata2	Tbx3	Ascl1	0.0174966
Tbx1	Hey1	Msx3	0.0174966
Sall4	Tbx3	Msx3	0.0174966
Sox17	Hand2	Pou3f1	0.0161507
Gata2	Tbx3	Pou3f1	0.0161507
Tbx1	Tbx3	Ascl1	0.0161507
Tbx1	Hand2	Msx3	0.0161507
Sox17	Hand2	Ascl1	0.0148048
Kit	Hey2	Sox11	0.0148048
Sox17	Irx6	Sox11	0.0148048
Tbx1	Tbx3	Pou3f1	0.013459
Tbx1	Hey1	Ascl1	0.013459

Endoderm	Mesoderm	Ectoderm	CoExpRate
Sall4	Tbx3	Ascl1	0.013459
Kit	Hey1	Msx3	0.013459
Kit	Hand2	Msx3	0.013459
Kit	Hand2	Ascl1	0.0121131
Kit	Irx6	Sox11	0.0121131
Sox17	Hey1	Pou3f1	0.0107672
Tbx1	Hand2	Pou3f1	0.0107672
Tbx1	Hand2	Ascl1	0.0107672
Gata2	Hand2	Pou3f1	0.0094213
Kit	Tbx3	Pou3f1	0.0094213
Gata2	Hey1	Ascl1	0.0094213
Gata2	Hand2	Ascl1	0.0094213
Nkx6-1	Tbx3	Ascl1	0.0094213
Sox17	Hey1	Msx3	0.0094213
Tbx1	Irx6	Msx3	0.0094213
Gata2	Hey1	Pou3f1	0.0080754
Sall4	Tbx3	Pou3f1	0.0080754
Kit	Hey1	Ascl1	0.0080754
Nkx6-1	Hey1	Ascl1	0.0080754
Sox17	Hey1	Ascl1	0.0080754
Tbx1	Hey2	Ascl1	0.0080754
Nkx6-1	Irx6	Sox11	0.0080754
Sall4	Irx6	Sox11	0.0080754
Nkx6-1	Hey1	Msx3	0.0080754
Sall4	Hey1	Msx3	0.0080754
Sall4	Hand2	Msx3	0.0080754
Sox17	Hand2	Msx3	0.0080754
Tbx1	Hey1	Pou3f1	0.0067295
Kit	Hand2	Pou3f1	0.0067295
Nkx6-1	Tbx3	Pou3f1	0.0067295
Sall4	Hand2	Ascl1	0.0067295
Sall4	Hey2	Ascl1	0.0067295
Nkx6-1	Hey2	Sox11	0.0067295
Nkx6-1	Tbx3	Msx3	0.0067295
Gata2	Irx6	Msx3	0.0067295
Kit	Tbx3	Sox3	0.0053836
Sall4	Tbx3	Sox3	0.0053836
Sox17	Hey2	Pou3f1	0.0053836
Sall4	Hey1	Ascl1	0.0053836
Nkx6-1	Hand2	Ascl1	0.0053836
Sox17	Hey2	Ascl1	0.0053836
Sall4	Hand2	Sox3	0.0040377
Tbx1	Tbx3	Sox3	0.0040377
Sox17	Tbx3	Sox3	0.0040377
Sall4	Hand2	Pou3f1	0.0040377

Endoderm	Mesoderm	Ectoderm	CoExpRate
Tbx1	Hey2	Pou3f1	0.0040377
Sall4	Hey2	Pou3f1	0.0040377
Kit	Irx6	Pou3f1	0.0040377
Kit	Hey2	Ascl1	0.0040377
Nkx6-1	Hey2	Msx3	0.0040377
Tbx1	Hey2	Msx3	0.0040377
Gata2	Hey2	Msx3	0.0040377
Sall4	Hey2	Msx3	0.0040377
Sox17	Hey2	Msx3	0.0040377
Kit	Irx6	Msx3	0.0040377
Kit	Hand2	Sox3	0.0026918
Sox17	Hand2	Sox3	0.0026918
Nkx6-1	Tbx3	Sox3	0.0026918
Gata2	Tbx3	Sox3	0.0026918
Sall4	Hey2	Sox3	0.0026918
Kit	Hey1	Pou3f1	0.0026918
Nkx6-1	Hey1	Pou3f1	0.0026918
Nkx6-1	Hand2	Pou3f1	0.0026918
Kit	Hey2	Pou3f1	0.0026918
Gata2	Hey2	Pou3f1	0.0026918
Tbx1	Irx6	Pou3f1	0.0026918
Nkx6-1	Hey2	Ascl1	0.0026918
Kit	Irx6	Ascl1	0.0026918
Nkx6-1	Irx6	Ascl1	0.0026918
Tbx1	Irx6	Ascl1	0.0026918
Gata2	Irx6	Ascl1	0.0026918
Nkx6-1	Hand2	Msx3	0.0026918
Kit	Hey2	Msx3	0.0026918
Tbx1	Hey1	Sox3	0.0013459
Gata2	Hey1	Sox3	0.0013459
Nkx6-1	Hand2	Sox3	0.0013459
Tbx1	Hand2	Sox3	0.0013459
Gata2	Hand2	Sox3	0.0013459
Kit	Hey2	Sox3	0.0013459
Tbx1	Hey2	Sox3	0.0013459
Gata2	Hey2	Sox3	0.0013459
Sox17	Hey2	Sox3	0.0013459
Sall4	Hey1	Pou3f1	0.0013459
Nkx6-1	Irx6	Pou3f1	0.0013459
Tbx1	Irx6	Pou3f1	0.0013459
Gata2	Irx6	Pou3f1	0.0013459
Gata2	Hey2	Ascl1	0.0013459
Sox17	Irx6	Ascl1	0.0013459
Nkx6-1	Irx6	Msx3	0.0013459
Sall4	Irx6	Msx3	0.0013459
Sox17	Irx6	Msx3	0.0013459

Response Table 2 The summary of co-expression rates among three germ layer genes.

4. The manuscript emphasizes IRAK4 as a reprogramming barrier. However, Figure 3i indicates that IR1 and IR2 also inhibit IRAK1. Is IRAK1 similarly a barrier to reprogramming?

Response: We sincerely appreciate the reviewer's insightful question. To address this, we performed additional shRNA-mediated *Irak1* knockdown experiments under CFAE+8 condition. The results showed that *Irak1* knockdown didn't upregulate XEN master genes compared to scramble. Notably, *Gata4* and *Sall4* expressions exhibited down-regulatory trends under Irak1-knockdown conditions. These results suggest that Irak1 may not function as a primary barrier in this context. (**Response Figure 6**)

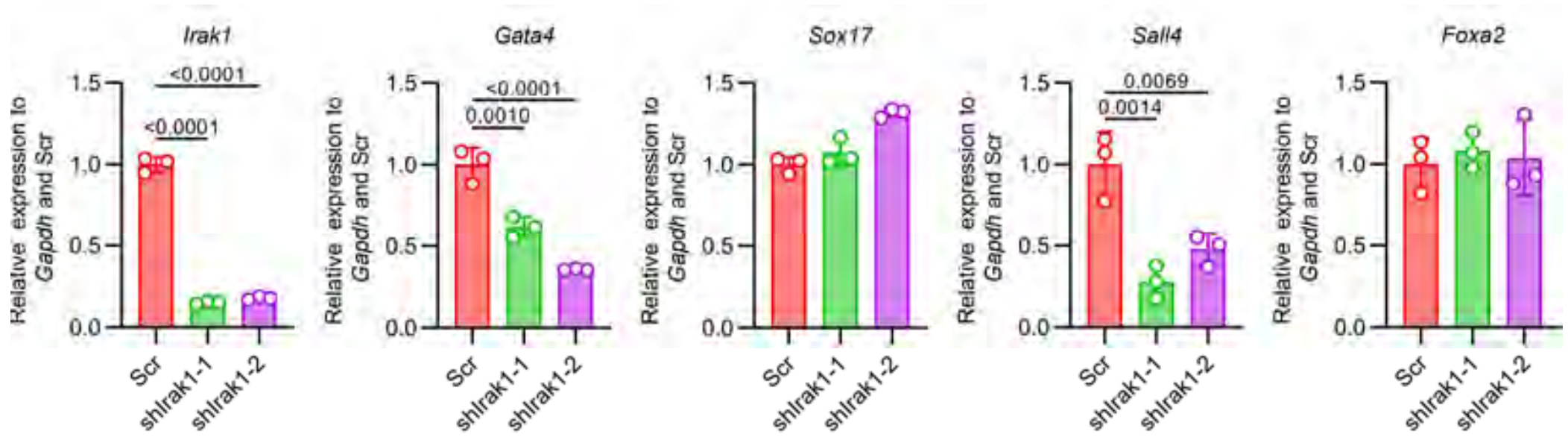

Response Figure 6 Quantitative PCR of *Irak1* and XEN master genes in *Irak1* knockdown conditions.

5. The shRNA-mediated knockdown experiment targeting IRAK4 requires validation of knockdown efficiency to confirm the specificity of the observed effects.

Response: We apologize for omitting this crucial validation in the original manuscript. We have now added comprehensive knockdown efficiency data. (**Response Figure 7**)

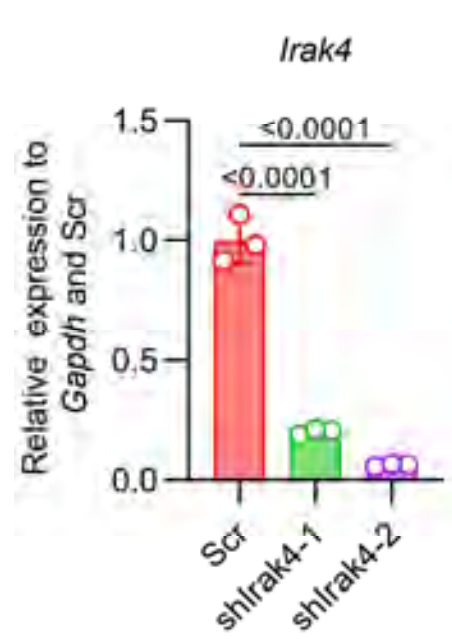

Response Figure 7 Quantitative PCR of *Irak4* in *Irak4* knockdown conditions.

6. In Figure 4f, the differences between condition 8 and conditions 8+IR1/8+IR2 appear minimal. How many independent experiments were performed for these statistical analyses? Please provide the exact n numbers.

Response: We thank the reviewer for requesting clarification on the statistical robustness. Three independent experiments were included in the original manuscript. We have now provided detailed experimental replication data and the corresponding statistical P values

below (**Response Table 3**). We have added (n=3 biological replicates) in Figure 4f legend in revised manuscript.

D8 (%)									
	G0-1			S			G2-M		
MEF	62.94498	63.69337	64.94741	5.75445	5.521845	5.997168	31.42193	30.48139	29.23746
DMSO	28.56803	29.62799	28.96552	2.925089	2.242229	2.079157	69.51928	67.81722	68.25548
8	20.38987	20.94434	21.45697	3.023434	2.793277	2.657274	76.4751	76.60064	75.65909
8+IR1	18.2344	17.36153	16.10545	1.724444	1.607352	1.958628	81.31498	80.93177	80.76145
8+IR2	16.35872	16.49392	16.16113	4.159878	5.959025	2.454328	80.70163	77.42573	80.28564
6	19.78176	19.41563	17.77881	3.704368	3.672063	3.90897	76.79027	76.59643	78.3517

D10(%)									
	G0-1			S			G2-M		
MEF	53.45363	59.23103	57.91568	3.430017	4.431703	4.209106	42.41484	36.75885	38.15514
DMSO	26.49826	24.88655	26.35812	2.492659	2.762947	3.273492	71.03577	72.33716	70.35505
8	25.12764	24.20663	21.08319	4.25468	7.808589	5.105616	70.52758	67.75453	74.13154
8+IR1	16.87218	17.43358	19.05764	4.521303	3.859649	4.531328	78.95739	79.04762	75.7193
8+IR2	20.06622	18.33049	19.66489	4.866058	5.949634	3.110264	75.27842	75.55935	77.17468
6	14.6814	14.77129	14.7613	5.762701	3.974965	4.44437	79.8189	81.11725	80.66782

	P value (T.test)					
	D8			D10		
	G0-1	S	G2-M	G0-1	S	G2-M
8+IR1 vs 8	0.006729	0.001022	0.000312	0.012681	0.157676	0.020171
8+IR2 vs 8	0.001147	0.154469	0.039379	0.030631	0.23596	0.047455
6 vs 8	0.034264	0.001512	0.104208	0.009514	0.233947	0.014939

Response Table 3 The raw data of percentages of cell cycle phases in MEF, CFAE+DMSO, CFAE+8, CFAE+8+IR1/IR2 and CFAE+6 medium cultured cells for 8 or 10 days (n=3 biological replicates), respectively.

Referee #3:

In this study, Li and colleagues attempted to show that the innate immune kinase IRAK4 functions as a previously unappreciated barrier to somatic cell plasticity and chemical reprogramming. Using pharmacological or genetic inhibition of IRAK4, the authors report enhanced multi lineage reprogramming of MEFs through CaMP and XEN like intermediate states, accompanied by increased colony formation and robust activation of core XEN transcriptional regulators. Mechanistically, they propose that IRAK4 suppression reshapes cell cycle dynamics to promote transcriptional plasticity. Importantly, the study further suggests that IRAK4 inhibition broadly facilitates cell fate conversion by significantly improving direct reprogramming of fibroblasts into functional hepatocyte-like cells. Overall, the manuscript is clearly written and logically organized, and most conclusions are supported by the presented data. However, a major limitation of the study is the lack of a clear mechanistic explanation for how IRAK4 inhibition erases somatic identity and facilitates acquisition of XEN or hepatocyte gene signatures.

Major comments:

1. The immunostaining images presented in Figures 1 and S1 are of very low quality. Staining for nuclear transcription factors such as SALL4, SOX17, or GATA4 should exhibit clear nuclear localization, which is not evident in these images. It is unclear whether this reflects image compression during the journal conversion process or suboptimal staining procedures.

Response: We appreciate the reviewer's raising this point. We sincerely apologize for the confusion caused by the image quality in Figures 1 and S1. The primary reason for the suboptimal resolution is that we initially captured these images at low magnification to present the full microscopic field of view, which compromised the clarity of subcellular localizations. Additionally, the image compression in merged pdf file obscured the nuclear localization signals. We confirm that the original uncompressed images do show clear nuclear localization of these transcription factors in many colonies. Regarding the apparent lack of nuclear staining in some clones, we would like to clarify that many of these colonies form multi-layered spherical structures, making it technically challenging to capture nuclear signals from the inner cell layers using normal fluorescence microscopy. However, nuclear localization is clearly observable in the peripheral regions of these colonies. To address this issue, we will provide higher-resolution images and higher-magnification images of Figure 1A in revised manuscript. We apologize for any inconvenience during the review process and thank you again for your valuable suggestion.

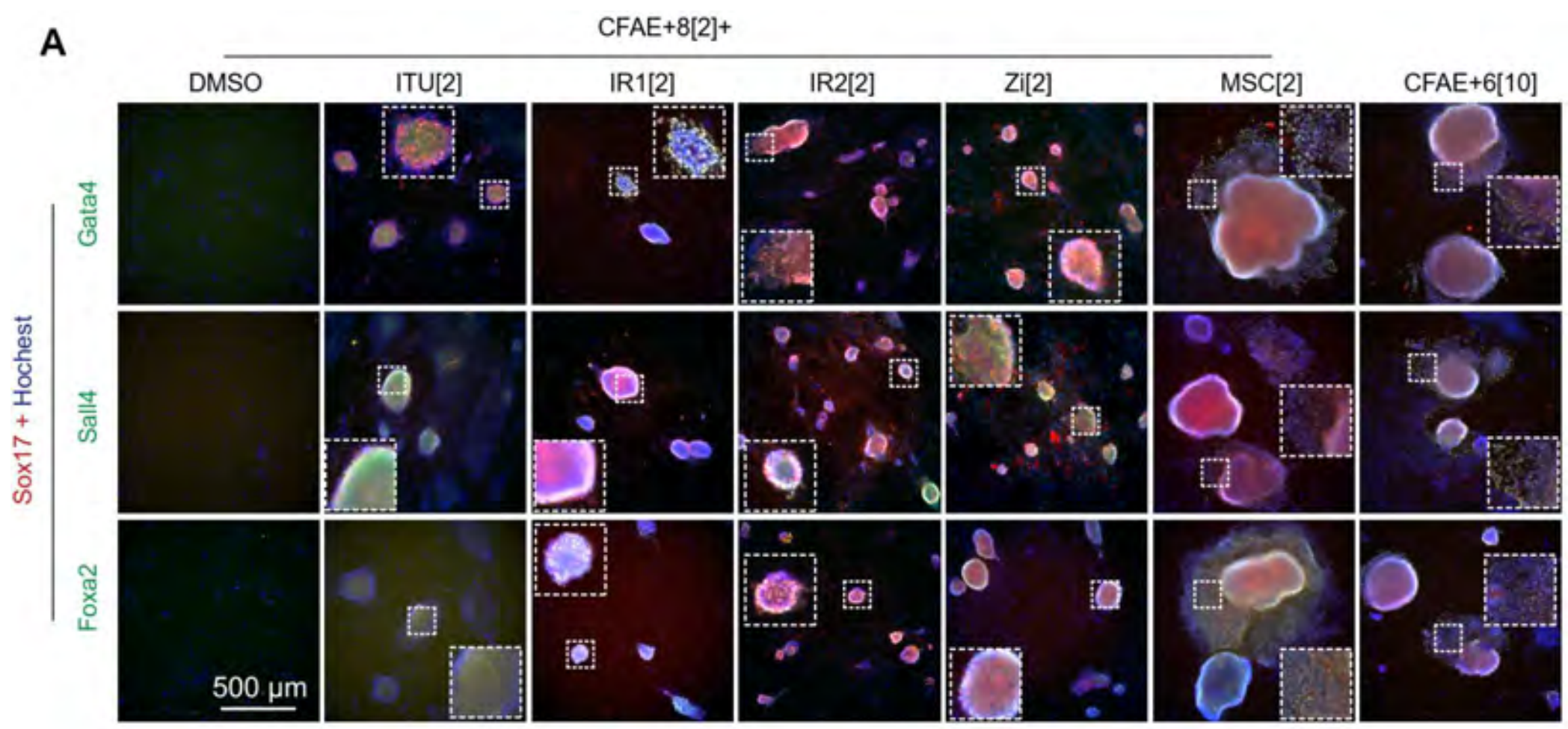

Response Figure 8 Enlarge panels of Figure 1 to show clear nuclear localization.

2. In Figures 1b-d, the authors show that the number of double positive colonies (SOX17 plus an additional XEN marker) is substantially higher in the CFAE+8+IR2 condition compared to CFAE+6. However, in Figures 1e-h, expression levels of all tested XEN

genes are markedly higher in CFAE+6. How can these observations be reconciled? Does the CFAE+6 condition induce stronger XEN gene expression per colony, whereas CFAE+8+IR2 increases colony number with low XEN gene expression? What biological conclusions can be drawn from this discrepancy?

Response: We thank the reviewer for raising this insightful point regarding the apparent discrepancy between colony number and gene expression data. We would like to clarify that this observation can be reconciled by considering differences in colony size. In Figure 1, the CFAE+8+IR2 condition (with 2 μM IR2) indeed generated a higher number of double-positive colonies; however, these colonies were notably smaller than those in the CFAE+6 condition, likely contributing to the lower XEN gene expression levels observed. To address this issue, we subsequently optimized the IR2 concentration to 5 μM (Figure 2A), which yielded comparable colony sizes and similar even higher mRNA expression levels of XEN genes (**Response Table 4**). We apologize for not having adequately explained this point in the original manuscript and have now added this clarification to the revised text. We thank the reviewer again for this valuable reminder.

gene_id	gene_name	DMSO_	DMSO_	8_1	8_2	8_IR1_1	8_IR1_2	8_IR2_1	8_IR2_2	6_1	6_2
ENSMUSG00000025902	Sox17	0.0325	0.0196	1.6491	1.5245	15.881	14.896	25.274	22.662	18.158	16.488
ENSMUSG00000021944	Gata4	0.0795	0.0957	0.2618	0.4545	3.9047	4.1837	3.408	4.6085	2.5501	2.5022
ENSMUSG00000027547	Sall4	0.3504	0.2699	0.3877	0.6891	1.4634	1.5805	2.2185	3.1798	2.2313	2.3016
ENSMUSG00000037025	Foxa2	0	0.0394	0.5606	0.8236	5.1089	5.4148	2.591	2.3211	0.9335	1.4785

Response Table 4 The FPKM values of XEN-regulator genes in indicated samples.

3. The authors state that "both treatments activated genes associated with cell fate specification and markers representative of all three germ layers" (Figure 3F). However, little to no expression of endoderm markers is detectable in CFAE+8+IR1 or CFAE+6 conditions in the corresponding figure. This statement should be revised or better supported.

Response: We thank the reviewer for this careful observation. We have re-examined the expression data presented in Figure 3F and agree that the original statement was overly generalized and did not accurately reflect the differences among the three conditions. Specifically, in the CFAE+8+IR1 and CFAE+6 conditions, endodermal markers showed little to no detectable expression, whereas CFAE+8+IR2 exhibited broader activation across all three germ layers.

To address this concern, we have revised the corresponding sentence in the Results section to accurately describe the expression patterns observed for each condition. The revised text (page 4, line 139-146) now provides a condition-specific description without altering any data.

4. Clear transcriptional differences exist between CFAE+8+IR1, CFAE+8+IR2, and CFAE+6, including in core XEN markers. For example, Foxa2 is not upregulated in CFAE+6 (Figure 3I). The authors are encouraged to perform a deeper comparative

analysis highlighting all major transcriptional differences between these three conditions, beyond GO term enrichment (Figures 4c-d), and to propose potential explanations for these differences.

Response: We thank the reviewer for this insightful comment. About Foxa2 expression, we downloaded the single-cell RNA sequencing data (Smart-seq2) for the CFAE+6 condition from the GEO database (accession number: GSE144097) and performed a re-analysis of the Foxa2 expression dynamics during the reprogramming process. Our analysis revealed that Foxa2 expression was indeed very low during the early stages of induction in the CFAE+6 condition, with significant upregulation only becoming apparent after day 8 of induction (**Response Figure 9**). In this study, RNA-seq data were obtained from cells on day 8, which could indeed show that Foxa2 is not upregulated in CFAE+6 (Figure 3I).

Additionally, to address the transcriptional differences among the CFAE+6, CFAE+8+IR1, and CFAE+8+IR2 conditions, we performed deeper comparative analyses based on the RNA-seq data. As shown in **Response Figure 10-11**, although statistically significant differentially expressed genes were identified between each pair, these genes were primarily enriched in metabolic processes, signal transduction, cell locomotion, and cell-cell interaction pathways, with no significant enrichment in other lineage developmental trajectories except for an enrichment in kidney or renal system development both in CFAE+6 vs CFAE+8+IR1 and CFAE+8+IR2 vs CFAE+8+IR1. These findings suggest that their transcriptomic distinctions primarily reflect variations in cellular metabolic states and intercellular communication rather than fundamental divergence in cell identity determination. These results have been summarized in a new supplementary figure (Figure EV4-5).

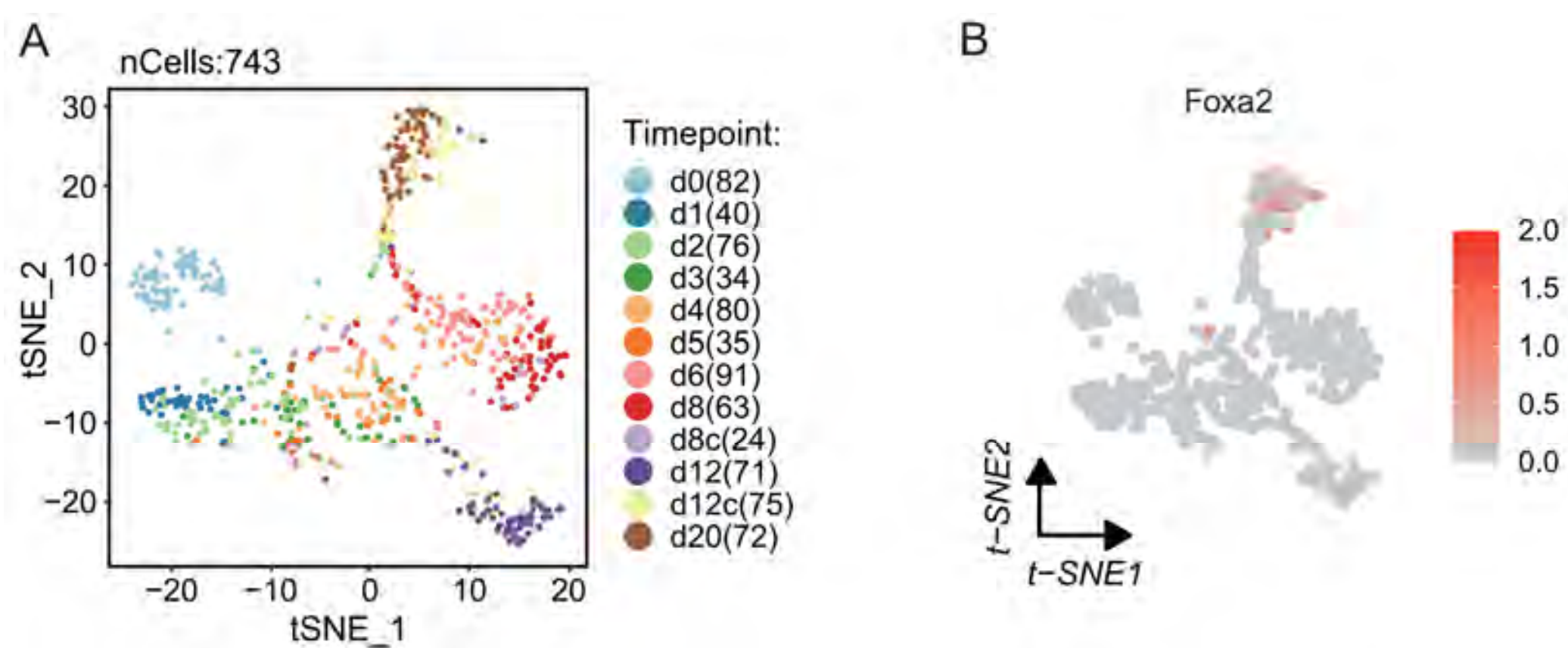

Response Figure 9 (A) t-SNE visualization of single cells from the MEF-to-XEN reprogramming time course. Cells were collected at indicated time points (d0 to d20) and analyzed by Smart-seq2. **(B)** Expression pattern of *Foxa2* in t-SNE visualization of scRNA-seq data.

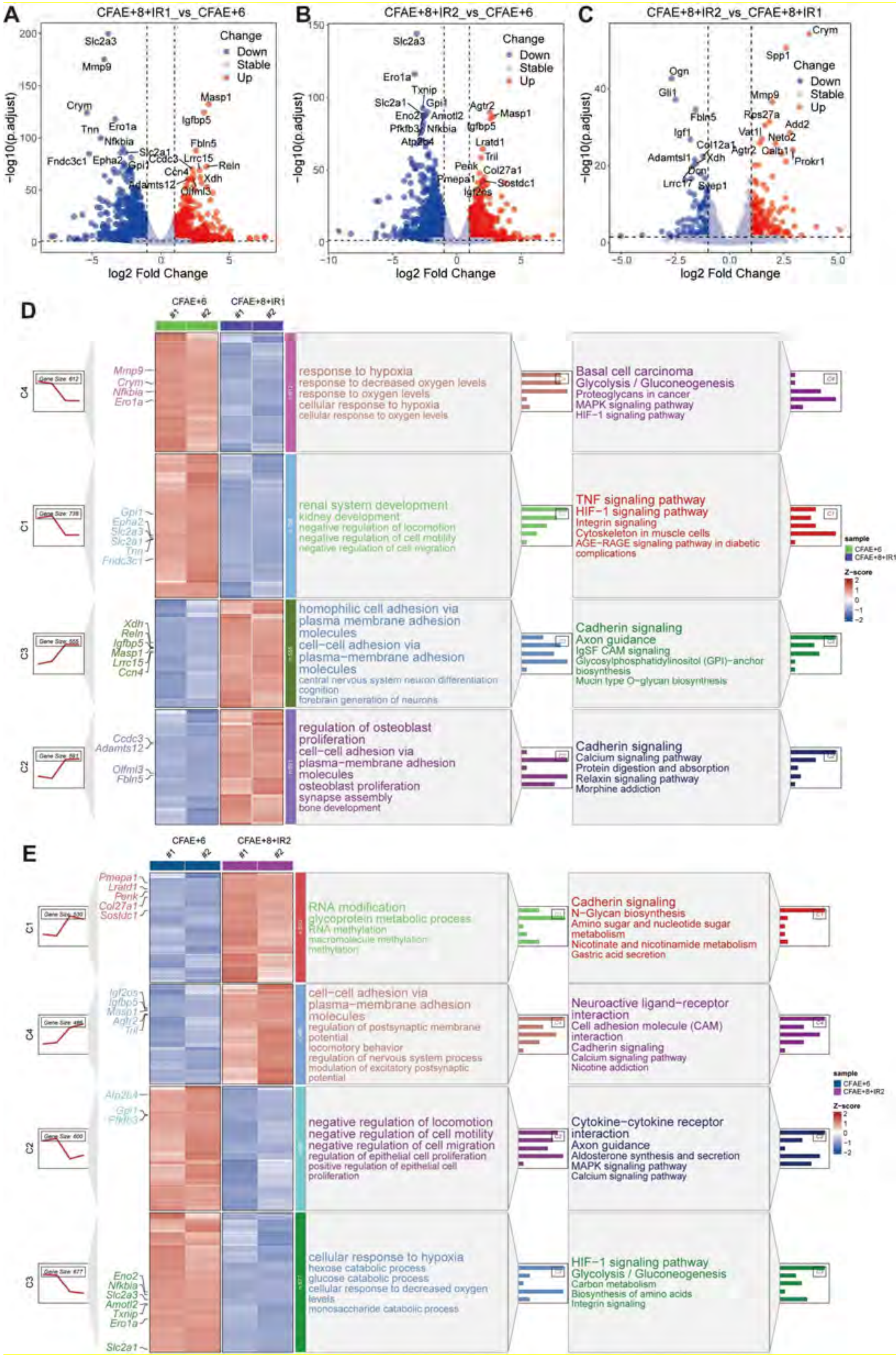

Response Figure 10 (A)-(C) Volcano plot of CFAE+8+IR1, CFAE+8+IR2 versus CFAE+6, or CFAE+8+IR2 versus CFAE+8+IR1. Significantly up- and down-regulated genes are defined by |log2FC| > 1 and *p.adjust* < 0.05. **(D) and (E)** Heatmap, GO and KEGG

enrichments of differentially expressed transcripts in CFAE+8+IR1 or CFAE+8+IR2 versus CFAE+6. Enriched GO-BP and KEGG pathways are displayed with font size scaled to significance.

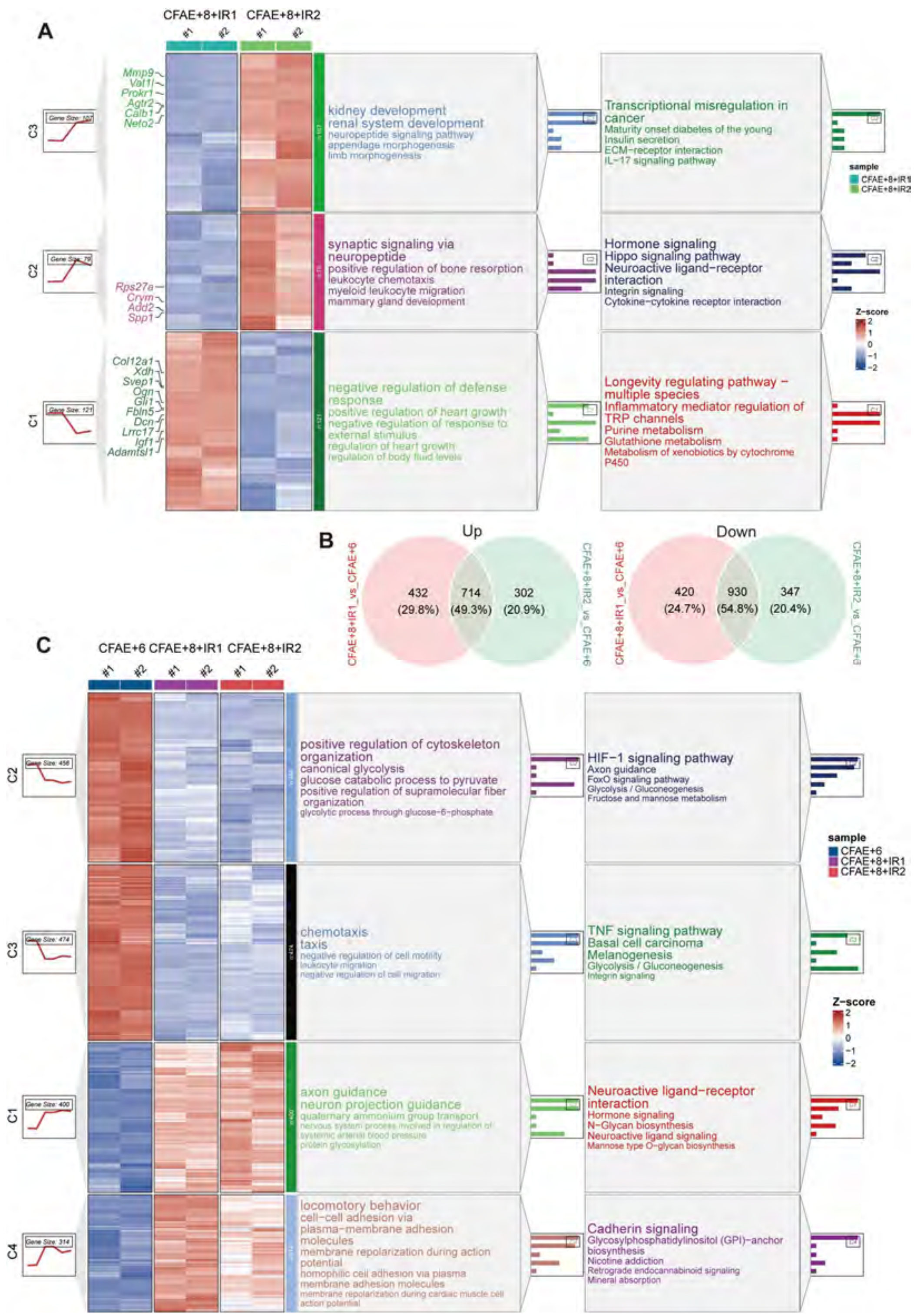

Response Figure 11 (A) Heatmap, GO and KEGG enrichments of differentially expressed transcripts in CFAE+8+IR2 versus CFAE+8+IR1. Enriched GO-BP and KEGG pathways are displayed with font size scaled to significance. **(B)** Venn diagrams show the common down- or up-regulated genes between CFAE+8+IR1 versus C6FAE and CFAE+8+IR2 versus C6FAE. **(C)** Heatmap, GO and KEGG enrichments of transcripts in the intersection of comparisons. Enriched GO-BP terms and KEGG pathways for each cluster are displayed, with font size scaled to enrichment significance.

5. The authors show that IRAK4 and IRAK1 expression is not detected in CFAE+6. To determine whether the effects of IR1 and IR2 are indeed mediated specifically through IRAK4/1 inhibition, the authors should test CFAE+6 conditions with and without IR1 or IR2 and quantify colony numbers. An increase in colonies upon IR1 or IR2 addition would suggest IRAK independent effects of these inhibitors.

Response: We thank the reviewer for this insightful suggestion. As recommended, we performed additional experiments to determine whether the effects of IR1 and IR2 are specifically mediated through IRAK4/1 inhibition.

In the CFAE+6 condition, we treated cells with IR1 or IR2 and quantified colony formation. We found that the addition of IR1 or IR2 did not result in any significant change in colony numbers compared to the untreated CFAE+6 control. In addition, the expression levels of XEN marker genes remained largely unchanged upon IR1 or IR2 treatment under these conditions. These results indicate that IR1 and IR2 do not exert detectable effects on colony formation or XEN marker expression (**Response Figure 12**). This supports the conclusion that the effects of IR1 and IR2 observed in other conditions are specifically mediated through IRAK4/1 inhibition, rather than through IRAK-independent effects.

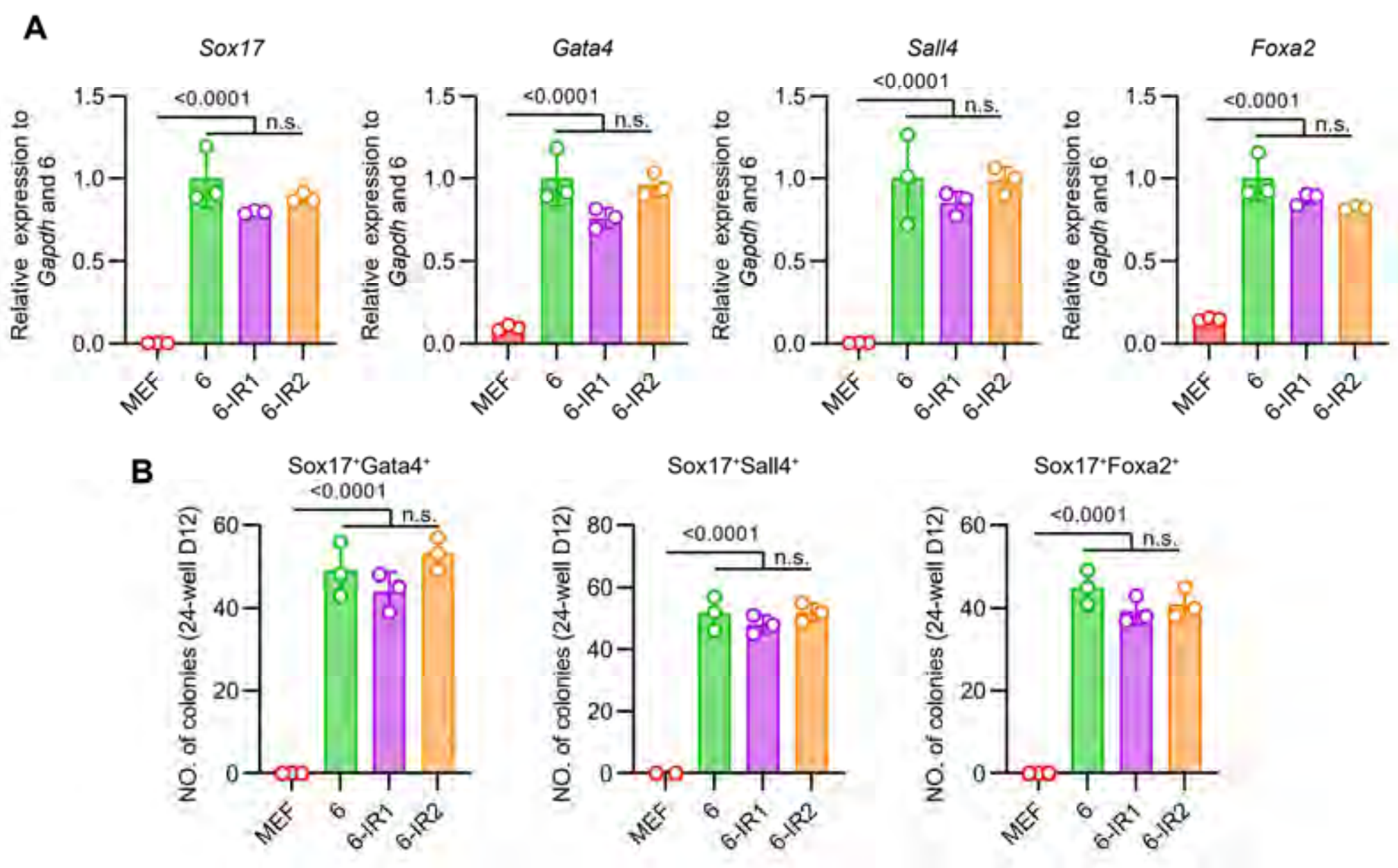

Response Figure 12 (**A**) Quantitative PCR for *Sox17*, *Gata4*, *Sall4*, and *Foxa2* in CFAE+6 plus IR1 or IR2 for 12 days, respectively. (**B**) The numbers of Sox17^{+}Gata4^{+}, Sox17^{+}Sall4^{+} and Sox17^{+}Foxa2^{+} colonies in (**A**). Data are represented as mean ± SD. Numbers in each panel are P-values between 6 and indicated samples (n=3 biological replicates, respectively). *P* values were calculated using one-way ANOVA followed by Dunnett multiple comparisons test.

6. Although CFAE+8+IR1, CFAE+8+IR2, and CFAE+6 slightly reduce the G0/G1 phase relative to CFAE+8, the reviewer is not convinced that this modest change in cell cycle dynamics is the key determinant enabling XEN induction. The major shift in cell cycle distribution already occurs with CFAE+8 alone. Therefore, the abstract statement that "IRAK4 inhibition remodels cell cycle dynamics, shortening G0/G1 and lengthening G2/M phases to license transcriptional plasticity" is not sufficiently supported. To strengthen this claim, the authors should test whether accelerating the cell cycle under CFAE+8 conditions alone (for example using p53 knockout MEFs) is sufficient to induce a XEN like transcriptional program. Such experiments would directly assess whether cell cycle acceleration per se accounts for the IR1/IR2-mediated induction of XEN gene expression.

Response: We thank the reviewer for this insightful and constructive suggestion (similar to point 2 of Referee #1). Indeed, multiple studies have demonstrated that suppression or loss of TP53 facilitates somatic cell reprogramming toward induced pluripotent stem cells (iPSCs)[3-6] under OSKM inducible system. Based on this rationale, we initially planned to test whether accelerating the cell cycle under CFAE+8 conditions alone, for example using TP53 knockout MEFs (suggested by Referee #3), would be sufficient to induce a XEN-like transcriptional program. However, after consulting multiple mouse vendors and other alternative strategies, we found that homozygous TP53 knockout mice frequently

exhibit severe developmental defects, compromised viability, or embryonic lethality, making it extremely difficult to reliably obtain suitable embryos for MEF derivation. Consequently, we were unable to carry out this experiment as originally proposed. Alternatively, we employed shRNA-mediated knockdown of Trp53 (shTrp53) to establish Trp53-deficient conditions. However, Trp53 depletion alone failed to induce XEN-like colony formation. Unlike the OSKM system, which alone can induce pluripotent conversion, the CFAE+8 system alone cannot. Therefore, these two systems are distinct, and it seems reasonable that Trp53 knockdown in these systems does not yield similar results, which was inconsistent with the indispensable roles of Trp53 in human CiPSC induction[7].

As an alternative approach, we investigated cell cycle modulation using small-molecule inhibitors AT9283 (an Aurora kinase A/B inhibitor) and Barasertib-HQPA (a selective Aurora kinase B inhibitor) under CFAE+8+IR2 conditions. Notably, treatment with either inhibitor completely abolished the formation of XEN-like colonies, indicating that proper cell cycle progression is indeed an important factor supporting reprogramming (**Response Figure 1**). However, since this approach only demonstrates that disruption of the cell cycle impairs reprogramming, rather than proving that cell cycle acceleration is sufficient to induce XEN-like fate under CFAE+8 conditions, we agree with the reviewer that cell cycle remodeling alone cannot fully account for IR1/IR2-mediated effects. According to the suggestion of Referee #1, we investigated the chromatin landscape and found that enhanced chromatin accessibility underlies the mechanism by which IR2 promotes cell reprogramming (**Response Figure 2**).

Therefore, we have revised the manuscript to moderate our conclusion and clarify that cell cycle regulation represents an important, but not exclusive, contributor to the acquisition of XEN-like identity (page 9 lines 343-349).

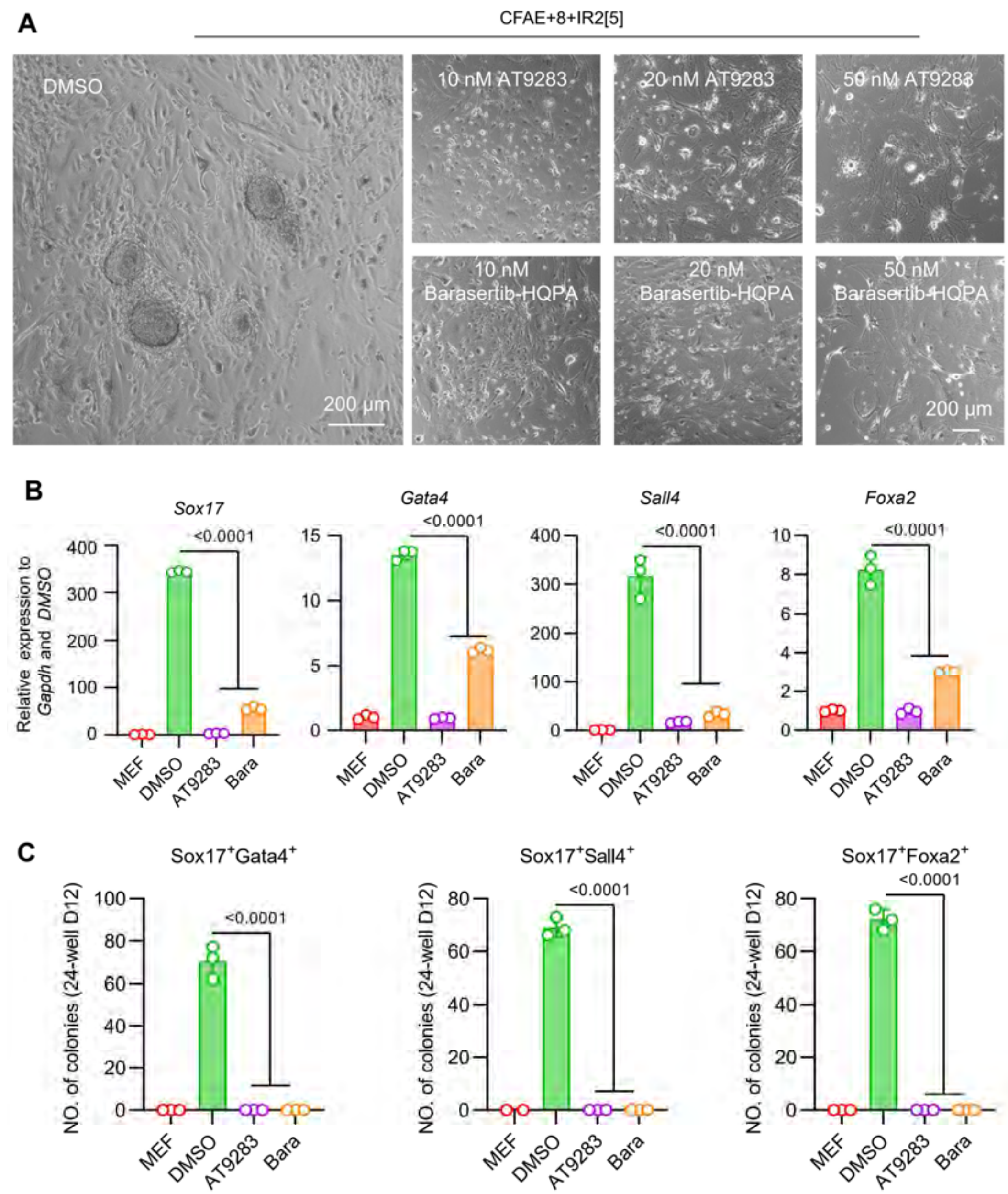

Response Figure 13 Disruption of the cell cycle impairs cell reprogramming

(**A**) Morphology of colonies or cells induced by indicated cocktails in bright field. Scale bar, 200 μm. (**B**) Relative mRNA expression of *Sox17, Gata4, Sall4,* and *Foxa2* in indicated samples in MEF or CFAE+8+IR2 basal medium for 12 days, respectively. Bara: Barasertib-HQPA. (**C**) Percentages of Sox17$^+$Gata4$^+$, Sox17$^+$Sall4$^+$, and Sox17$^+$Foxa2$^+$ colonies. **(Same to Response Figure 1)**

7. As noted above, immunostaining in Figures 5a-c is not convincing due to the lack of clear nuclear localization of the indicated transcription factors.

Response: We thank the reviewer for raising this concern regarding the immunostaining in original Figures 5a–c. The absence of clear nuclear localization was primarily attributable to image compression in merged pdf. To address this, we have now replaced the images with representative images from a single clone, which clearly demonstrates the nuclear localization. (**Response Figure 14**)

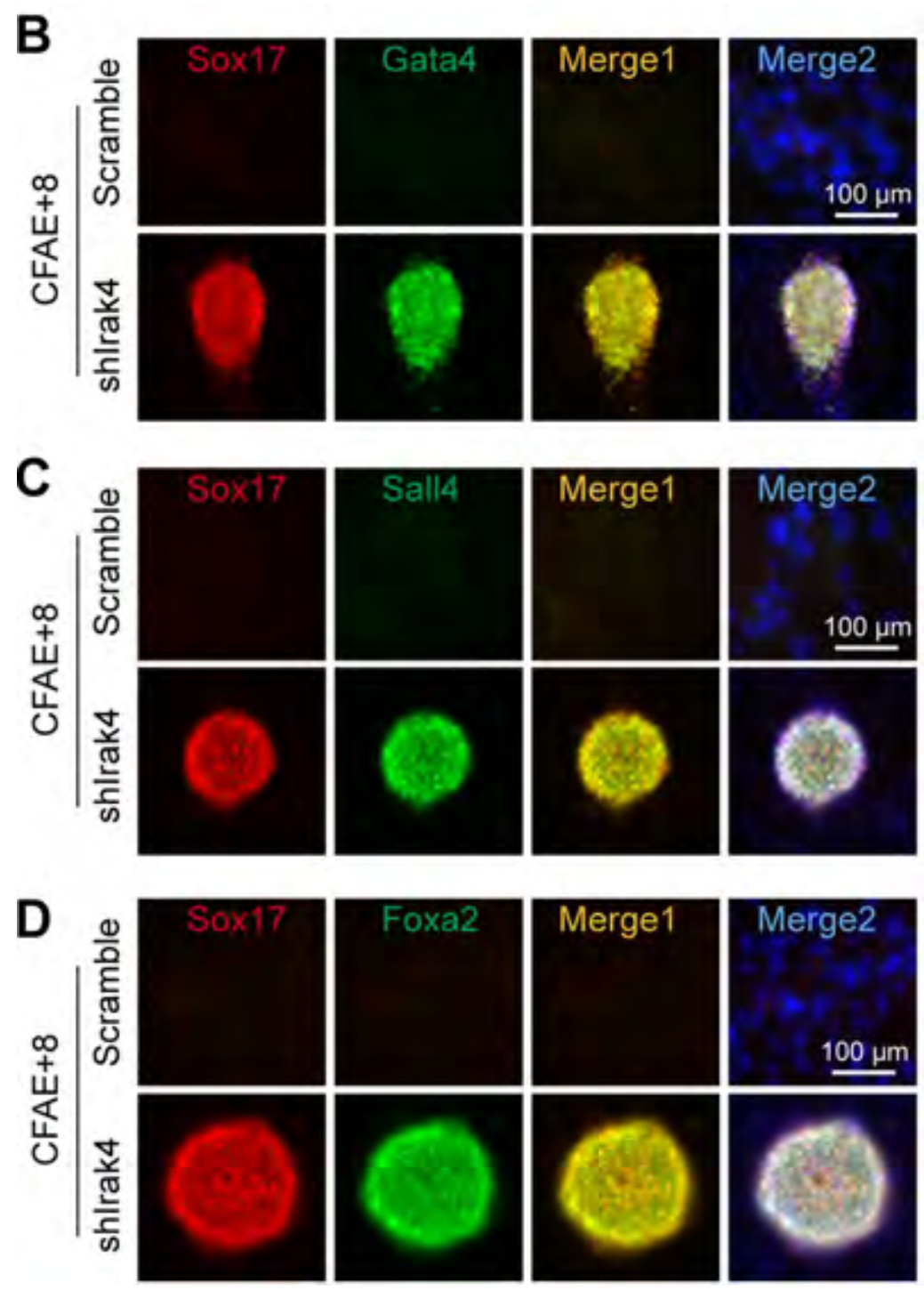

Response Figure 14 (**A**)-(**C**) Immunofluorescence co-staining of Sox17 and Gata4 (**A**), Sall4 (**B**) or Foxa2 (**C**) in *scramble* and *shIrak4* under CFAE+8 induction for 16 days, respectively.

8. In Figure 5E, the two independent shRNAs targeting IRAK4 induce non-consistent activation of XEN markers. While both shRNAs downregulate IRAK4 to a similar extent, SOX17 and SALL4 are much more strongly induced by shRNA-1, whereas GATA4 is preferentially induced by shRNA-2. The authors should address and explain this inconsistency.

Response: We thank the reviewer for this careful observation. We apologize for the confusion caused by original Figure 5E. To address this inconsistency, we have repeated the knockdown experiments and performed qPCR analysis. The new qPCR results consistently show that both independent shRNAs targeting IRAK4 similarly upregulate the expression of XEN regulator genes (**Response Figure 15**). The revised figure (new additional Figure 6A) with qPCR validation has been included in the updated manuscript.

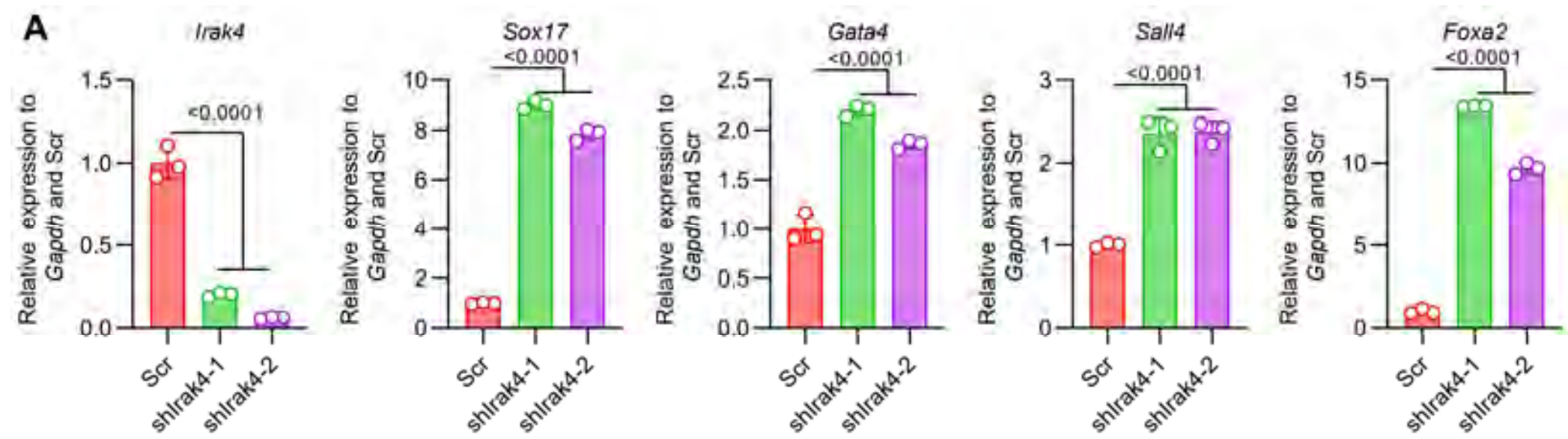

Response Figure 15 Quantitative PCR for *Sox17*, *Gata4*, *Sall4*, and *Foxa2* of scramble, shIrak4-1 and shIrak4-2 in CFAE+8 for 12 days, respectively

9. In Figure 6, IRAK4 overexpression blocks MEF to XEN conversion, but the authors do not describe the effects of IRAK4 overexpression on cell cycle progression. Do these cells stop proliferating? If so, impaired proliferation could readily explain the failure of reprogramming. The reviewer suggests overexpressing IRAK4 in p53-deficient fibroblasts and re-testing reprogramming efficiency.

Response: We thank the reviewer for this important and insightful suggestion. We agree that impaired proliferation could be a potential explanation for the reduced reprogramming efficiency upon IRAK4 overexpression. In our current study, although we did not observe a complete cessation of cell growth in IRAK4-overexpressing cells, we cannot exclude the possibility that IRAK4 overexpression may partially affect cell cycle progression or proliferative capacity, which in turn could influence reprogramming outcomes. To directly address whether enhanced proliferation could rescue the inhibitory effect of IRAK4 overexpression, the reviewer's suggestion to perform experiments in p53-deficient fibroblasts is well taken. Unfortunately, as noted above, we encountered significant technical challenges in obtaining TP53-deficient MEFs, which limited our ability to perform this experiment. Like our response to question 6 that Trp53 was indispensable to chemical reprogramming[7], we speculate that overexpressing IRAK4 in p53-deficient fibroblasts will fail to induce cell reprogramming. Although we currently cannot obtain p53-KO MEF, once we have the opportunity to acquire it in the future, we will prioritize testing this result.

10. For hepatocyte reprogramming, it is unclear whether the PCR assays detecting Hnf4a and Foxa3 measure endogenous or exogenous transcripts. Demonstration of endogenous activation is essential to support successful reprogramming.

Response: We thank the reviewer for raising this important point. To address this concern, we have re-examined the hepatocyte-like cells induced by both the *Hnf4a+Foxa3* overexpression system and the chemical induction approach. Our data demonstrate that in the *Hnf4a+Foxa3* system, both IR1 and IR2 significantly upregulated endogenous

Hnf4a expression, with IR2 showing higher efficacy, whereas neither condition could upregulate endogenous *Foxa3*. Given that *Hnf4a,* rather than *Foxa3* serves as the principal marker gene of hepatocyte identity and considering the suboptimal performance of IR1 relative to IR2, we subsequently focused our validation on IR2 in the chemically induced hepatocyte-like cells (CiHeps). The results confirmed that IR2 successfully upregulated both endogenous *Hnf4a* and *Foxa3*, thereby satisfying the essential requirement for demonstrating endogenous activation of reprogramming factors and supporting the successful induction of hepatocyte-like cells.

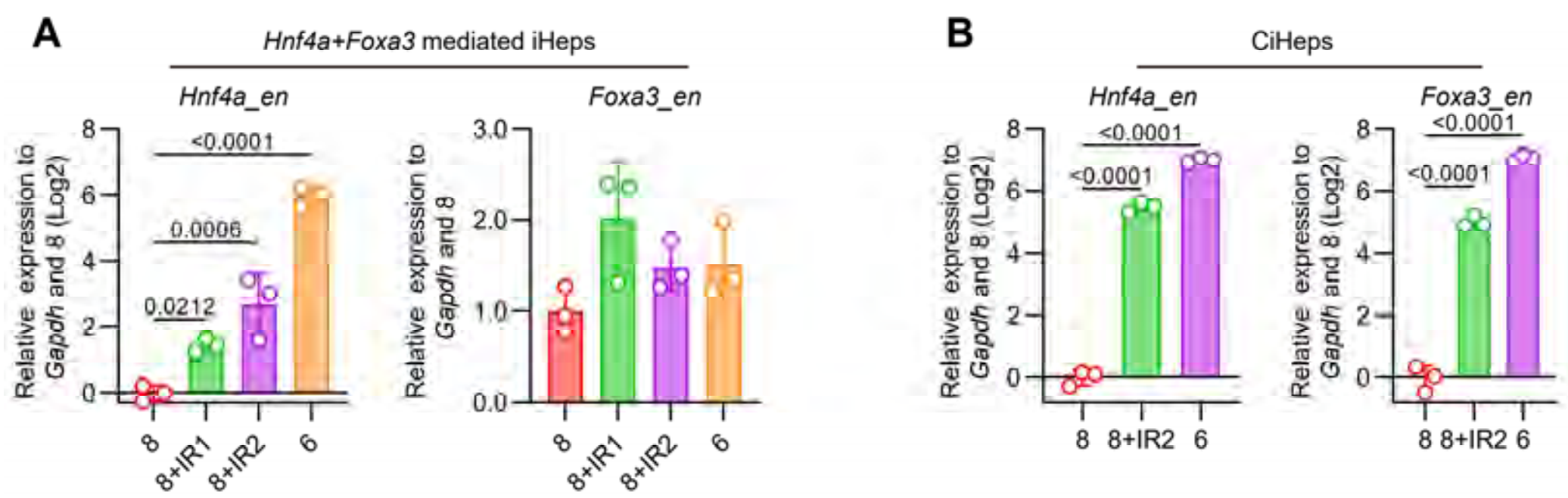

Response Figure 16 (**A**) and (**B**) Quantitative PCR of endogenous *Hnf4a* and *Foxa3* mRNA in Hnf4a+Foxa3 mediated iHeps (**A**) and CiHeps (**B**).

11. The immunostaining images in Figure 7 are of low quality and difficult to interpret. The reviewer is not convinced by the reported hepatocyte like morphology.

Response: We appreciate the reviewer's feedback regarding the image quality in original Figure 7. To facilitate clearer visualization and interpretation of the hepatocyte-like cellular morphology, we have now included higher-magnification views of representative regions, which allow for more detailed examination of the characteristic cell shape, prominent nuclei, and cytoplasmic granularity typical of hepatocyte-like cells, and we hope these enlarged images provide sufficient detail to convincingly demonstrate the reported morphological features.

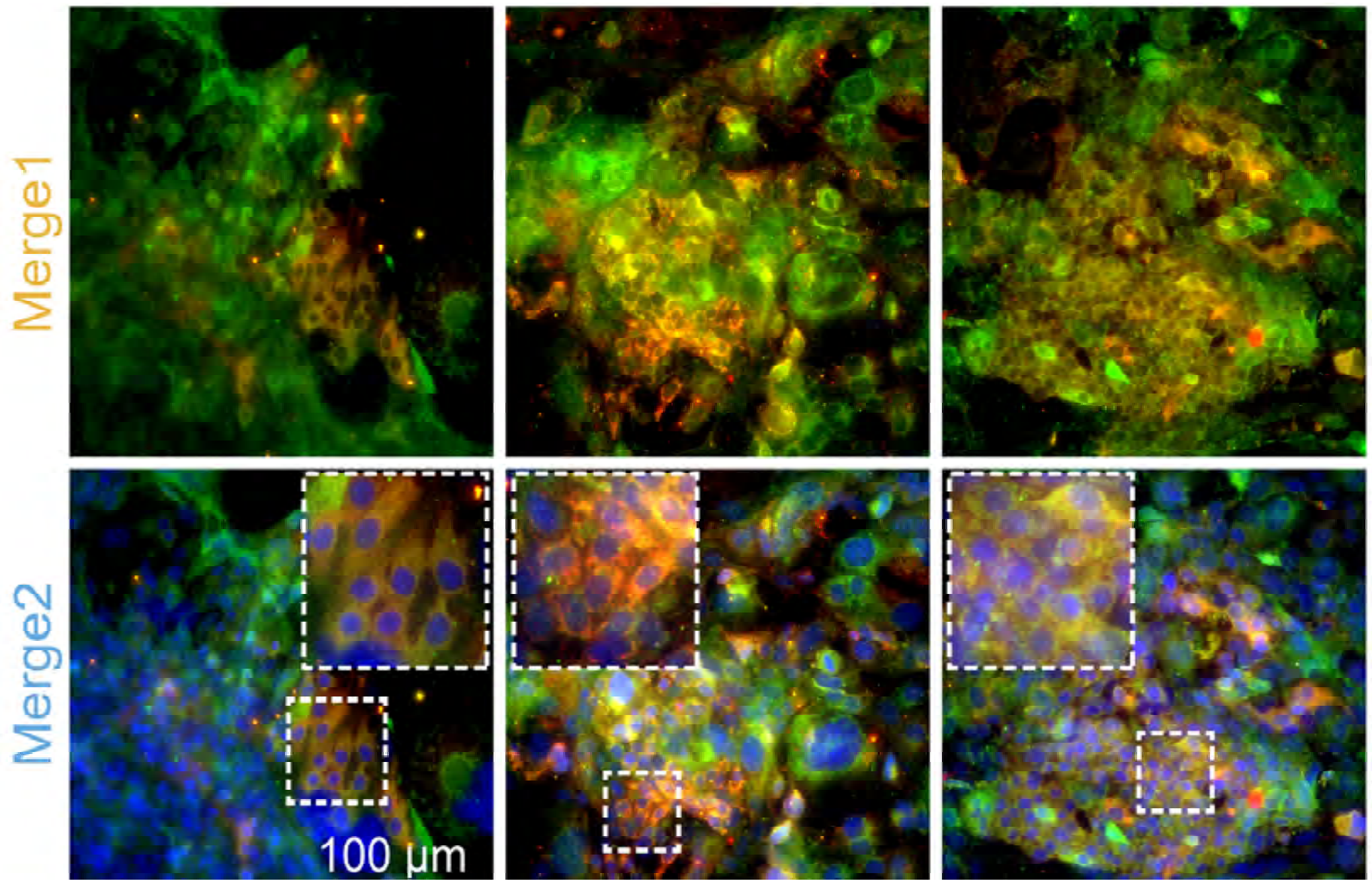

Response Figure 17 Enlarged images to show the hepatocyte like morphology

12. Do IR1 and IR2 similarly accelerate the cell cycle during hepatocyte conversion? Additionally, what explains the differences in hepatocyte reprogramming efficiency between IR1 and IR2?

Response: We thank the reviewer for this important question. Our RNA-seq data did not enrich cell cycle related GO terms during hepatocyte conversion upon treatment with IR1 or IR2, suggesting that these inhibitors do not markedly accelerate the cell cycle in this context. However, when we quantified the number of $ALB^{+}CYP3A11^{+}$ cells within individual colonies, we found that IR1- and IR2-treated groups consistently formed larger colonies containing a higher number of hepatocyte-like cells per colony compared to controls. These results indicate that IR1 and IR2 may promote either the proliferation of successfully reprogrammed cells or the stability and expansion of hepatocyte-like cell populations within colonies. Regarding the differences in reprogramming efficiency between IR1 and IR2, we speculate that these may arise from differences in their inhibitory potency, selectivity, or downstream signaling effects, which could differentially influence the expansion or maintenance of reprogrammed hepatocyte-like cells. We have clarified these points in the revised manuscript and incorporated additional discussion to better explain these observations (page 11, lines 401-404). We thank the reviewer again for the valuable question.

13. The authors should assess expression of fibroblast identity markers following IRAK4 inhibition (for example Thy1, Postn, Col1a1, Col5a2) to directly demonstrate erasure of somatic identity.

Response: We thank the reviewer for this valuable suggestion. We assessed the mRNA expression of fibroblast identity markers (*Thy1*, *Postn*, *Col1a1*, and *Col5a2*). The results showed that CFAE+8 downregulated these fibroblast-associated genes more effectively than the positive control (CFAE+6). Furthermore, the addition of IRAK4 inhibitors (IR1 or IR2) did not further downregulate *Col1a1* or *Col5a2* mRNA levels compared to CFAE+8 or MEF. Both IR1 and IR2 also induced upregulation of *Postn* and downregulation of *Thy1*. Nevertheless, *Thy1* and *Postn* remained at low expression levels compared to MEF. Collectively, these results indicate that IRAK4 inhibition leads to complex or dual effects on the expression of fibroblast identity genes rather than complete erasure of somatic identity under our experimental conditions. We deleted this statement in revised manuscript (page 2 line 65).

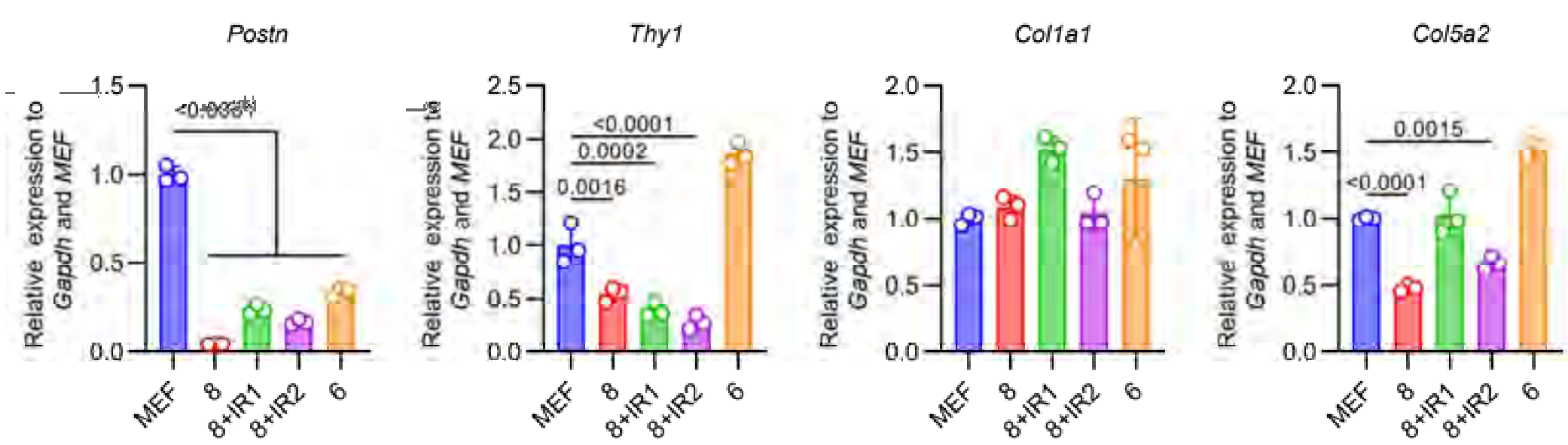

Response Figure 18 (**A**) and (**B**) Quantitative PCR of *Thy1, Postn, Col1a1, Col5a2* mRNA in MEF, CFAE+8, CFAE+8+IR1, CFAE+8+IR2, CFAE+6 samples. CFAE+6 was the positive control of XEN-like cells induction medium.

Minor comments

1. Line 191: “teems” should be corrected to “terms”.

Response: We thank the reviewer for this point. We replaced “teems” to “terms” in the revised manuscripts in line 155.

2. The statement that “erasure of somatic identity and initiation of the early reprogramming phase represent the principal rate limiting steps” is inaccurate. Early reprogramming and somatic identity loss occur efficiently in most cells, whereas activation of endogenous late pluripotency or lineage defining genes is generally rate limiting. This statement should be revised.

Response: We thank the reviewer for this important correction. We agree that our original statement was inaccurate and have revised it to reflect the current understanding of reprogramming dynamics. The revised text now states that "Activation of endogenous late pluripotency or lineage-defining genes represents the principal rate-limiting step". We appreciate the reviewer for bringing this to our attention and have updated the manuscript accordingly (page 2, lines 65-66).

Reference

1. Li, Y., Shah, R.B., Sarti, S., Belcher, A.L., Lee, B.J., Gorbatenko, A., Nemati, F., Yu, H., Stanley, Z., Rahman, M., et al. A noncanonical IRAK4-IRAK1 pathway counters DNA damage–induced apoptosis independently of TLR/IL-1R signaling. Science Signaling *16*, eadh3449. 10.1126/scisignal.adh3449.
2. Bennett, J., Ishikawa, C., Agarwal, P., Yeung, J., Sampson, A., Uible, E., Vick, E., Bolanos, L.C., Hueneman, K., Wunderlich, M., et al. (2023). Paralog-specific signaling by IRAK1/4 maintains MyD88-independent functions in MDS/AML. Blood *142*, 989-1007. 10.1182/blood.2022018718.
3. Hong, H., Takahashi, K., Ichisaka, T., Aoi, T., Kanagawa, O., Nakagawa, M., Okita, K., and Yamanaka, S. (2009). Suppression of induced pluripotent stem cell generation by the p53–p21 pathway. Nature *460*, 1132-1135. 10.1038/nature08235.
4. Kawamura, T., Suzuki, J., Wang, Y.V., Menendez, S., Morera, L.B., Raya, A., Wahl, G.M., and Belmonte, J.C.I. (2009). Linking the p53 tumour suppressor pathway to somatic cell reprogramming. Nature *460*, 1140-1144. 10.1038/nature08311.
5. Marión, R.M., Strati, K., Li, H., Murga, M., Blanco, R., Ortega, S., Fernandez-Capetillo, O., Serrano, M., and Blasco, M.A. (2009). A p53-mediated DNA damage response limits reprogramming to ensure iPS cell genomic integrity. Nature *460*, 1149-1153. 10.1038/nature08287.
6. Utikal, J., Polo, J.M., Stadtfeld, M., Maherali, N., Kulalert, W., Walsh, R.M., Khalil, A., Rheinwald, J.G., and Hochedlinger, K. (2009). Immortalization eliminates a roadblock during cellular reprogramming into iPS cells. Nature *460*, 1145-1148. 10.1038/nature08285.
7. Cheng, L., Wang, Y., Yang, Z., Cao, J., Peng, F., Lan, T., Cheng, R., Liu, T., Xia, Z., Guan, J., et al. (2026). p53 safeguards chemical reprogramming of human somatic cells toward pluripotency. Cell. 10.1016/j.cell.2026.03.038.
8. Wu, Y., Song, Y., Soto, J., Hoffman, T., Lin, X., Zhang, A., Chen, S., Massad, R.N., Han, X., Qi, D., et al. (2025). Viscoelastic extracellular matrix enhances epigenetic remodeling and cellular plasticity. Nature Communications *16*, 4054. 10.1038/s41467-025-59190-7.
9. Guan, J., Wang, G., Wang, J., Zhang, Z., Fu, Y., Cheng, L., Meng, G., Lyu, Y., Zhu, J., Li, Y., et al. (2022). Chemical reprogramming of human somatic cells to pluripotent stem cells. Nature. 10.1038/s41586-022-04593-5.
10. Wang, Y., Peng, F., Yang, Z., Cheng, L., Cao, J., Fu, X., He, H., Cai, R., Zeng, W., Dong, Y., et al. (2025). A rapid chemical reprogramming system to generate human pluripotent stem cells. Nat Chem Biol. 10.1038/s41589-024-01799-8.
11. Lee, J., Sayed, N., Hunter, A., Au, K.F., Wong, W.H., Mocarski, E.S., Pera, R.R., Yakubov, E., and Cooke, J.P. (2012). Activation of innate immunity is required for efficient nuclear

reprogramming. Cell *151*, 547-558. 10.1016/j.cell.2012.09.034.

12. Meng, S., Chanda, P., Thandavarayan, R.A., and Cooke, J.P. (2018). Transflammation: How Innate Immune Activation and Free Radicals Drive Nuclear Reprogramming. Antioxidants & Redox Signaling *29*, 205-218. 10.1089/ars.2017.7364.

Dear Prof. Li,

Thank you for the submission of your revised manuscript. We have now received the enclosed reports from the referees who agreed to assess it, and I am happy to say that both referees support the publication of your study now. However, both referees also point out that it remains unknown whether the changes in the cell cycle induced by IRAK4 knockdown mediate the increased reprogramming efficiency. This aspect needs to be noted and discussed more clearly in the ms file. For example, line 189 contains an overstatement. Please carefully revise the full ms text and remove all overstatements.

Please address all final referee comments in the ms text and please also co-submit a point-by-point response with your final ms files.

A few editorial requests will also need to be addressed:

- The conflict of interest subheading needs to be corrected to "Disclosure and Competing Interests Statement"

- There is one author name discrepancy - Chuanshu Huang in the ms vs. Huang Chuanshu in our online submission system. Please correct.

- The author credits need to be removed from the ms file. All credits need to be entered during online ms submission.

- The REFERENCE format is not correct. It needs to be alphabetical, not numerical; DOIs should only be used for preprints and datasets that have not been published yet. Please correct to EMBO reports reference style.

- DATA NOT SHOWN is mentioned on page 9. This is not allowed per journal policy, please re-write or show the data.

- 2 author checklists were uploaded. Please upload a single one only with your final ms.

- A callout for Figure 3E is missing, please add.

- Please note that all deposited data in public databases need to be freely accessible upon the online publication of your ms.

- Methods and Protocols should be only Methods.

- The Lead Contact section should be removed from p16.

- The Figure legends need to be placed after the References.

- Our routine image analysis detected a
*Reuse between Figure 1A and Appendix Fig S1 C,D&E that is Not listed in the figure legend. And a
*Reuse between Figure 2A and Appendix Fig S A,B&C that is Listed briefly in the figure legend. Please explain both re-uses and in case this is intentional create detailed callouts in the figure legends.

Figure Legends - Comments

- Please note that the exact p values are not provided in the legends of figures 1B-D, E-H; 2B-H; 6A-D; 7D-H; 8B, C; 9A, B, J. Please provide exact values as reasonable.
- Please indicate the statistical test used for data analysis in the legends of figures 3D, 4A, C, D; 7J, N; 8B, C; EV2 A-F; EV4 A-C;
- Please note that information related to n is missing in the legends of figures 3D, 7J, 8B, C; EV4 A-C
- Please note that the error bars are not defined in the legends of figures 8B, C; EV1 B, S1 D,

I would like to suggest some changes to the abstract that contains overstatements. Do you agree with this:

Chemical reprogramming holds transformative potential for regenerative medicine. However, the regulatory mechanisms governing cell fate transitions are not well understood. Here, we identify Interleukin-1 Receptor-Associated Kinase 4 (IRAK4) as a barrier to multi-lineage reprogramming. Pharmacological inhibition of IRAK4 enhances the reprogramming of mouse embryonic fibroblasts (MEFs) through a chemically activated multi-lineage priming (CaMP) state and extraembryonic endoderm (XEN)-like intermediates, increasing colony formation, and the expression of core XEN regulators (Sox17, Gata4, Sall4, and Foxa2). Genetic knockdown of Irak4 similarly accelerates reprogramming, whereas its overexpression blocks cell fate transitions. IRAK4 inhibition enhances chromatin accessibility and reshapes cell cycle dynamics, characterized by G0/G1

shortening and G2/M lengthening, potentially contributing to multi-lineage state establishment. Furthermore, IRAK4 suppression enhances the direct conversion of MEFs to neuron-like and hepatocyte-like cells, which exhibit enhanced functional maturity, including increased glycogen storage and improved detoxification capacity. Our findings establish IRAK4 as a regulator that constrains cellular plasticity potentially by coordinating chromatin accessibility and cell cycle dynamics.

EMBO press papers are accompanied online by A) a short (1-2 sentences) summary of the findings and their significance, B) 2-3 bullet points highlighting key results and C) a synopsis image that is exactly 550 pixels wide and 300 pixels high. The synopsis image should provide a sketch of the major findings, like a graphical abstract. Please note that text needs to be readable at the final size. Please send us this information along with the final manuscript.

I look forward to seeing a final version of your manuscript as soon as possible.

Best regards,
Esther

Esther Schnapp, PhD
Senior Editor
EMBO reports

Referee #1:

I thank the authors for their extensive revisions and for the substantial amount of new experimental work added to the manuscript. The newly included ATAC-seq analyses, TNF-α rescue experiments, neuronal differentiation assays, and the corresponding revisions to the text have significantly improved the study and strengthened several of the original conclusions.

In particular, I find that the authors have satisfactorily addressed my concerns regarding the mechanism linking IRAK4 inhibition to lineage conversion (Major Comment 3) and have provided functional evidence supporting the existence of a more plastic intermediate state (Major Comment 10). I also appreciate the authors' decision to moderate their conclusions regarding IRAK4 as a shared barrier across multiple reprogramming contexts (Major Comment 4), which now more accurately reflects the available evidence.

However, several points remain only partially resolved and should be further clarified in the revised manuscript.

1. Cell-cycle remodeling remains correlative rather than mechanistically established

In my original comments (Major Comments 1 and 2), I asked whether IRAK4 directly regulates cell-cycle machinery and whether cell-cycle remodeling is sufficient to account for the enhanced reprogramming phenotype.

The additional experiments demonstrate that disruption of cell-cycle progression impairs reprogramming, supporting the notion that proper cell-cycle progression is important for this process. However, these data do not establish that IRAK4 inhibition promotes reprogramming primarily through cell-cycle remodeling, nor do they provide direct molecular evidence linking IRAK4 activity to cell-cycle regulators.

Importantly, no analyses of canonical cell-cycle control pathways (e.g., cyclins, CDKs, RB phosphorylation, checkpoint kinases, mitotic markers, or DNA damage signaling) have been provided. Therefore, I recommend that the manuscript consistently present cell-cycle changes as an associated and potentially contributing mechanism rather than a demonstrated causal pathway.

2. Interpretation of immune signaling remains speculative

I appreciate the authors' thoughtful discussion regarding the possibility that immune-related gene activation following IRAK4 overexpression may reflect either inflammatory signaling or non-canonical IRAK4 functions.

However, the central question raised in my original comment remains unresolved: whether the observed immune gene induction is mechanistically involved in regulating reprogramming or simply represents a secondary consequence of IRAK4 overexpression.

The current response primarily relies on literature precedent and conceptual discussion rather than experimental evidence. Therefore, I suggest that the authors further moderate the relevant statements in both the Results and Discussion sections and explicitly acknowledge that the causal contribution of inflammatory signaling remains to be determined.

3. Clarification of the chromatin accessibility mechanism

The newly added ATAC-seq data are valuable and provide important mechanistic insight. However, the current data demonstrate a correlation between IRAK4 inhibition and increased chromatin accessibility rather than directly establishing causality.

I encourage the authors to avoid overly definitive language and instead frame chromatin accessibility remodeling as a plausible mechanism supported by the current evidence.

Overall assessment

Overall, the manuscript has been substantially improved and many of my original concerns have been adequately addressed. The remaining issues primarily concern the strength of mechanistic interpretation rather than the quality of the experimental data. I believe the manuscript would be considerably strengthened if the authors further align their conclusions with the level of evidence currently provided.

Referee #3:

The authors have added a significant amount of new data and addressed most of the concerns raised. While the manuscript is clearly improved, a key question regarding the role of the cell cycle in mediating the IRAK4 effect remains unresolved. Ultimately, it is for the editor to determine whether the absence of a clearly defined mechanism is sufficient for publication.

Dear Esther Schnapp,

We thank Referee #1 and Referee #3 for their positive assessment of our revisions and for their constructive suggestions to further improve the manuscript. We have carefully considered all remaining concerns and have revised the manuscript accordingly. Below is our point-by-point response.

Sincerely
Jun Li

Reviewers' comments:

Referee #1:

I thank the authors for their extensive revisions and for the substantial amount of new experimental work added to the manuscript. The newly included ATAC-seq analyses, TNF-α rescue experiments, neuronal differentiation assays, and the corresponding revisions to the text have significantly improved the study and strengthened several of the original conclusions.

In particular, I find that the authors have satisfactorily addressed my concerns regarding the mechanism linking IRAK4 inhibition to lineage conversion (Major Comment 3) and have provided functional evidence supporting the existence of a more plastic intermediate state (Major Comment 10). I also appreciate the authors' decision to moderate their conclusions regarding IRAK4 as a shared barrier across multiple reprogramming contexts (Major Comment 4), which now more accurately reflects the available evidence.

However, several points remain only partially resolved and should be further clarified in the revised manuscript.

We greatly appreciate the referee's recognition that our new experimental work has substantially strengthened the study. We also thank the referee for the clear guidance on aligning our conclusions with the level of evidence. We have addressed each point as follows.

1. Cell-cycle remodeling remains correlative rather than mechanistically established
In my original comments (Major Comments 1 and 2), I asked whether IRAK4 directly regulates cell-cycle machinery and whether cell-cycle remodeling is sufficient to account for the enhanced reprogramming phenotype.
The additional experiments demonstrate that disruption of cell-cycle progression impairs

reprogramming, supporting the notion that proper cell-cycle progression is important for this process. However, these data do not establish that IRAK4 inhibition promotes reprogramming primarily through cell-cycle remodeling, nor do they provide direct molecular evidence linking IRAK4 activity to cell-cycle regulators.

Importantly, no analyses of canonical cell-cycle control pathways (e.g., cyclins, CDKs, RB phosphorylation, checkpoint kinases, mitotic markers, or DNA damage signaling) have been provided. Therefore, I recommend that the manuscript consistently present cell-cycle changes as an associated and potentially contributing mechanism rather than a demonstrated causal pathway.

Response:

We fully agree with the referee's assessment. Our data show that IRAK4 inhibition is accompanied by changes in cell-cycle progression and that proper cell-cycle progression is important for reprogramming; however, we have not established a direct causal link between IRAK4 activity and core cell-cycle regulators. To address this concern, we have revised the manuscript throughout to present cell-cycle remodeling as an associated event that may contribute to the phenotype, rather than as a proven causal mechanism. Specific changes include:

1) **Results section:** The original sentence *"IRAK4 inhibition … thereby facilitating accelerated S-phase entry and mitotic progression to catalyze cell fate transitions"* has been revised to:
"Collectively, these integrated transcriptional and functional analyses demonstrate that despite acting on distinct primary molecular targets, both E-616452 and IRAK4 inhibitors converge functionally to alter cell cycle dynamics. These changes are associated with accelerated reprogramming kinetics, suggesting that cell-cycle remodeling may be an indispensable factor." (page 5-6, lines 191–195)

2) **Discussion section:** We have added a clear statement:
"While our data demonstrate a correlation between IRAK4 inhibition and cell-cycle changes, we cannot conclude that cell-cycle remodeling is the primary or direct mechanism underlying the enhanced reprogramming. Thus, we present cell-cycle changes as an associated and potentially permissive event rather than a demonstrated causal pathway." (page 10, lines 366–369)

2. Interpretation of immune signaling remains speculative

I appreciate the authors' thoughtful discussion regarding the possibility that immune-related gene activation following IRAK4 overexpression may reflect either inflammatory signaling or non-canonical IRAK4 functions.

However, the central question raised in my original comment remains unresolved: whether the observed immune gene induction is mechanistically involved in regulating reprogramming or simply represents a secondary consequence of IRAK4 overexpression. The current response primarily relies on literature precedent and conceptual discussion rather than experimental evidence. Therefore, I suggest that the authors further moderate the relevant statements in both the Results and Discussion sections and explicitly

acknowledge that the causal contribution of inflammatory signaling remains to be determined.

Response:

We agree with the referee that our current data do not resolve causality regarding immune-related gene activation. We have therefore further moderated the language in both the Results and Discussion sections, and we now explicitly acknowledge the uncertainty. Specific changes:

1) **Results section:** The original statement *"This pronounced pro-immunogenic shift … mechanistically aligns with our prior finding that CDK8 inhibitors … facilitate reprogramming by suppressing inflammatory signaling pathways"* has been revised to:

   "This pronounced pro-immunogenic shift in the transcriptional landscape is consistent with our prior observation that CDK8 inhibitors facilitate reprogramming by suppressing inflammatory signaling pathways. However, whether the immune-related gene activation upon IRAK4 overexpression plays a causal role in blocking reprogramming or merely represents a secondary consequence of enforced IRAK4 expression remains to be determined." (page 7, lines 260–264)

2) **Discussion section:** We have revised the paragraph beginning with *"The finding that IRAK4 overexpression actively promotes immune-associated gene signatures …"* to:

   "The finding that IRAK4 overexpression actively promotes immune-associated gene signatures reinforces the emerging concept that innate immune signaling pathways may act as guardians of somatic identity (Figure 7I). Their activation may reinforce a defensive cellular state resistant to identity change, while their suppression (via CDK8 or IRAK4 inhibition) licenses the cell for reprogramming by removing this molecular brake and altering cell cycle progression. However, whether this immune gene induction directly causes the reprogramming blockade or represents an epiphenomenon remains unclear, and additional studies are needed to establish causality." (page 11, lines 414–421)

3. Clarification of the chromatin accessibility mechanism

The newly added ATAC-seq data are valuable and provide important mechanistic insight. However, the current data demonstrate a correlation between IRAK4 inhibition and increased chromatin accessibility rather than directly establishing causality.

I encourage the authors to avoid overly definitive language and instead frame chromatin accessibility remodeling as a plausible mechanism supported by the current evidence.

Response:

We thank the referee for this important clarification. We have revised the manuscript to present chromatin accessibility remodeling as a plausible mechanism supported by correlation, not as a proven causal event. Specific changes:

1) **Results section:** The original sentence *"These results demonstrate that IRAK4 inhibition enhances chromatin accessibility at lineage development-associated genes"* has been revised to:
"Collectively, these results show that IRAK4 inhibition is associated with increased chromatin accessibility at lineage development-associated genes, raising the possibility that this chromatin remodeling contributes to the enhanced reprogramming." (page 6, lines 216–218)
2) **Discussion section:** We have added a clear statement:
"While our ATAC-seq data reveal a strong correlation between IRAK4 inhibition and increased chromatin accessibility, we cannot conclude that it is sufficient for the reprogramming phenotype. The observed accessibility changes provide a plausible mechanistic framework for future investigations, such as testing whether direct modulation of chromatin remodelers can phenocopy IRAK4 inhibition." (page 9, lines 339–343)

Overall assessment

Overall, the manuscript has been substantially improved and many of my original concerns have been adequately addressed. The remaining issues primarily concern the strength of mechanistic interpretation rather than the quality of the experimental data. I believe the manuscript would be considerably strengthened if the authors further align their conclusions with the level of evidence currently provided.

We are grateful for the referee's recognition that the manuscript has been substantially improved. Following the above revisions, we have aligned our conclusions with the level of evidence provided, as recommended. We hope that the revised manuscript now meets the referee's expectations.

Referee #3:

The authors have added a significant amount of new data and addressed most of the concerns raised. While the manuscript is clearly improved, a key question regarding the role of the cell cycle in mediating the IRAK4 effect remains unresolved. Ultimately, it is for the editor to determine whether the absence of a clearly defined mechanism is sufficient for publication.

Response:

We thank the referee for acknowledging the improvements and for raising this important point. We fully recognize that the exact mechanism by which IRAK4 influences the cell cycle and reprogramming is not yet fully resolved. As also suggested by Referee #1, we have now revised the manuscript to present cell-cycle changes as an associated and potentially contributing phenomenon rather than a proven causal pathway. The remaining

mechanistic questions are also clearly stated as such in the revised Discussion (see detailed changes in response to Referee #1).

2nd Revision - Editorial Decision 19th Jun 2026

Prof. Jun Li
Wenzhou medical university
School of Laboratory Medicine and Life Sciences
China

Dear Prof. Li,

I am very pleased to accept your manuscript for publication in the next available issue of EMBO reports. Thank you for your contribution to our journal.

Your manuscript will be processed for publication by EMBO Press. It will be copy edited and you will receive page proofs prior to publication. Please note that you will be contacted by Springer Nature Author Services to complete licensing and payment information.

You may qualify for financial assistance for your publication charges - either via a Springer Nature fully open access agreement or an EMBO initiative. Check your eligibility: https://link.springer.com/journal/44319/how-to-publish-with-us

Should you be planning a Press Release on your article, please notify the production team via this form in order to coordinate publication and release dates: https://forms.cloud.microsoft/e/eH4A8JVP6g

If you have any questions, please do not hesitate to contact the Editorial Office. Thank you for your contribution to EMBO Reports.

Best regards,
Esther

Esther Schnapp, PhD
Senior Editor
EMBO reports

>>> Please note that it is EMBO Reports policy for the transcript of the editorial process (containing referee reports and your response letter) to be published as an online supplement to each paper. If you do NOT want this, you will need to inform the Editorial Office via email immediately. More information is available here: https://link.springer.com/partners/embo-press/editorial-policies#Peer%20review

EMBO Press Author Checklist

Corresponding Author Name: Yang Zhao, Chuanshu Huang, Jun Li
Journal Submitted to: EMBO Reports
Manuscript Number: EMBOR-2025-62664V2

USEFUL LINKS FOR COMPLETING THIS FORM
The EMBO Journal - Author Guidelines
EMBO Reports - Author Guidelines
Molecular Systems Biology - Author Guidelines
EMBO Molecular Medicine - Author Guidelines

Reporting Checklist for Life Science Articles (updated January
This checklist is adapted from Materials Design Analysis Reporting (MDAR) Checklist for Authors. MDAR establishes a minimum set of requirements in transparent reporting in the life sciences (see Statement of Task: 10.31222/osf.io/9sm4x). Please follow the journal's guidelines in preparing your
Please note that a copy of this checklist will be published alongside your article.

Abridged guidelines for figures
1. Data
The data shown in figures should satisfy the following conditions:
- the data were obtained and processed according to the field's best practice and are presented to reflect the results of the experiments in an accurate and unbiased manner.
- ideally, figure panels should include only measurements that are directly comparable to each other and obtained with the same assay.
- plots include clearly labeled error bars for independent experiments and sample sizes. Unless justified, error bars should not be shown for technical
- if n<5, the individual data points from each experiment should be plotted. Any statistical test employed should be justified.
- Source Data should be included to report the data underlying figures according to the guidelines set out in the authorship guidelines on Data

2. Captions
Each figure caption should contain the following information, for each panel where they are relevant:
- a specification of the experimental system investigated (eg cell line, species name).
- the assay(s) and method(s) used to carry out the reported observations and measurements.
- an explicit mention of the biological and chemical entity(ies) that are being measured.
- an explicit mention of the biological and chemical entity(ies) that are altered/varied/perturbed in a controlled manner.
- the exact sample size (n) for each experimental group/condition, given as a number, not a range;
- a description of the sample collection allowing the reader to understand whether the samples represent technical or biological replicates (including how many animals, litters, cultures, etc.).
- a statement of how many times the experiment shown was independently replicated in the laboratory.
- definitions of statistical methods and measures:
  - common tests, such as t-test (please specify whether paired vs. unpaired), simple χ2 tests, Wilcoxon and Mann-Whitney tests, can be unambiguously identified by name only, but more complex techniques should be described in the methods section;
  - are tests one-sided or two-sided?
  - are there adjustments for multiple comparisons?
  - exact statistical test results, e.g., P values = x but not P values < x;
  - definition of 'center values' as median or average;
  - definition of error bars as s.d. or s.e.m.

Please complete ALL of the questions below.
Select "Not Applicable" only when the requested information is not relevant for your study.

Materials

Newly Created Materials	Information included in the manuscript?	In which section is the information available? (Reagents and Tools Table, Materials and Methods, Figures, Data Availability Section)
New materials and reagents need to be available; do any restrictions apply?	Not Applicable	

Antibodies	Information included in the manuscript?	In which section is the information available? (Reagents and Tools Table, Materials and Methods, Figures, Data Availability Section)
For **antibodies** provide the following information:<br>- Commercial antibodies: RRID (if possible) or supplier name, catalogue number and or/clone number<br>- Non-commercial: RRID or citation	Yes	Reagents and Tools Table, Methods

DNA and RNA sequences	Information included in the manuscript?	In which section is the information available? (Reagents and Tools Table, Materials and Methods, Figures, Data Availability Section)
**Short novel DNA or RNA including primers, probes**: provide the sequences.	Yes	Reagents and Tools Table

Cell materials	Information included in the manuscript?	In which section is the information available? (Reagents and Tools Table, Materials and Methods, Figures, Data Availability Section)
**Cell lines:** Provide species information, strain. Provide accession number in repository **OR** supplier name, catalog number, clone number, and/**OR** RRID.	Yes	Reagents and Tools Table, Methods, Materials and Methods
**Primary cultures:** Provide species, strain, sex of origin, genetic modification status.	Not Applicable	
Report if the cell lines were recently **authenticated** (e.g., by STR profiling) and tested for mycoplasma contamination.	Yes	Materials and Methods

Experimental animals	Information included in the manuscript?	In which section is the information available? (Reagents and Tools Table, Materials and Methods, Figures, Data Availability Section)
**Laboratory animals or Model organisms:** Provide species, strain, sex, age, genetic modification status. Provide accession number in repository **OR** supplier name, catalog number, clone number, **OR** RRID.	Yes	Materials and Methods
**Animal observed in or captured from the field:** Provide species, sex, and age where possible.	Yes	Materials and Methods
Please detail **housing and husbandry conditions**.	Not Applicable	

Plants and microbes	Information included in the manuscript?	In which section is the information available? (Reagents and Tools Table, Materials and Methods, Figures, Data Availability Section)
**Plants:** provide species and strain, ecotype and cultivar where relevant, unique accession number if available, and source (including location for collected wild specimens).	Not Applicable	
**Microbes:** provide species and strain, unique accession number if available, and source.	Not Applicable	

Human research participants	Information included in the manuscript?	In which section is the information available? (Reagents and Tools Table, Materials and Methods, Figures, Data Availability Section)
If collected and within the bounds of privacy constraints report on age, sex and gender or ethnicity for all study participants.	Not Applicable	

Core facilities	Information included in the manuscript?	In which section is the information available? (Reagents and Tools Table, Materials and Methods, Figures, Data Availability Section)
If your work benefited from core facilities, was their service mentioned in the acknowledgments section?	Yes	Materials and Methods

Design

Study protocol	Information included in the manuscript?	In which section is the information available? (Reagents and Tools Table, Materials and Methods, Figures, Data Availability Section)
If study protocol has been **pre-registered, provide DOI in the manuscript**. For clinical trials, provide the trial registration number **OR** cite DOI.	Not Applicable	
Report the **clinical trial registration number** (at ClinicalTrials.gov or equivalent), where applicable.	Not Applicable	

Laboratory protocol	Information included in the manuscript?	In which section is the information available? (Reagents and Tools Table, Materials and Methods, Figures, Data Availability Section)
Provide DOI OR other citation details if **external detailed step-by-step protocols** are available.	Yes	Materials and Methods

Experimental study design and statistics	Information included in the manuscript?	In which section is the information available? (Reagents and Tools Table, Materials and Methods, Figures, Data Availability Section)
Include a statement about **sample size** estimate even if no statistical methods were used.	Yes	Materials and Methods
Were any steps taken to minimize the effects of subjective bias when allocating animals/samples to treatment (e.g. **randomization procedure**)? If yes, have they been described?	Yes	Materials and Methods
Include a statement about **blinding** even if no blinding was done.	Not Applicable	
Describe **inclusion/exclusion criteria** if samples or animals were excluded from the analysis. Were the criteria pre-established?<br><br>If sample or data points were omitted from analysis, report if this was due to attrition or intentional exclusion and provide justification.	Not Applicable	
For every figure, are **statistical tests** justified as appropriate? Do the data meet the assumptions of the tests (e.g., normal distribution)? Describe any methods used to assess it. Is there an estimate of variation within each group of data? Is the variance similar between the groups that are being statistically compared?	Yes	Materials and Methods

Sample definition and in-laboratory replication	Information included in the manuscript?	In which section is the information available? (Reagents and Tools Table, Materials and Methods, Figures, Data Availability Section)
In the figure legends: state number of times the experiment was **replicated** in laboratory.	Yes	Materials and Methods, Figures
In the figure legends: define whether data describe **technical or biological replicates**.	Yes	Materials and Methods, Figures

Ethics

Ethics	Information included in the manuscript?	In which section is the information available? (Reagents and Tools Table, Materials and Methods, Figures, Data Availability Section)
Studies involving **human participants**: State details of **authority granting ethics approval** (IRB or equivalent committee(s), provide reference number for approval.	Not Applicable	
Studies involving **human participants**: Include a statement confirming that **informed consent** was obtained from all subjects and that the experiments conformed to the principles set out in the WMA Declaration of Helsinki and the Department of Health and Human Services Belmont Report.	Not Applicable	
Studies involving **human participants:** For publication of **patient photos**, include a statement confirming that consent to publish was obtained.	Not Applicable	
Studies involving experimental **animals**: State details of **authority granting ethics approval** (IRB or equivalent committee(s), provide reference number for approval. Include a statement of compliance with ethical regulations.	Not Applicable	
Studies involving **specimen and field samples:** State if relevant **permits** obtained, provide details of authority approving study; if none were required, explain why.	Not Applicable	

Dual Use Research of Concern (DURC)	Information included in the manuscript?	In which section is the information available? (Reagents and Tools Table, Materials and Methods, Figures, Data Availability Section)
Could your study fall under dual use research restrictions? Please check biosecurity documents and list of **select agents and toxins** (CDC): https://www.selectagents.gov/sat/list.htm	Not Applicable	
If you used a select agent, is the security level of the lab appropriate and reported in the manuscript?	Not Applicable	
If a study is subject to dual use research of concern regulations, is the name of the **authority granting approval and reference number** for the regulatory approval provided in the manuscript?	Not Applicable	

Reporting

The MDAR framework recommends adoption of discipline-specific guidelines, established and endorsed through community initiatives. Journals have their own policy about requiring specific guidelines and recommendations to complement MDAR.

Adherence to community standards	Information included in the manuscript?	In which section is the information available? (Reagents and Tools Table, Materials and Methods, Figures, Data Availability Section)
State if relevant guidelines or checklists (e.g., **ICMJE, MIBBI, ARRIVE, PRISMA**) have been followed or provided.	Not Applicable	
For **tumor marker prognostic studies,** we recommend that you follow the **REMARK** reporting guidelines (see link list at top right). See author guidelines, under 'Reporting Guidelines'. Please confirm you have followed these guidelines.	Not Applicable	
For **phase II and III randomized controlled trials**, please refer to the **CONSORT** flow diagram (see link list at top right) and submit the CONSORT checklist (see link list at top right) with your submission. See author guidelines, under 'Reporting Guidelines'. Please confirm you have submitted this list.	Not Applicable	

Data Availability

Data availability	Information included in the manuscript?	In which section is the information available? (Reagents and Tools Table, Materials and Methods, Figures, Data Availability Section)
Have **primary datasets** been deposited according to the journal's guidelines (see 'Data Deposition' section) and the respective accession numbers provided in the Data Availability Section?	Yes	Materials and Methods
Were **human clinical and genomic datasets** deposited in a public access-controlled repository in accordance to ethical obligations to the patients and to the applicable consent agreement?	Not Applicable	
Are **computational models** that are central and integral to a study available without restrictions in a machine-readable form? Were the relevant accession numbers or links provided?	Not Applicable	
If publicly available data were reused, provide the respective **data citations in the reference list**.	Not Applicable	
