# Supplementary material for: IRAK4 constrains cellular plasticity during chemically-induced cell fate reprogramming into multiple lineages
Source: EMBO Rep. 2026 Jul 15;27(16):4903–34. doi: 10.1038/s44319-026-00855-9 (PMC13503836; doi:10.1038/s44319-026-00855-9)
Supplement: Supplementary file 6 [file 44319_2026_855_MOESM4_ESM.zip › All microscopy images of of Figure 2A.docx]

All microscopy images of Figure 2A are collected in <https://www.ebi.ac.uk/biostudies/bioimages/studies/S-BIAD3324>
